# Supplementary material for: Functional Dissection of the Enhancer Repertoire in Human Embryonic Stem Cells
Source: Cell Stem Cell. 2018 Aug 2;23(2):276–288.e8. doi: 10.1016/j.stem.2018.06.014 (PMC6084406; doi:10.1016/j.stem.2018.06.014)

Cell Stem Cell, Volume *23*

Supplemental Information

Functional Dissection of the Enhancer Repertoire in Human Embryonic Stem Cells

Tahsin Stefan Barakat, Florian Halbritter, Man Zhang, André F. Rendeiro, Elena Perenthaler, Christoph Bock, and Ian Chambers

Supplementary Information

Functional dissection of the enhancer repertoire in human embryonic stem cells

Tahsin Stefan Barakat, Florian Halbritter, Man Zhang, André F. Rendeiro, Elena Perenthaler, Christoph Bock and Ian Chambers

Supplemental Inventory

Supplemental Figure legends

Supplemental Figures:

Figure S1, related to Figure 1: ChIP-seq in primed H9 human embryonic stem cells
Figure S2, related to Figure 1: Overview of generated datasets
Figure S3, related to Figure 2: ChIP-STARR-seq in primed H9 ESCs
Figure S4, related to Figure 4: Core and extended enhancer module
Figure S5, related to Figure 5: Conversion of primed to naive H9 human embryonic stem cells
Figure S6, related to Figure 5: ChIP-STARR-seq and functional enhancers in naive ESCs
Figure S7, related to Figure 7: Super-enhancers and ChIP-STARR-seq in primed and naive ESCs

Supplemental Figure Legends

Figure S1, related to Figure 1: ChIP-seq in primed H9 human embryonic stem cells

A) Brightfield microscopy of a representative colony of H9 ESCs cultured on Matrigel in standard ESC culture conditions.

B) Immunofluorescence of primed H9 ESCs for NANOG (green) or OCT4 (red); DNA is stained with DAPI (blue).

C) ChIP-qPCR in primed H9 ESC with anti-NANOG, anti-OCT4 or rabbit IgG at known OCT4 and NANOG binding sites in *SCGB3A2*, *SMARCA* (Kunarso et al., 2010) and *XIST* (left) or with anti-H3K4me1, anti-H3K27ac or rabbit IgG at binding sites near *FGFR1* (at central and flanking locations), *POU5F1* (at central and flanking locations), *CD9*, *SCGB3A2*, and *SMARCA* (Rada-Iglesias et al., 2011) (right). The mean fold-enrichment is shown relative to total input control DNA, normalized to a non-bound site in *ACTB* (for OCT4 and NANOG), or *NCAPD2* (for H3K4me1 and H3K27ac). Error bars indicate standard deviations; n= 3.

D) Venn diagrams of the overlap between ChIP-seq peaks in primed ESCs, indicating the cell line and study for NANOG, OCT4, H3K4me1 and H3K27ac (Ji et al., 2016; Gafni et al., 2013; Gifford et al., 2013; Kunarso et al., 2010; Lister et al., 2009 and this study) . The numbers indicate overlapping peaks.

E) Heatmap contrasting gene expression of immune response genes in human ESCs as determined by RNA-seq (Muerdter et al., 2018). Data from (Gifford et al., 2013; Ji et al., 2016; Takashima et al., 2014)

Figure S2, related to Figure 1: Overview of generated datasets

A) Summary table of the high-throughput sequencing datasets generated in this study, indicating the number of ChIP-seq, plasmid (corresponding to ChIP-STARR-seq or BAC-STARR-seq plasmid libraries prior to transfection) and isolated plasmid (plasmid libraries 24h after transfection) samples, as well as RNA-seq data from GFP-positive primed and/or naive ESCs (RNA: primed and RNA: naive, respectively).

B) Pearson correlation matrix of samples from the indicated ChIP-seq, plasmid libraries and isolated plasmid libraries from primed ESCs. Rows and columns have been arranged by hierarchical clustering with complete linkage.

C-D) Scatterplots contrasting normalized read counts (reads per million) per peak for different datasets. The Pearson correlation coefficient (*r*) for each comparison is indicated for each plot.

C) Comparison between ChIP-seq, DNA-seq of ChIP-STARR-seq plasmid libraries and two replicates of DNA-seq for isolated plasmid libraries post transfection, for OCT4, NANOG, H3K37ac, H3K27ac and genomic DNA (input).

D) Comparison between ChIP-STARR-seq plasmid libraries prior to transfection and the corresponding RNA-seq read counts generated from two replicates of GFP-positive cells after transfection.

E) Pearson correlation coefficients (*r*) between replicate STARR-RNA-seq measurements from the same pool of ESCs transfected with the same ChIP-STARR-seq library, shown as a function of minimum read count in both replicates (0 = unfiltered, 1 = at least one read from the same plasmid measured in each replicate, etc.).

Figure S3, related to Figure 2: ChIP-STARR-seq in primed H9 ESCs

A) Bar graph showing the mean GFP intensity as measured by flow cytometry for positive or negative ChIP-STARR-seq regions cloned into STARR-seq plasmids and used in transfection of H9 primed ESCs. Average results for two experiments are shown. Three different minimal promoters were assessed for each tested sequence. GFP mean intensity was indistinguishable from non-transfected cells for negative sequences in all constructs.

B) Boxplots showing the distribution of RNA-seq RPKM values of genes associated with enhancers grouped by activity level. Boxplots represent the interquartile range (IQR), the line is the median, whiskers extend to 1.5xIQR and outliers are indicated as dots. The numbers on the x-axis indicate thresholds on the RPP activity level; RPKM, reads per kilobase million. RNA-seq datasets were from the following studies: H1 (Ji et al., 2016); HUES64 (Gifford et al., 2013).

C) Distribution of active (RPP ≥138) and inactive sequences (RPP <138) in ChIP-STARR-seq regions overlapping with ChIP-seq peaks of the indicated factor or combination of factors.

D) PCR genotyping of H9 ESC wild type (WT) and targeted clones (numbers), that were transfected with Cas9 and gRNAs to delete DNA sequences with enhancer activity detected by ChIP-STARR-seq (*pos*, top) or inactive (*neg*, bottom) sequences. The putative interacting gene is indicated. Primers used for genotyping are located outside the gRNA targets; wt = wt allele, ko = knockout allele.

E) Relative enrichment of DNA-binding proteins (DBPs) from the ENCODE database (2012) in inactive genome regions, as well as active enhancers (compare to **Fig. 2G**). Shown are the log_2-odds ratios between observed percentages of enhancers overlapping binding sites of each given DBP in the respective groups over the percentage of overlaps in the entire enhancer dataset. Each dot represents one ChIP-seq dataset for the given DBP and the lines connect the most extreme dot with zero for visualization. For each category, the eight most enriched

DBPs are shown ranked by their mean log-odds ratio. ChIP-seq datasets produced from ESCs are indicated as dots and those from other cell sources as crosses. Enrichments were calculated using LOLA (Sheffield and Bock, 2016).

F) Smooth line plots showing the proportion of active plasmids (RPP ≥138) of all plasmids measured at the indicated distance from the peak center for ChIP-seq binding sites of factors from the CODEX database (Sanchez-Castillo et al., 2015) found preferentially associated at highly active enhancers (compare to **Fig. 2H**), averaged across all binding sites for the respective factor. The number of peaks (*n*) is indicated in each plot.

G) LOLA enrichment plots as in panel **E**, but showing instead the relative over-presentation of ENCODE chromatin segments from different cell lines in enhancers with different activity levels. E, enhancer; PF, promoter-flanking region; R, repressed; T, transcribed; TSS, transcription start site; WE, weak enhancer.

H) Line plots as in panel F, showing the proportion of active plasmids in a window around the center of repressed chromatin segments and enhancer chromatin segments from the ENCODE H1 chromatin segmentation (Hoffman et al., 2013).

Figure S4, related to Figure 4: Core and extended enhancer module

A) Illustration showing three source datasets (Hawkins et al., 2011; Rada-Iglesias et al., 2011; Xie et al., 2013) that contributed to the catalogue of putative ESC enhancers used in this study. We converted all enhancer coordinates to the same assembly (hg19) and then merged overlapping peaks resulting in a list of 76,666 putative enhancers.

B) Luciferase assay in primed ESCs for eight putative enhancers that did not show activity in ChIP-STARR-seq. The OCT4 proximal enhancer (PE) is tested as a positive control. Luciferase activity is reported as the fold enrichment in luciferase counts over empty vector, normalised to the Renilla transfection control. Error bars indicate standard deviations, n=2.

C) Illustration of the number of genes found in the proximity of core enhancer module (pink, left circle), or extended enhancer module (olive, right), or with enhancers of both types (white, overlap). Enhancer-gene assignments were performed with GREAT (McLean et al., 2010).

D) Receiver operating characteristic (ROC) curve illustrating random forest classifier performance.

E) Bar charts displaying the scaled variable importance of the top 25 sequence features used for the distinction of active enhancers from inactive regions. Motif IDs are shortened versions of the full ID from the HOCOMOCO database (v11) (Kulakovskiy et al., 2016). dinuc., dinucleotide frequency.

F) Boxplots of gene expression (RNA-Seq; $\log_2$) in different tissues from the GTEx database (2013)for all genes linked to Core (pink) or Extended (olive) module enhancers or both (white). Boxplots represent the interquartile range (IQR), the line is the median, whiskers extend to 1.5xIQR and outliers are indicated as dots. RPKM, reads per kilobase million.

Figure S5, related to Figure 5: Conversion of primed to naive H9 human embryonic stem cells

A) Brightfield microscopy of a representative colony of H9 ESCs cultured in naive culture conditions on feeders for 10 passages showing a more dome-shaped colony morphology (compare to **Figure S1A**).

B) Immunofluorescence of naive H9 ESCs for NANOG (green) and OCT4 (red); DNA is stained with DAPI (blue).

C) Immunoblot analysis of NANOG and LAMININ B in primed and naive ESCs.

D) qRT-PCR of pluripotency-related genes in H9 ESCs cultured in primed or naive conditions (10 passages). Error bars indicate standard deviations; n=3.

E) ChIP-qPCR in naive H9 ESC with anti-NANOG, anti-OCT4 or rabbit IgG at three OCT4 and NANOG binding sites (from primed ChIP-seq data) in *SCGB3A2, SMARCA* (Kunarso et al., 2010) and *XIST* (left) or with anti-H3K4me1, anti-H3K27ac or rabbit IgG at binding sites near *FGFR1* (at central and flanking locations), *POU5F1* (at central and flanking locations), *CD9*, *SCGB3A2* and *SMARCA* (Rada-Iglesias et al., 2011) (right). The mean fold-enrichment is shown relative to total input control DNA, normalized to a non-bound site in *ACTB* (for NANOG and OCT4) or *NCAPD2* (for H3K4me1 and H3K27ac). Error bars indicate standard deviations; n= 3.

F-G) Venn diagrams of the overlap between ChIP-seq peaks in naive ESCs, indicating the cell line and study for **F)** H3K4me1 and **G)** H3K27ac (Gafni et al., 2013 and this study). The numbers indicate overlapping peaks.

H) Local motif enrichment analysis using CentriMo (Bailey and Machanick, 2012) for OCT4 ChIP-seq data generated in this study for primed (upper panel) and naive H9 ESCs (lower panel). Top-3 identified motifs and their p-values are indicated.

I) as **H,** but now for NANOG.

Figure S6, related to Figure 5: ChIP-STARR-seq and functional enhancers in naive ESCs

A-B) Scatterplots contrasting normalized read counts (reads per million) in naive H9 ESCs per peak for different datasets. The Pearson correlation coefficient (*r*) for each comparison is indicated for each plot.

A) Comparison of ChIP-seq datasets and the corresponding ChIP-STARR-seq plasmid library.

B) Comparison between ChIP-STARR-seq plasmid libraries prior to transfection and the corresponding RNA-seq read counts generated from two replicates of GFP-positive naive H9 ESCs after transfection.

C) Plot showing enhancer activity (normalized ratio of ChIP-STARR RNA over plasmids; $\log_2$) in naive H9 ESCs ranked from lowest to highest across all measured enhancers (union of all peak calls). Active enhancers and inactive regions are discriminated by a threshold (θ) as indicated in the plot by a dashed line. RPP, reads per plasmid million.

D) Distribution of active (RPP $\geq$138) and inactive sequences (RPP <138) in naive ESCs in ChIP-STARR-seq regions overlapping with ChIP-seq peaks of the indicated factor or combination of factors.

E) Relative enrichment of DNA-binding proteins (DBPs) from the ENCODE database (2012) in inactive genome regions, as well as lowly active and highly active enhancers in naive H9 ESCs (compare to **Fig. 5B**). Shown are the $\log_2$-odds ratios between observed percentages of enhancers overlapping binding sites of each given DBP in the respective groups over the percentage of overlaps in the entire enhancer dataset. Each dot represents one ChIP-seq dataset for the given DBP and the lines connect the most extreme dot with zero for visualization. For each category, the eight most enriched DBPs are shown ranked by their mean log-odds ratio. ChIP-seq datasets produced from ESCs are indicated as dots and those

from other cell sources as crosses. Enrichments were calculated using LOLA (Sheffield and Bock, 2016).

F) LOLA enrichment plots as in panel **C**, but showing instead the relative over-presentation of ENCODE chromatin segments from different cell lines in enhancers with different activity levels. E, enhancer; PF, promoter-flanking region; R, repressed; T, transcribed; TSS, transcription start site; WE, weak enhancer.

G) Barplots showing relative enrichment of H9 chromatin segment overlaps (Kundaje et al., 2015) in regions with ChIP-STARR-seq activity compared to inactive regions (see panel **A**). TSS, transcription start site; enh, enhancer; ZNF, zinc-finger protein.

H) Density scatter plot comparing ranked activity (RPP) in primed and naive ESCs. Point density is represented by color and contours are shown to emphasize dense regions. The majority of points is located either in the lower left (inactive) or upper left (active in primed and naive) section of the plot.

I) Plot equivalent to panel **A**, but showing ChIP-seq binding intensity instead of RPP. A change point analysis was performed to determine a threshold to distinguish strongly bound from weakly bound regions. RPM, reads per million.

J) Table showing the percentage of ChIP-STARR-seq regions with a certain activity (e.g., Inactive in primed and naive ESCs) that remain weakly bound (W→W), gain binding (W→S), lose binding (S→W), or remain strongly bound (S→S) in the transition from primed to naive ESCs according to the threshold shown in panel G. W, weak ChIP-seq binding; S, strong ChIP-seq binding; I, inactive ChIP-STARR-seq region; A, active ChIP-STARR-seq enhancer.

K) Functional enrichment analysis using Enrichr to test the relative over-representation of enhancers that lose ChIP-STARR-seq activity in the transition from primed to naive ESCs (Active→Inactive), that gain activity (Inactive→Active), or that remain inactive in both states

(Inactive→Inactive). Enrichments were calculated for genes near these regions to test for GO assignments (left) or for ENCODE and ChEA ChIP-seq experiments (right). The x-axis reports the combined score calculated by Enrichr.

L) Distribution of active (RPP ≥138) and inactive sequences (RPP <138) in naive ESCs in ChIP-STARR-seq regions of the Core module (top), Extended module (middle), or in regions that were inactive in primed ESCs. The p-value calculated by the χ^2 test is indicated.

M) Receiver operating characteristic (ROC) curve illustrating random forest classifier performance. The area under the curve (AUC) and Gini index are reported.

Figure S7, related to Figure 7: Super-enhancers and ChIP-STARR-seq in primed and naive ESCs

A) Super-enhancers in naive H9 ESCs (SEs) were called from H3K27ac ChIP-seq data on enhancers stitched within 12.5kb windows using the ROSE software (Whyte et al., 2013).

B) Scatterplot contrasting SE intensity in naive H9 ESCs (H3K27ac signal divided by input) with ChIP-STARR-seq activity. The Pearson correlation coefficient (*r*) is indicated and the red line represents a generalised additive model fit to the data.

C) Kernel density plots showing the distribution of SE intensity values (H3K27ac signal divided by input) in primed and naive H9 ESCs.

D) Scatterplot contrasting SE intensity values (H3K27ac signal divided by input) in primed and naive H9 ESCs. Regions called as SEs in primed, naive, or both ESCs are indicated in blue, red, or purple, respectively.

E) Comparison between super-enhancers and H3K27ac peaks from H1 ESCs (Hnisz et al., 2013) and this study.

F) Genome browser plots showing two regions captured by our additional BAC-STARR-seq libraries. Shown are annotated genes in these regions, the BAC-covered region itself (in yellow), followed by tracks showing the combined ChIP-seq coverage and RNA over plasmid ratios in primed and naive ESCs.

G) PCR genotyping of H9 wild type (WT) and targeted clones (numbers) that were transfected with Cas9 and gRNAs to delete three different constituents of the *FGFR1* super-enhancer (part A, B and G, see **Figure 7**). Primers used for genotyping are located outside the gRNA targets; wt = wt allele, ko = knockout allele.

H) qRT-PCR analysis of wild type (wt) H9 ESCs or H9 ESCs with a homozygous deletion of *FGFR1* super-enhancer part A, part B or part G for amplicons detecting *FGFR1*, *LETM2, C8ORF86*, *RNF5P1* and *WHSC1L1* (*NSD3*) mRNA. 3 clones per genotype were assessed, and

expression was normalized for *TBP* and one wt set to 1. *** =$p<0.001$ (2-way ANOVA with Bonferoni post-test), error bars are SD.

I) Violin plots showing the proportion of active plasmids (RPP $\geq$ 138) for 1,159 super-enhancers (SE) compared to normal enhancers (NE) in naive ESCs.

J) Violin plots as in panel I, for SEs and NEs in primed (left) and naive (right) ESCs with super-enhancers based on H3K27ac peaks with d=0kb stitching distance (called using ROSE).

Supplemental Figures

Figure S1
Related to Figure 1

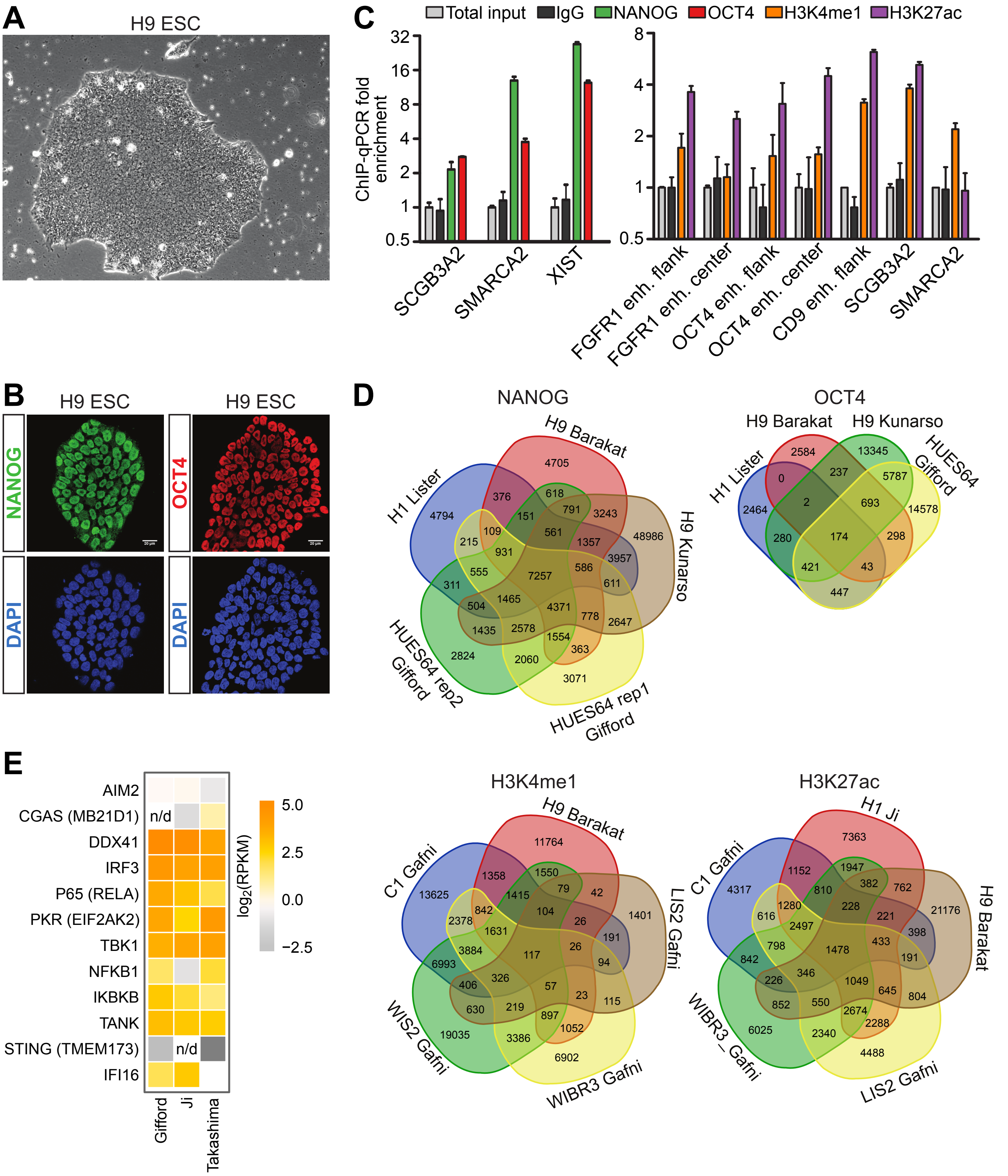

Figure S2
Related to Figure 1

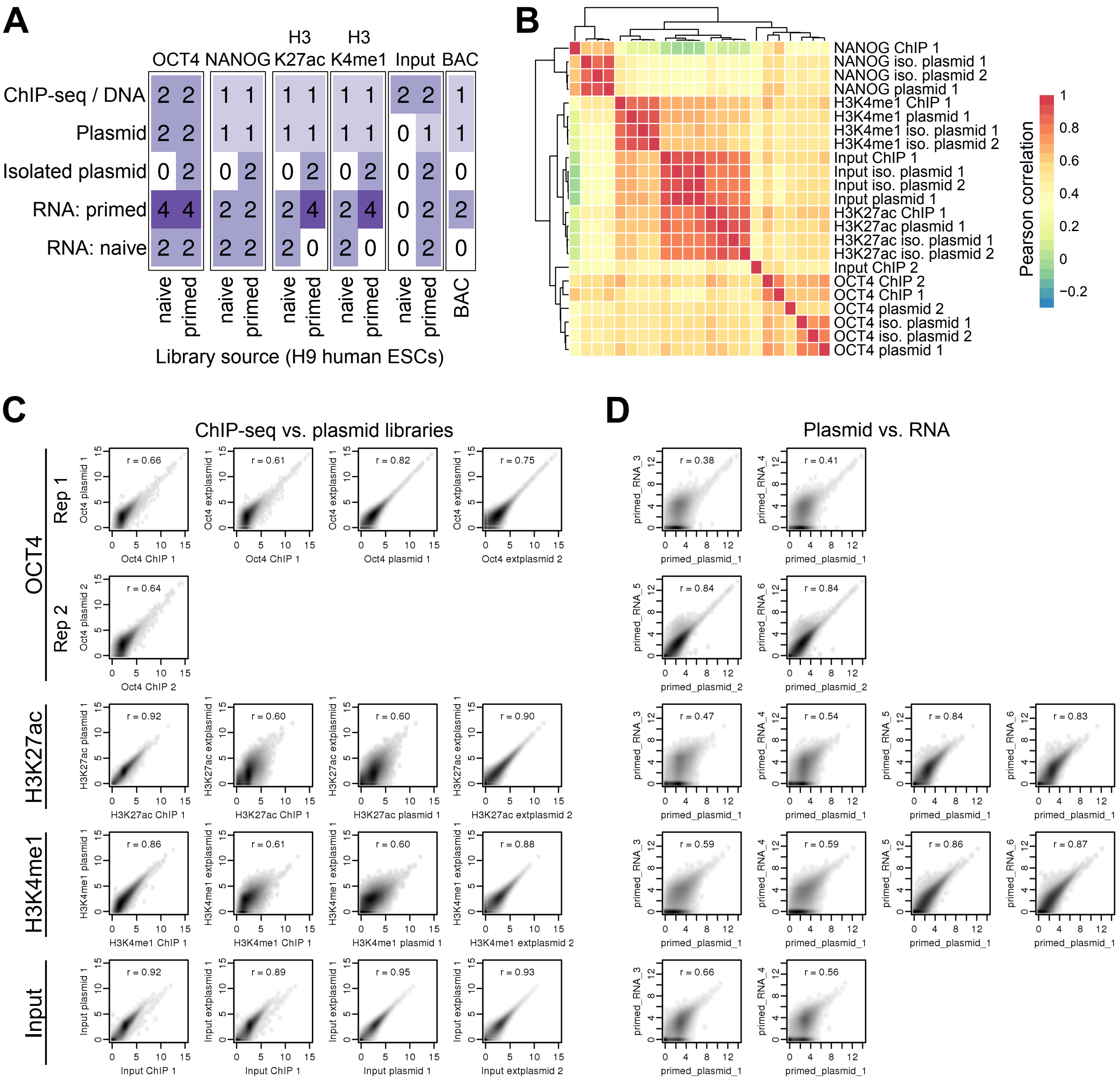

Figure S3
Related to Figure 2

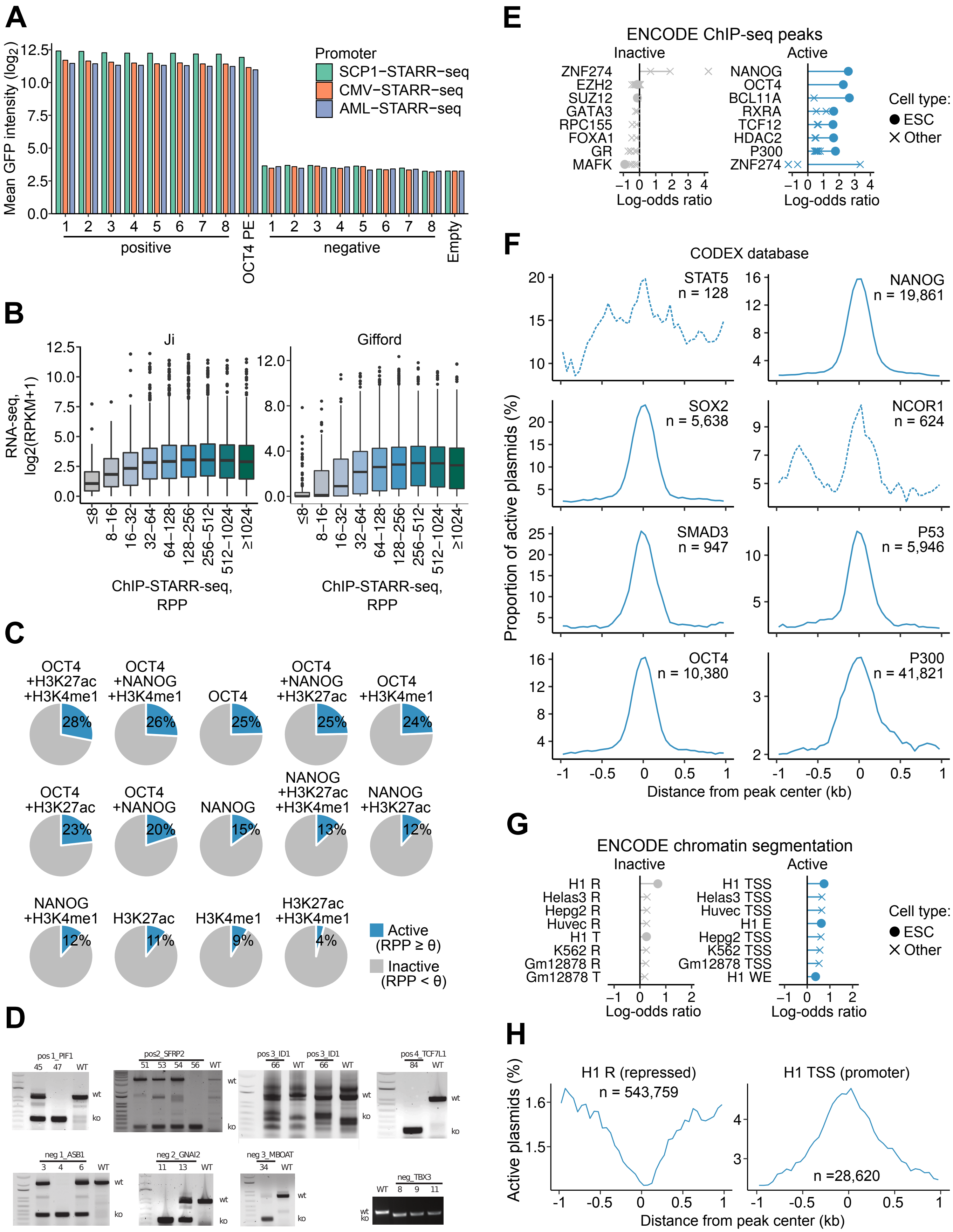

Figure S4
Related to Figure 4

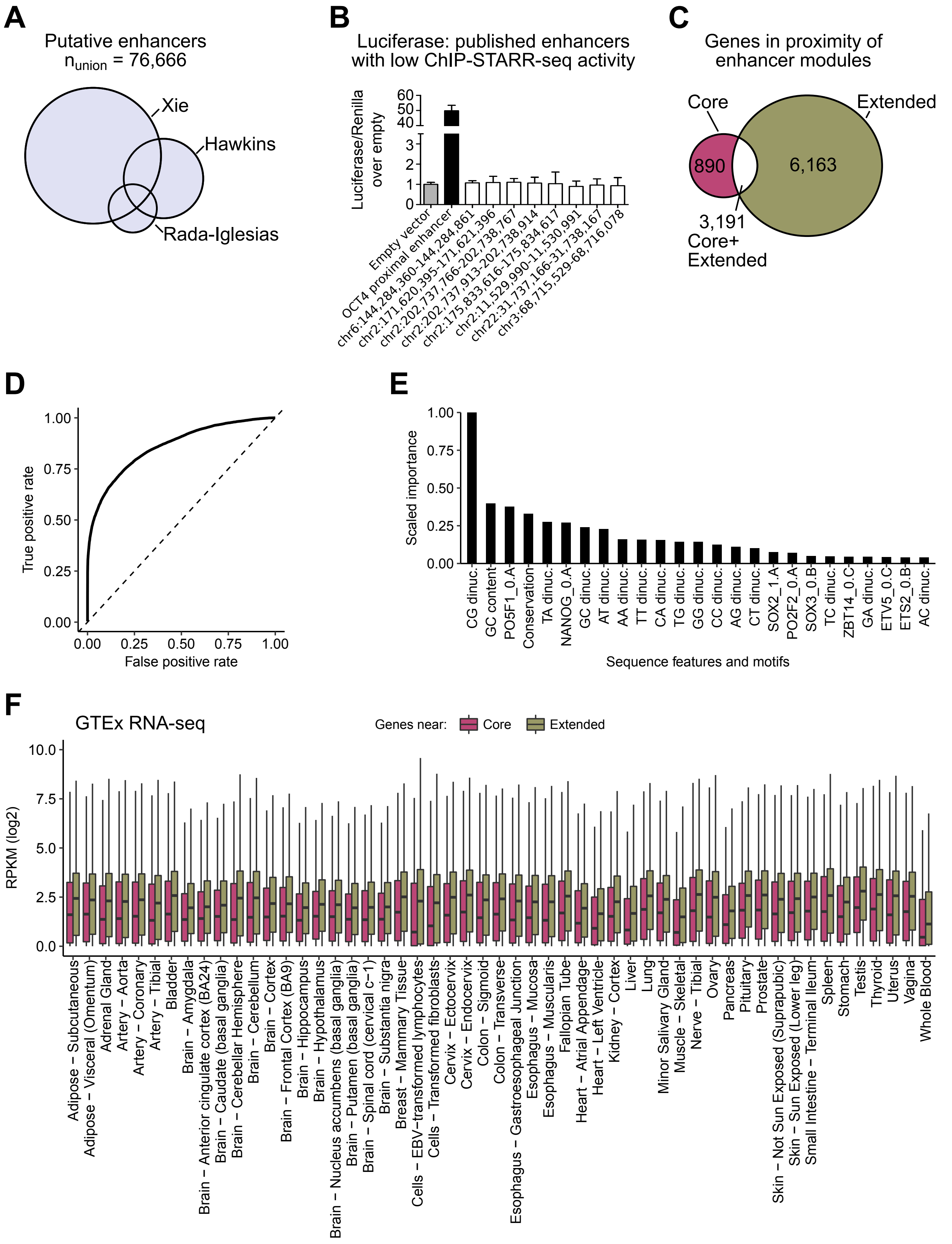

Figure S5
Related to Figure 5

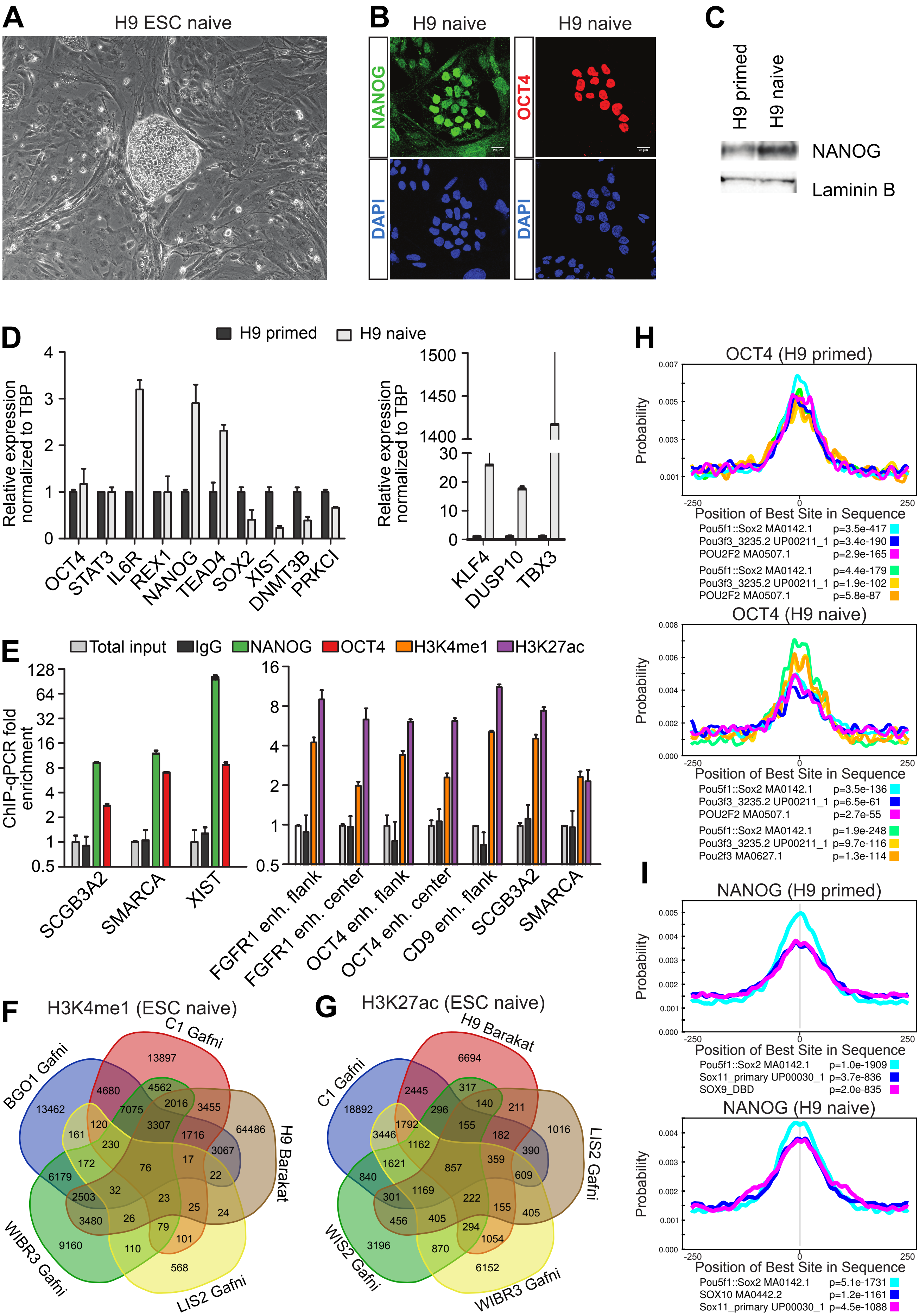

Figure S6
Related to Figure 5

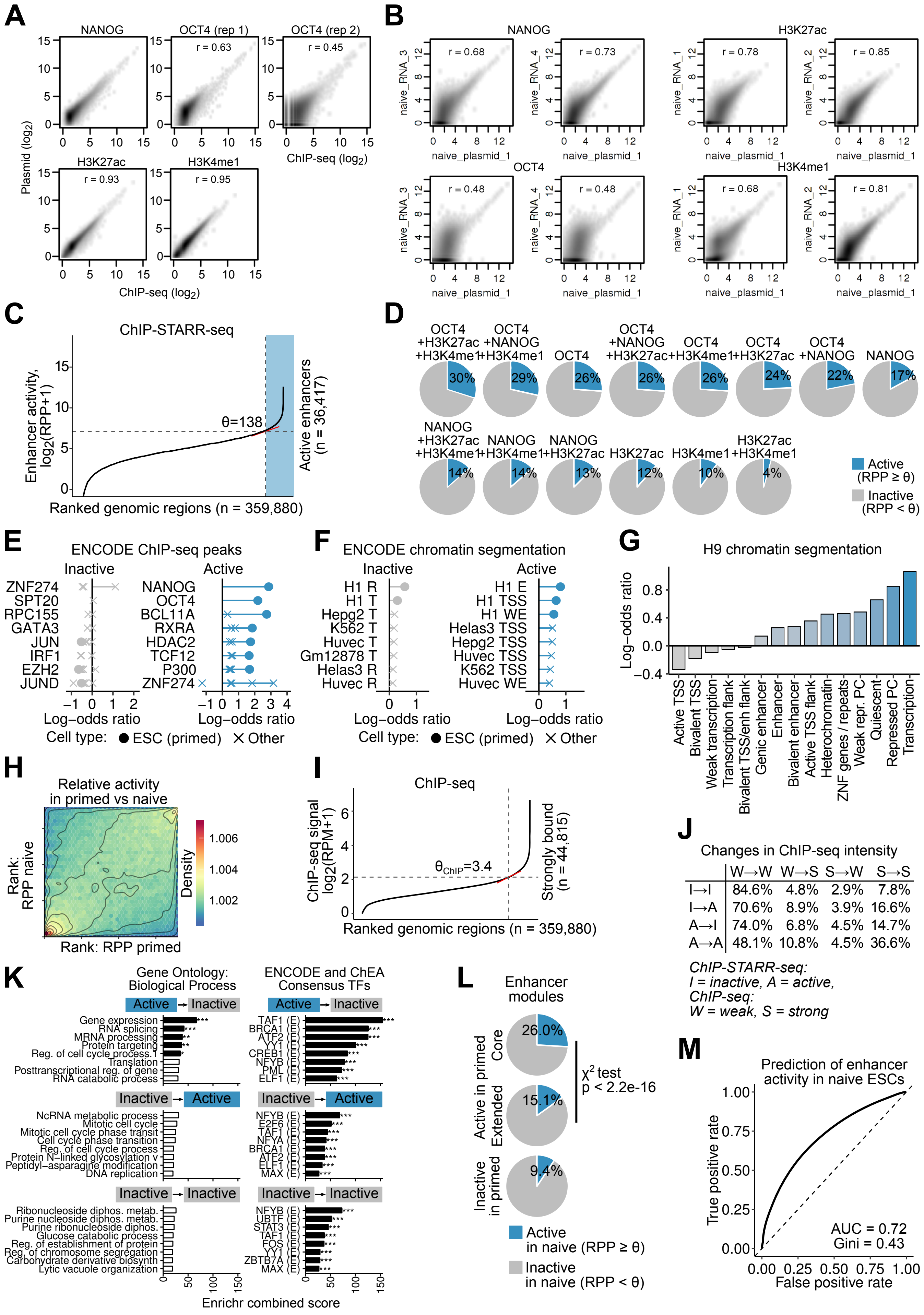

Figure S7
Related to Figure 7

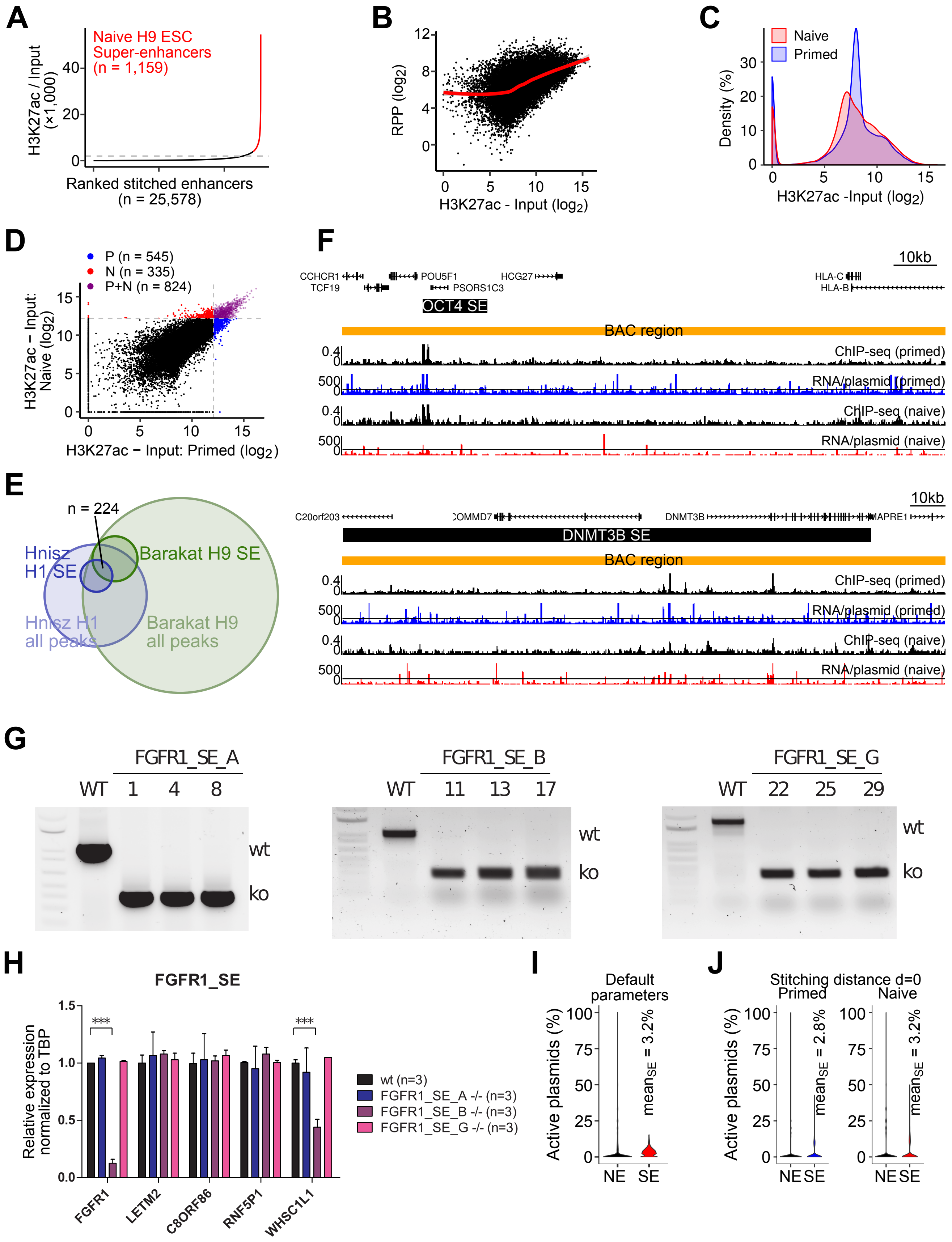

Supplement: Document S1. Figures S1–S7 [file mmc1.pdf]
