# Supplementary material for: Functional Dissection of the Enhancer Repertoire in Human Embryonic Stem Cells
Source: Cell Stem Cell. 2018 Aug 2;23(2):276–288.e8. doi: 10.1016/j.stem.2018.06.014 (PMC6084406; doi:10.1016/j.stem.2018.06.014)

Cell Stem Cell

Resource

Functional Dissection of the Enhancer Repertoire in Human Embryonic Stem Cells

Graphical Abstract

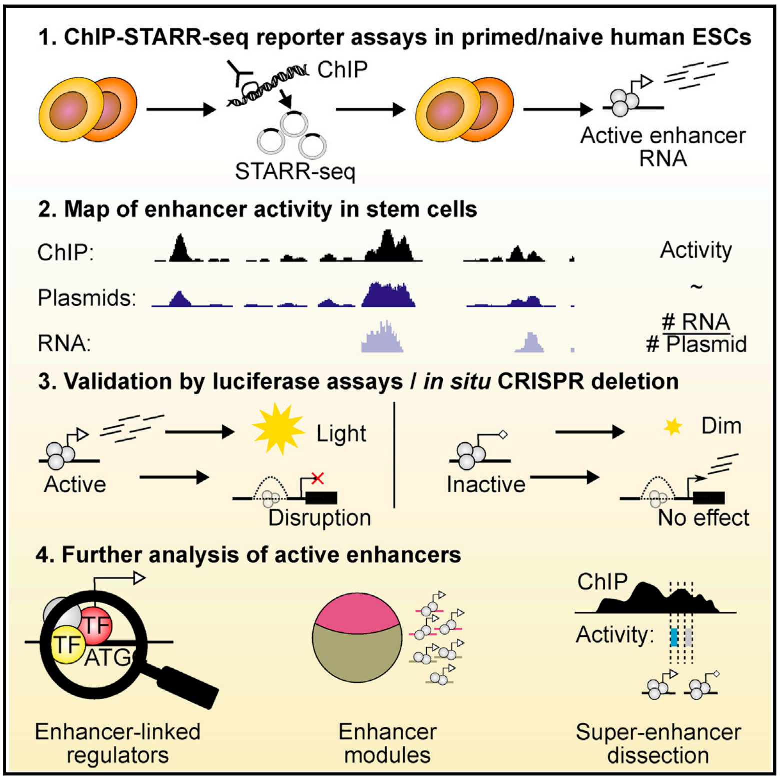

Highlights

- Massively parallel reporter assay assessed over 350,000 genome regions
- ChIP-STARR-seq catalogs functional enhancers in primed and naive hESCs
- Identification of transcription factors and transposable elements linked to enhancers
- ChIP-STARR-seq dissects super-enhancers into small functional units

Authors

Tahsin Stefan Barakat, Florian Halbritter, Man Zhang, André F. Rendeiro, Elena Perenthaler, Christoph Bock, Ian Chambers

Correspondence

t.barakat@erasmusmc.nl (T.S.B.), i.chambers@ed.ac.uk (I.C.)

In Brief

Barakat et al. use a combination of chromatin immunoprecipitation and a massively parallel reporter assay to identify functional enhancers in primed and naive human embryonic stem cells. This genome-wide catalog of validated enhancers provides a valuable resource for the further dissection of the regulatory genome.

Barakat et al., 2018, Cell Stem Cell *23*, 276–288
August 2, 2018 © 2018 The Authors. Published by Elsevier Inc.
https://doi.org/10.1016/j.stem.2018.06.014

Cell Stem Cell
Resource

Functional Dissection of the Enhancer Repertoire in Human Embryonic Stem Cells

Tahsin Stefan Barakat,[1,2,6,*] Florian Halbritter,[3,6] Man Zhang,[1,7] André F. Rendeiro,[3,7] Elena Perenthaler,[2] Christoph Bock,[3,4,5] and Ian Chambers[1,8,*]
[1]MRC Centre for Regenerative Medicine, Institute for Stem Cell Research, School of Biological Sciences, University of Edinburgh, Edinburgh, EH16 4UU, UK
[2]Department of Clinical Genetics, Erasmus MC, University Medical Center, Wytemaweg 80, 3015 CN Rotterdam, the Netherlands
[3]CeMM Research Center for Molecular Medicine of the Austrian Academy of Sciences, Lazarettgasse 14, AKH BT 25.3, 1090 Vienna, Austria
[4]Department of Laboratory Medicine, Medical University of Vienna, 1090 Vienna, Austria
[5]Max Planck Institute for Informatics, Saarland Informatics Campus, 66123 Saarbrücken, Germany
[6]These authors contributed equally
[7]These authors contributed equally
[8]Lead Contact
*Correspondence: t.barakat@erasmusmc.nl (T.S.B.), i.chambers@ed.ac.uk (I.C.)
https://doi.org/10.1016/j.stem.2018.06.014

SUMMARY

Enhancers are genetic elements that regulate spatiotemporal gene expression. Enhancer function requires transcription factor (TF) binding and correlates with histone modifications. However, the extent to which TF binding and histone modifications functionally define active enhancers remains unclear. Here, we combine chromatin immunoprecipitation with a massively parallel reporter assay (ChIP-STARR-seq) to identify functional enhancers in human embryonic stem cells (ESCs) genome-wide in a quantitative unbiased manner. Although active enhancers associate with TFs, only a minority of regions marked by NANOG, OCT4, H3K27ac, and H3K4me1 function as enhancers, with activity markedly changing under naive versus primed culture conditions. We identify an enhancer set associated with functions extending to non-ESC-specific processes. Moreover, although transposable elements associate with putative enhancers, only some exhibit activity. Similarly, within super-enhancers, large tracts are non-functional, with activity restricted to small sub-domains. This catalog of validated enhancers provides a valuable resource for further functional dissection of the regulatory genome.

INTRODUCTION

Human embryonic stem cells (ESCs) are a genetically tractable developmental model system with potential for stem-cell-based therapeutics. Understanding how ESC pluripotency is regulated by transcription factors (TFs) is central to achieving this promise. Gene expression is modulated by *cis*-regulatory elements, such as enhancers (Banerji et al., 1981), which can stimulate target gene expression in a position and orientation-independent manner, independent of their genomic context (Spitz and Furlong, 2012). ESCs direct a specific gene expression program using a network of TFs including OCT4, SOX2, and NANOG. Compared to mouse ESCs, human ESCs are more developmentally advanced with characteristics of post-implantation embryos. Recently, so-called naive ESCs with pre-implantation embryo characteristics have been derived from established ESCs either by transient transgene expression (Buecker et al., 2010; Hanna et al., 2010; Takashima et al., 2014) or by altering culture conditions (Gafni et al., 2013; Theunissen et al., 2014). Naive ESCs differ from primed ESCs in several ways, including increased clonogenicity, different growth factor requirements, distinct energy metabolism, and altered morphology (Sperber et al., 2015), but how naive and primed ESCs differ in enhancer usage is currently unclear.

The past decade of genomics research has focused on cataloguing *cis*-regulatory elements within the non-coding genome (ENCODE Project Consortium, 2012). Technological advances have allowed genome-wide occupancy by TFs to be measured by chromatin immunoprecipitation (ChIP) followed by sequencing (ChIP-seq). Putative enhancer locations have been obtained by mapping histone modifications (e.g., H3K27ac, H3K4me1) (Rada-Iglesias et al., 2011) and by measuring chromatin accessibility (Buenrostro et al., 2013). However, not all predicted enhancers could be validated functionally. To assay enhancer activity, plasmid-based cell transfections can be used. Recent advances have enabled thousands of sequences to be tested simultaneously (Kwasnieski et al., 2012; Melnikov et al., 2012; Patwardhan et al., 2012). For instance, with self-transcribing active regulatory region sequencing (STARR-seq) compact, non-mammalian genomes can be quantitatively screened for enhancer activity by cloning randomly sheared DNA between a minimal-promoter-driven GFP open reading frame and a downstream polyA sequence. If an enhancer is active, this results in transcription of the enhancer sequence (Arnold et al., 2013). Similar approaches have been adapted to test chosen sequences with putative enhancer features (Kwasnieski et al., 2014; Vanhille et al., 2015), predicted TF binding sites (Verfaillie et al., 2016), features of quantitative trait loci (Tewhey et al., 2016), or nucleosome-depleted sequences (Murtha et al., 2014).

 © 2018 The Authors. Published by Elsevier Inc.
This is an open access article under the CC BY license (http://creativecommons.org/licenses/by/4.0/).

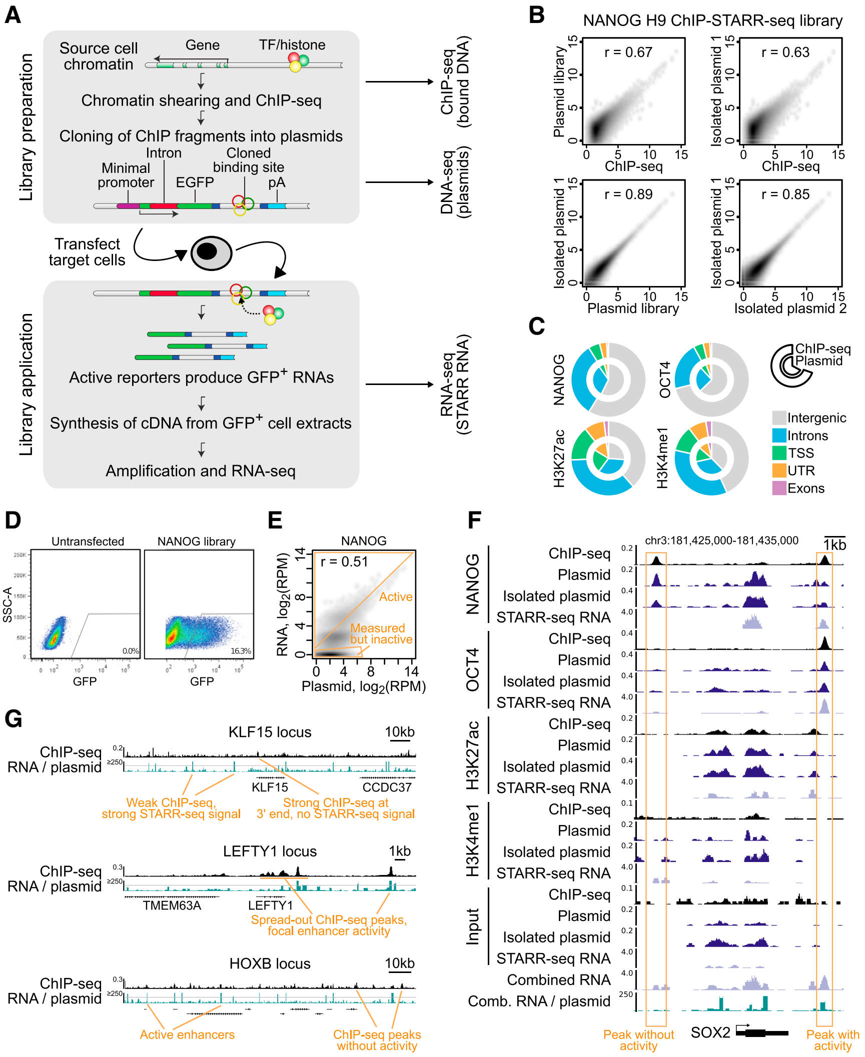

Figure 1. ChIP-STARR-Seq in Human Embryonic Stem Cells

(A) Outline of the ChIP-STARR-seq approach combining antibodies against TFs or histone modifications (colored balls) with the STARR-seq plasmid (Arnold et al., 2013).

(B) ChIP-STARR-seq for NANOG in H9. Scatterplots compare normalized read count (reads per million) per peak between datasets, obtained from ChIP-seq or DNA-seq of plasmid libraries pre- or post-transfection/recovery from ESCs (n = 2); *r*, Pearson correlation.

(C) Genomic distribution of peaks called for ChIP-seq (outer chart) and corresponding plasmid libraries (inner chart). TSSs, transcription start sites.

(D) FACS plots of single DAPI-negative ESCs. Left: untransfected cells; right: cells transfected with a NANOG ChIP-STARR-seq plasmid library.

(E) Scatterplot (like in B) comparing the NANOG plasmid library and corresponding ChIP-STARR-seq RNA. The dense cluster of points in the lower left corresponds to library plasmids that did not produce RNAs. RPM, reads per million.

(F) Genome browser plot of *SOX2* showing tracks for ChIP-seq, DNA-seq of plasmid libraries pre- and post-transfection, and from RNA-seq of GFP+ cells transfected with the indicated libraries. Bottom: combination (maximum) of all STARR-seq RNA-seq tracks and ratio of normalized RNA-seq/plasmid reads.

(G) Genome browser shots of *KLF15, LEFTY*, and *HOXB* cluster, illustrating a broad variety of enhancers profiled in this functional enhancer catalog.

Application of STARR-seq to explore mammalian genomes is hindered by genome size which means enhancer sequences would be infrequently sampled. This issue can be alleviated by combining ChIP with STARR-seq (Vockley et al., 2016). Using a similar approach (that we refer to as "ChIP-STARR-seq"), we generate a resource of genome-wide activity maps of functional enhancers in ESCs. This identifies highly active enhancers with major changes in activity patterns between primed and naive ESCs. Moreover, some transposable element (TE) families are enriched at highly active enhancers. Our data also identify the functional components within super-enhancers (SEs) and uncover a previously unidentified set of enhancers, including some associated with housekeeping functions. This resource encompasses an extensive collection of functional enhancer sequences in ESCs, providing a knowledge base for systematic analysis of the transcriptional circuitry underlying ESC maintenance and differentiation. Enhancer data are available from the STAR Methods and from a resource website (http://hesc-enhancers.computational-epigenetics.org).

RESULTS

ChIP-STARR-Seq: An Effective Strategy for Genome-wide Identification of Functional Enhancers

To generate a catalog of genomic elements that regulate ESC biology, we used a massively parallel reporter assay called "ChIP-STARR-seq." In ChIP-STARR-seq, DNA is co-immunoprecipitated and cloned *en masse* within the transcription unit of a STARR-seq plasmid that is downstream of GFP driven by a minimal promoter and upstream of a polyA sequence (Figure 1A) (Arnold et al., 2013). The resultant libraries can be tested for enhancer activity by cell transfection. If a cloned sequence functions as an enhancer, the transfected GFP-positive cells can be purified by fluorescence-activated cell sorting (FACS). Since the assayed sequences lie upstream of the polyA signal, the transcribed mRNA will contain the enhancer sequence. Therefore, both the identity and activity of captured regions can be determined quantitatively by sequencing mRNA (RNA-seq) from GFP-positive cells.

To investigate the functional potential of enhancers in ESCs, we first focused on primed H9 ESCs (Figures S1A and S1B)

and performed ChIP for NANOG, OCT4, H3K4me1 and H3K27ac. ChIP-qPCR and ChIP-seq were similar to previous results (Figures S1C and 1D). Although plasmid transfection can elicit an immune response in some cell types (Muerdter et al., 2018), the low expression of STING and CGAS in H1 (Muerdter et al., 2018) and H9 (Figure S1E) suggests this does not apply to ESCs. ChIP-STARR-seq libraries were generated (see the STAR Methods). Sequencing precipitated DNA, plasmid libraries, and transcribed RNAs produced 2.7×10^9 reads in total. Each plasmid library consisted of 8.4–30.8 $\times$ 10^6 unique plasmids, with a mean insert size of 221 bp (Table S1). Figure S2A summarizes the sequenced samples analyzed in this study.

We first assessed whether the plasmid libraries achieved a good representation of the binding events captured by ChIP-seq (Data S1). A good correlation between ChIP-seq coverage and the corresponding plasmid libraries was seen both pre- and post-transfection (Figures 1B, 1C, S2B, and S2C). Next, the ability of the plasmid libraries to drive GFP expression in primed ESCs was tested. Library transfections produced up to 20% GFP-positive cells compared to <1% GFP-positive cells obtained by transfection of the empty STARR-seq vector or ~50% cherry-positive in control transfections with a constitutively expressed mCherry plasmid (Figure 1D; data not shown). Therefore, a considerable proportion of cells contained plasmids with enhancer activity. 24 hr post-transfection, DNA was prepared from unsorted cells and RNA from FACS-purified GFP-positive cells was amplified for RNA-seq. DNA sequencing confirmed high consistency between the original plasmid libraries and plasmids re-isolated post-transfection (Figures 1B and S2C). Positive correlations were also observed between read coverage from STARR-RNA-seq and the respective plasmid libraries (Figures 1E and S2D) and between replicate STARR-RNA-seq datasets, with an increase for expressed plasmids sampled in replicates (mean correlation $r = 0.77$ at read count ≥ 5). These results show that while abundant plasmids can produce more RNA, some plasmids produce RNA in excess of the plasmid count, indicating high enhancer activity. However, many plasmids transfected into cells did not produce RNA indicating that the ChIP-enriched DNA in these plasmids lacked enhancer activity.

Visual inspection of selected genomic regions illustrates the broad spectrum of enhancer activity measured by ChIP-STARR-seq (Figures 1F and 1G). For instance, ChIP-seq for NANOG indicates two strong binding sites up- and downstream of *SOX2* (Figure 1F), but only the downstream binding site resulted in ChIP-STARR-seq RNA in excess of plasmid abundance.

Activity Levels Define Classes of Enhancers Bound by Distinct Transcription Factors

Using ChIP-STARR-seq, we assessed the functional capacity of 361,737 genomic regions in primed ESCs (Table S2). Enhancer activity was defined as the ratio of RNA reads relative to plasmid reads after normalization (RPP, reads per plasmid). Paired-end sequencing enabled unequivocal assignment of RNA reads to plasmids. The activity level of each region was recorded as the activity generated by the most active plasmid (from any library) within this region. The activities of 68 genomic regions covering the full activity range were compared with luciferase-based assays, and included regions covered in ChIP-seq and evaluated as not active in the STARR-seq assay. DNAs from regions of < 64 RPP had luciferase activities indistinguishable from empty vector. In contrast, regions with increasingly high ChIP-STARR-seq activity showed gradually higher luciferase activity (Figure 2A). Using different minimal promoters did not affect the activity calls of selected regions (Figure S3A). To assess the relationship of activity classifications to gene expression, each region was assigned to a putative target gene based on genomic distance. ChIP-STARR-seq regions with enhancer activity were associated with genes that showed significantly higher gene expression values than genes associated with regions lacking enhancer activity (Figures 2B and S3B). To simplify further analysis and ease interpretation, we defined thresholds for discriminating genuine enhancer activity from the activity of the minimal promoter in the STARR-seq by examining mathematical changepoints in the ranked curve of RPP values (Figure 2C). The greatest changepoint ($\theta \geq 138$) was taken as the threshold to define active enhancers. Based on these thresholds, ChIP-STARR-seq identified 32,353 active enhancers (Figure 2C; Data S1).

Applying this threshold to regions bound by NANOG, OCT4, H3K4me1, H3K27ac, or combinations of these factors indicates that only a minority of ChIP-seq peaks showed enhancer activity (Figure 2D and S3C), with regions bound by OCT4 having the highest proportion of high activity enhancers. To determine whether activity predictions from the plasmid-based assay identified enhancers functional at the endogenous loci, ESCs with deletions of regions exhibiting or lacking STARR-seq activity were engineered using CRISPR-Cas9 (Figures 2E and S3D). Changes in gene expression at each locus were observed only for the target gene and only when an active element was deleted. Removal of inactive regions was without effect.

The endogenous context of assessed regions was examined by comparing our data to public reference datasets starting with the H9 chromatin segmentation (Kundaje et al., 2015) (Figure 2F). Chromatin segments marked as enhancers, transcription start sites (TSSs), sites flanking transcription and repeat sequences were most overrepresented in active regions. The relative representation of TFs from 190 ChIP-seq datasets from CODEX was next assessed by LOLA enrichment analysis (Sánchez-Castillo et al., 2015; Sheffield and Bock, 2016) (Figure 2G; Table S3). High activity enhancers were preferentially associated with pluripotency-related TFs (SOX2, SMAD3, OCT4, and NANOG). Overlaps were also seen for regions bound in non-ESCs by STAT5 and NCOR1. In contrast, no TFs were enriched at inactive regions. Similar results were obtained by extending the analysis to 690 ChIP-seq datasets for TFs from ENCODE Project Consortium (2012) (Figure S3E). Enhancer activity was strongest close to the binding peaks of enriched factors with activity lost quickly with increasing distance from the peak center (Figure 2H and S3F). These results suggest that binding of distinct TFs in close proximity may contribute to robust enhancer activity. How enhancer classes relate to chromatin state was further examined by LOLA analysis of ENCODE chromatin segmentations from H1 ESCs and various non-pluripotent cell types (Figures S3G and S3H). This confirmed that active enhancers were enriched in segments annotated as H1 enhancers and promoters, while inactive regions occurred primarily in closed

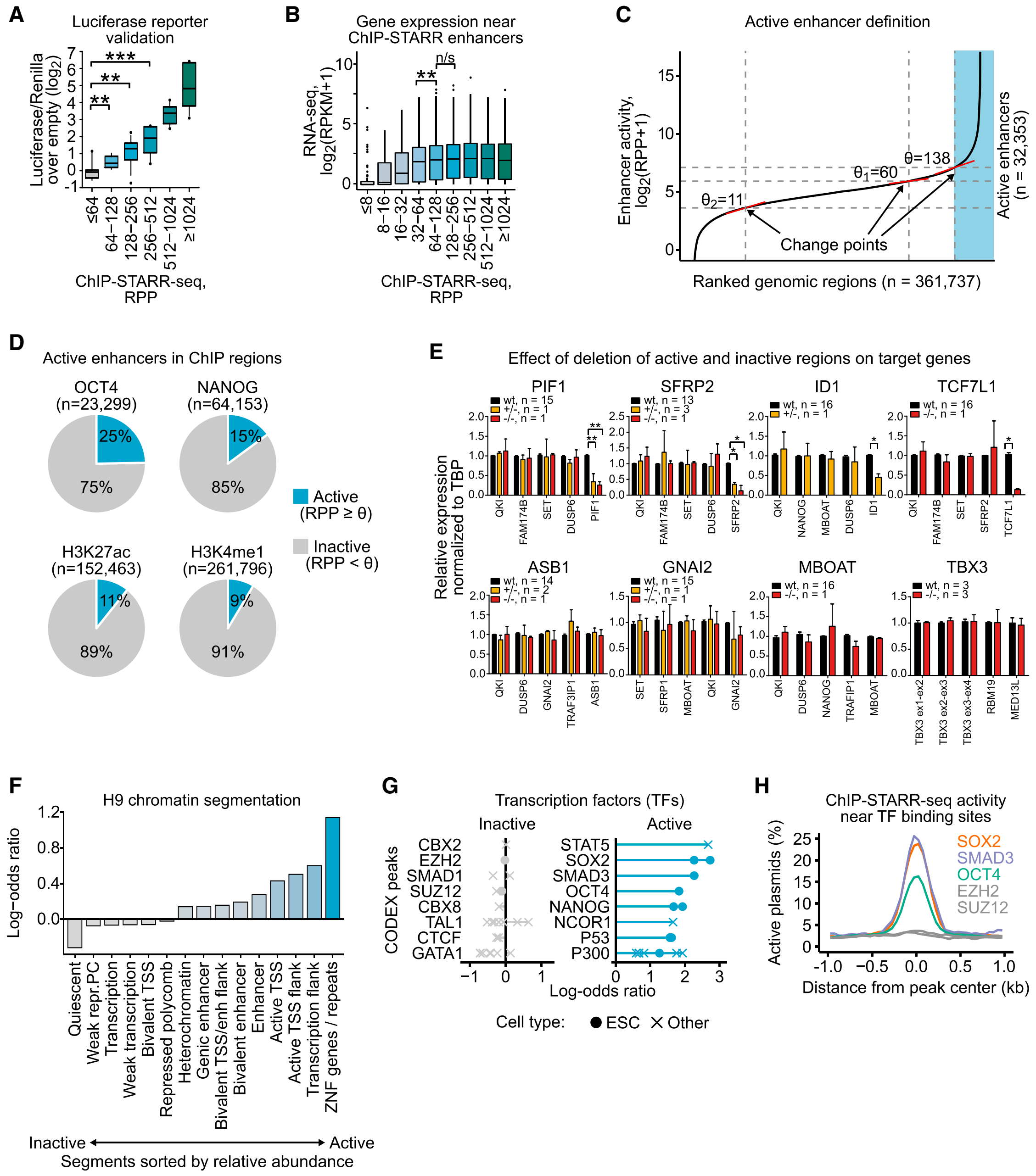

Figure 2. Activity Levels Define Functional Classes of Enhancers

(A) Luciferase activities of 68 genomic sequences in primed ESCs grouped by ChIP-STARR-seq activity. Boxes are interquartile range (IQR); line is median; and whiskers are the 10th to the 90th percentile. *p < 0.05; **p < 0.01; ***p < 0.001; Mann-Whitney test; n = 2.

(B) Distribution of expression values (Takashima et al., 2014) of genes associated with enhancers grouped by activity level. Boxes are IQR; line is median; whiskers extend to 1.5× the IQR; and dots are outliers. **p < 0.01; ***p < 0.001; unpaired t test.

(C) Plot showing enhancer activity (enrichment of ChIP-STARR-seq RNA over plasmids; $\log_2$) ranked from lowest to highest across all measured enhancers (union of all peak calls). Enhancers were distinguished based on activity; dashed lines indicate thresholds (θ).

(D) Distribution of active (RPP ≥ 138) and inactive sequences (RPP < 138) in peaks called for the indicated factors.

(E) qRT-PCR analysis of wild-type (WT) and enhancer-deleted heterozygous (+/−) or homozygous (−/−) ESC clones. Indicated mRNAs are normalized to TBP (WT = 1), and the average results for the indicated deletions are plotted relative to wild-type; n = number of cell lines per genotype (see STAR Methods for further details). *p < 0.05; **p < 0.01; ***p < 0.001 (two-way ANOVA, Bonferroni post-test). Error bars represent SD.

(F) Relative enrichment of H9 chromatin segment overlaps (Kundaje et al., 2015) between regions with ChIP-STARR-seq activity and inactive regions (see C).

(G) Relative LOLA enrichment of TFs from CODEX (Sánchez-Castillo et al., 2015) in inactive regions and active enhancers. Odds ratios between observed frequencies of enhancers overlapping binding sites for the eight most enriched TFs in the respective groups relative to the percentage in the entire region set are shown, ranked by mean odds ratio. Each dot represents a TF ChIP-seq dataset. ChIP-seq datasets from non-ESCs are shown as crosses.

(H) Smooth line plots of the proportion of active plasmids (RPP ≥ 138) around the peak center for the indicated ChIP-seq binding sites.

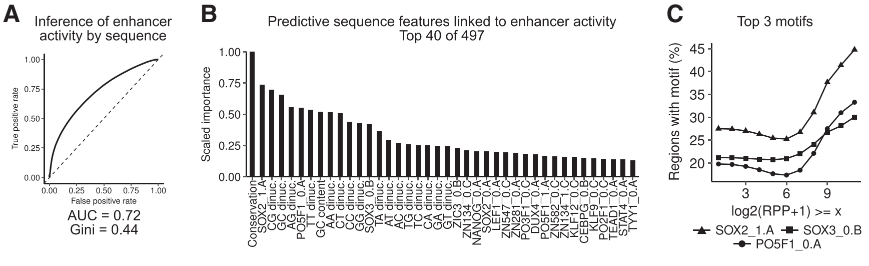

Figure 3. Sequence Determinants of Enhancer Activity

(A) Receiver operating characteristic (ROC) curve of the random forest classifier performance. AUC, area under the curve.

(B) The top-40 sequence features used to distinguish active and inactive regions ordered by variable importance. HOCOMOCO motif IDs were shortened (Kulakovskiy et al., 2016).

(C) Line plots of the percentage of regions containing one of the top-3 motifs from HOCOMOCO as a function of enhancer activity. Each point is the fraction of regions with at least $\log_2$(RPP+1) also containing the respective motif.

chromatin. Together, these results indicate that ChIP-STARR-seq can distinguish ChIP-seq peaks on the basis of enhancer activity and that enhancer activity reflects expression and regulatory function at the endogenous loci.

Sequence Determinants of Enhancer Activity

To address what distinguishes active enhancers from inactive regions, we used a machine learning approach to train a classifier to discriminate both types of regions based on sequence features (conservation, GC content, dinucleotide frequencies) and TF binding motif occurrence (see the STAR Methods). Mediocre classifier performance was achieved (AUC = 0.72; Figure 3A). The most informative features for enhancer activity were sequence conservation, ESC-related TF binding motifs occurrence, and various dinucleotide frequencies (Figure 3B), in line with recent observations from other MPRA data (Kreimer et al., 2017). The top-3 enriched TFs were found in higher abundance at regions with increasing RPP (Figure 3C). Our analysis highlights sequence features influencing enhancer activity but indicates that computational analysis with the simple features assessed could not unequivocally predict activity.

Active ESC Enhancers Include an Extended Module Containing Enhancers Associated with Housekeeping Functions

High-throughput sequencing studies have attempted to predict ESC enhancers on the basis of histone marks, TF binding, or DNaseI hypersensitivity (Hawkins et al., 2011; Rada-Iglesias et al., 2011; Xie et al., 2013). However, the overlap between enhancers predicted from these studies is limited (Figure S4A). Comparing the combination of three previously described enhancer maps with our dataset, 7,948 of the 32,353 active enhancers identified by ChIP-STARR-seq were among these predicted enhancers (n = 76,666; union of all datasets) (Table S2). Several putative enhancers predicted by these previous studies that were inactive by ChIP-STARR-seq were tested in luciferase assays but none possessed enhancer activity in this assay (Figure S4B). Enrichment analysis using GREAT (McLean et al., 2010) showed that the active ChIP-STARR-seq enhancer subset overlapping with previously predicted enhancers had stronger enrichment for gene ontology (GO) terms related to ESC biology than terms identified from all predicted enhancers (Table S3). This "core enhancer module" (Figure 4A) includes enhancers in close proximity to ESC TFs (*NANOG, OCT4)* and signaling pathway genes (TGF-β, FGF, WNT signaling). The remaining 24,405 enhancers with high ChIP-STARR-seq activity, that were not predicted previously, had GO terms associated with more generic processes; e.g., regulation of transcription, chromosome organization, housekeeping processes, and cytoskeleton organization. We therefore refer to these enhancers as the "extended enhancer module."

A comparison of the ChIP-seq signal intensity for all peaks to peaks associated with either the core or extended module indicates that enhancers of the extended module generally had slightly lower association with H3K4me1, NANOG, and OCT4 (Figure 4B). Reduced NANOG and OCT4 binding suggests that extended enhancers rely less on ESC-specific TFs, which is supported by a machine learning classifier attempt to discriminate enhancer modules based on sequence features (Figures S4D and S4E). This analysis demonstrated that core enhancers could be identified by CG dinucleotide frequency, GC content, and the occurrence of OCT4 and NANOG binding motifs. Nonetheless, the extended module sequences are *bona fide* enhancers, as their activities are similar to core enhancers (Figure 4C). Similarly, the expression of genes associated with the core and extended enhancer modules was comparable, with both gene sets expressed significantly above average ($p < 0.05$) (Figures 4D and S4C). Consistent with function in many cell types, expression of genes associated with the extended enhancer module was higher than core-module-associated genes in data from somatic tissues obtained from the RNA-Seq Atlas (Krupp et al., 2012) (Figure 4E) and GTEx Consortium (2013) (Figure S4F). To provide context, we included orthogonal "housekeeping" (Eisenberg and Levanon, 2013) and "tissue-specific" gene sets (Lachmann et al., 2018) in this analysis. Enrichment analysis using Enrichr (Chen et al., 2013) with data from ENCODE Project Consortium (2012) or ChEA (Lachmann et al., 2010) showed that core module enhancers were enriched near genes bound by NANOG, TCF3, SOX2, and OCT4, whereas extended enhancer module enhancers showed preferential enrichment of broadly expressed factors, such as TAF1, YY1, BRCA1, and ATF2 (Figure 4F; Table S3). Core enhancers were often found in regions associated with enhancer-like chromatin in H9 (Kundaje et al., 2015) (Figure 4G). In contrast, ~6% of extended module enhancers are annotated as heterochromatic or bivalent in H9 chromatin, suggesting that the activity of these enhancers may be suppressed by endogenous chromatin. The majority of enhancers from either the core or extended modules showed a similar distance distribution around TSSs, although a subset of extended module enhancers (n = 4,731) lie within 2 kb of TSSs (Figure 4H). GO terms

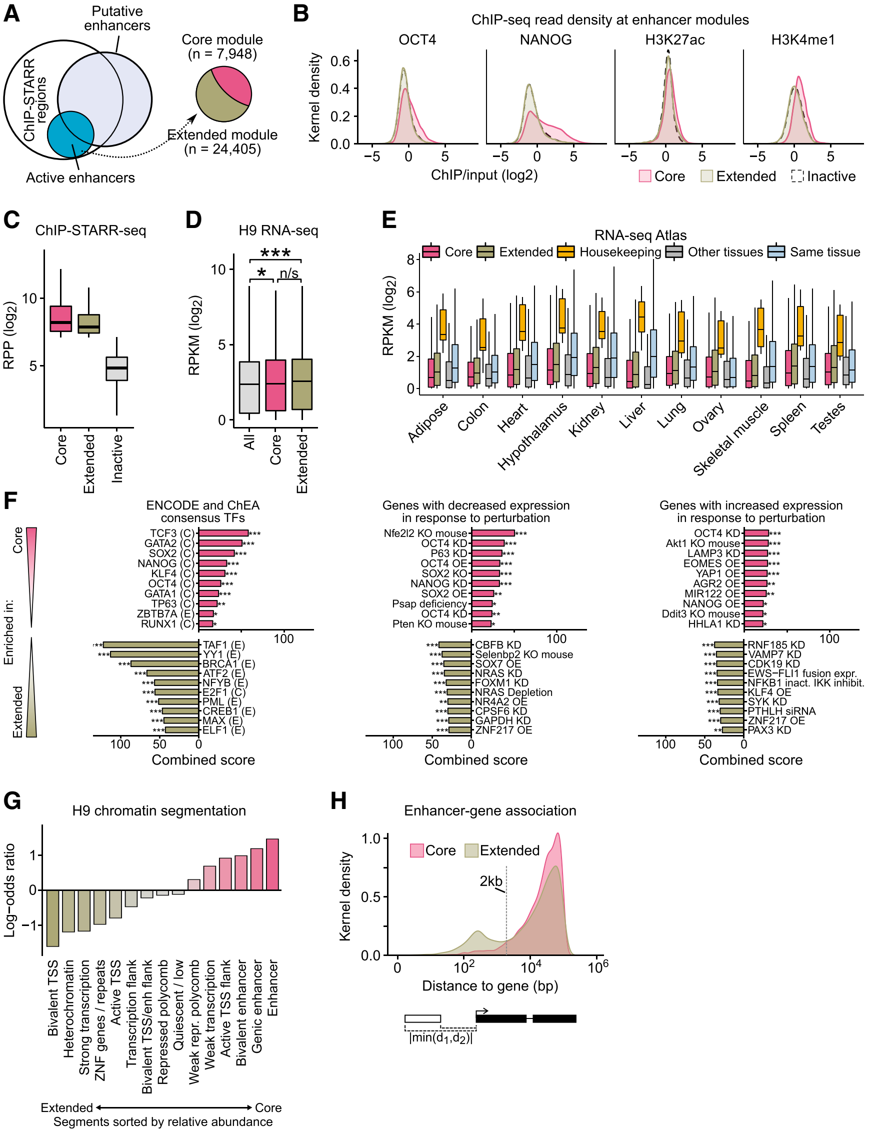

Figure 4. Active Enhancers Include Core and Extended ESC-Enhancer Modules

(A) The overlap between published putative enhancers (Hawkins et al., 2011; Rada-Iglesias et al., 2011; Xie et al., 2013) (light blue) and regions assessed by ChIP-STARR-seq (white) or called active (RPP $\geq$ 138; blue). We refer to ChIP-STARR-seq enhancers overlapping published putative enhancers as the "core module" and non-overlapping regions as the "extended module."

(B) Kernel density plots of the distribution of enrichment values in ESCs for the indicated factor for peaks associated with the core or extended modules or for inactive regions.

(C) RPP values for all assessed genomic regions compared to enhancers from the core or extended modules. Boxes are IQR; line is median; and whiskers extend to 1.5× the IQR.

(D) RNA-seq in H9 (Takashima et al., 2014) for all genes compared to genes associated with either core or extended enhancer modules. Boxes like in (C). RPKM, reads per kilobase million. * $p < 0.05$; *** $p < 0.001$ (t test).

(E) Gene expression in tissues from the RNA-seq Atlas (Krupp et al., 2012) for all genes linked to the core or extended modules. Housekeeping (Eisenberg and Levanon, 2013) and tissue-specific genes (Lachmann et al., 2018) are also shown. Tissue-specific genes are split into the one indicated (same; x axis) or "other tissues." As no tissue-specific gene set was available for hypothalamus, whole-brain-specific genes were used. Boxes like in D.

(F) Enrichment analysis (Enrichr) testing genes associated with the core (top) and extended (bottom) modules. Top-10 results for TF binding sites from ENCODE and ChEA (left) and genes downregulated (middle) or upregulated (right) upon single-gene perturbations from GEO.

(G) Relative enrichment (log-odds ratio in ESCs compared to all) of H9 chromatin segments (Kundaje et al., 2015) in core and extended module enhancers.

(H) Kernel density plot of the distance to associated genes for core and extended module enhancers. Shortest distance from either enhancer region boundary was recorded.

associated with the TSS-proximal subset are enriched for terms related to metabolic processes and housekeeping functions, whereas terms associated with TSS-distal enhancers include cell fate and differentiation annotations (Table S3). This indicates that a subset of extended module enhancers may be linked to housekeeping genes. ChIP-STARR-seq therefore identified by function, previously unappreciated enhancer sequences characterized by lower enrichment of enhancer-associated histone modifications and pluripotency-related TFs but with comparable enhancer activity.

Major Changes in Enhancer Activity upon Induction of Naive Pluripotency

To augment the catalog of functional enhancers in ESCs and to gauge the dynamics of enhancer activity we applied ChIP-STARR-seq to a closely related cell type. Primed H9 ESCs were converted to naive ESCs (Figures S5A–S5D). Characterization of established cultures agreed with prior studies (Barakat et al., 2015; Gafni et al., 2013), as did ChIP-qPCR and ChIP-seq for NANOG, OCT4, H3K4me1, and H3K27ac (Figures S5E–S5I). ChIP-STARR-seq plasmid libraries generated from naive ESCs (Figures 5A and S6) were transfected into naive ESCs and for comparison, into primed ESCs. Transfections followed by RNA-seq readout yielded measurements of enhancer activity in naive ESCs comparable to those obtained previously in primed ESCs, albeit at slightly lower reproducibility (mean correlation r = 0.63 at read count $\geq$ 5). Enhancer activity was categorized using the threshold applied previously (Table S2; Data S1). 359,880 regions covered by plasmids in naive ESCs (Figure S6C) were analyzed, identifying 36,417 enhancers. Again, only a fraction of ChIP-seq peaks displayed activity with peaks marked by OCT4, H3K27ac and H3K4me1 showing the highest proportion of activity (Figure S6D). LOLA enrichment analysis of TFs from CODEX for the naive enhancer class (Figure 5B;

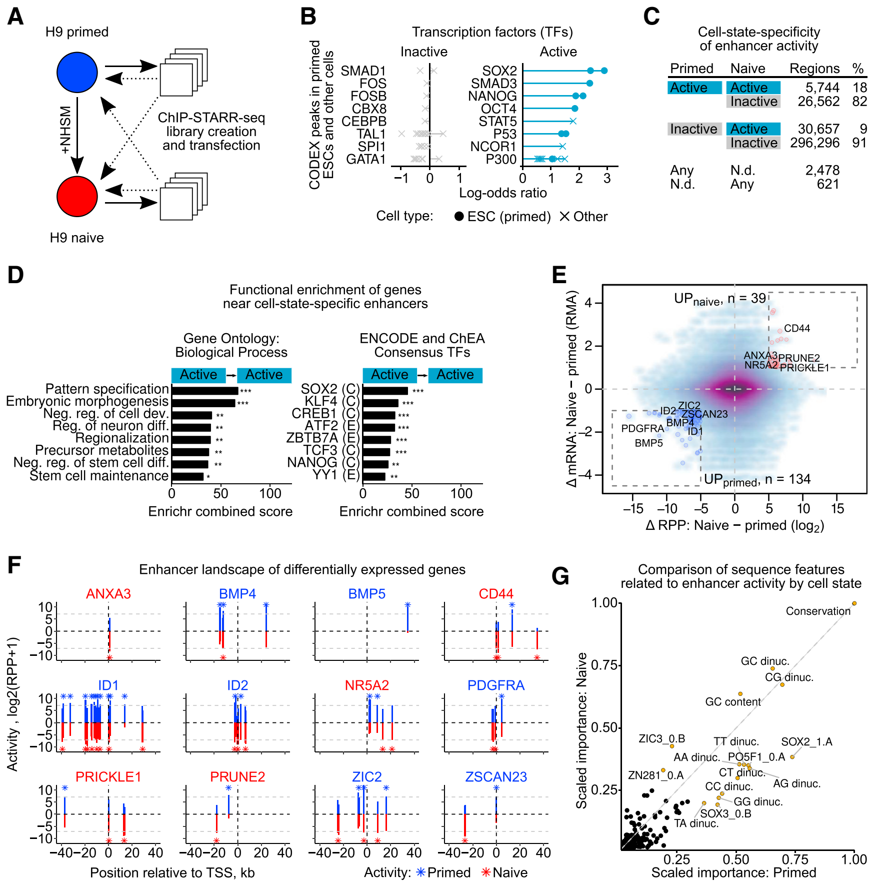

Figure 5. Changes in Enhancer Activity upon Induction of Naive Pluripotency

(A) Overview of primed to naive conversion and ChIP-STARR-seq cross-over design.

(B) Relative enrichment of TFs from CODEX (Sánchez-Castillo et al., 2015) in inactive, and active enhancers in naive hESCs. Plots like in Figure 2G.

(C) Table of relative changes in enhancer activity between primed and naive ESCs.

(D) Enrichment analysis (Enrichr) to test genes near enhancers active in both primed and naive ESCs against GO assignments (left) or binding sites from ENCODE and ChEA ChIP-seq (right).

(E) Scatterplot contrasting average changes in enhancer activity with changes in associated gene expression. Genes with strong concordant changes in enhancer activity and gene expression are shown using the thresholds: $|\max(\Delta RPP)| \geq 5$, $|\text{mean}(\Delta mRNA)| \geq 1$.

(F) Visualization of enhancer activity in ChIP-STARR-seq regions near selected genes (boxes in E; TSS ± 40 kb) with differential expression in primed and naive ESCs. Bars indicate enhancer activity (RPP) in primed (blue) and naive (red) ESCs. Grey dashed bars indicate activity threshold for active enhancers. Active enhancers are highlighted with asterisks. Gene name color shows the state expressing the gene the highest.

(G) Scatterplot of scaled variable importance of sequence features used to discriminate active and inactive regions in primed and naive ESCs. In both cases, a random forest classifier was trained.

Table S3), identified a similar TF profile as in primed ESCs (compare to Figure 2G). Sites bound by pluripotency-related TFs (e.g., SOX2 and NANOG) were also strongly represented at enhancers active in naive ESCs. Enrichment analysis of ENCODE ChIP-seq datasets (Figure S6E) and chromatin segmentations (Figures S6F and S6G) (Ernst et al., 2011; Kundaje et al., 2015) confirmed overlap with ESC TF binding sites.

Having extensive genome-wide enhancer maps for both pluripotent states allowed a global comparison of enhancer usage in both primed and naive ESCs (Figure 5C). Only 18% of enhancers active in primed ESCs maintained activity in naive ESCs (Active→Active), whereas 82% became inactive (Active→Inactive). Conversely, 9% of inactive regions in primed ESCs gained activity (Inactive→Active). Despite these extensive changes, the relative ranking of RPP values is stable, indicating that the highest and lowest activity score are comparable (Figure S6H). The changes in activity are not explicable by altered affinity of TF binding alone, as illustrated by discriminating peaks into strongly and weakly bound regions (Figure S6I) and applying the same analysis to ChIP-seq affinity values (Figure S6J). For instance, only 36% of regions that maintained strong enhancer activity in both states were also strongly bound in both states, whereas 15.3% of regions switched from strongly to weakly bound or vice versa. Enrichment analysis of enhancers maintaining or switching activity level (Figures 5D and S6K; Table S3) revealed that enhancers with high activity in both cell states (Active→Active) were related to suppression of differentiation processes and maintenance of stem cells, whereas genes near enhancers that lost activity (Active→Inactive) were annotated with generic expression-related terms. No significant GO terms were associated with enhancers that gained activity or regions that remained inactive, though this may be due to lack of annotation in naive ESCs. However, examining ChIP-seq data from ENCODE and ChEA indicated that enhancers that were active only in naive cells were enriched for transcriptional activators, such as ATF2, TAF1, or BRCA1, that occur near target promoters. Comparative analysis of core and extended module enhancers (see Figure 4), showed that core enhancers were significantly ($p < 2.2 \times 10^{-16}$) more likely to be active in naive ESCs than either extended module enhancers or enhancers inactive in primed ESCs (Figure S6L).

To relate changes in enhancer activity to differences in the expression of regulated genes, we plotted the average difference in enhancer RPP levels between naive and primed ESCs against the expression of nearby genes (Figure 5E). We highlighted genes with at least one strong enhancer change. Detailed examination of the ChIP-STARR-seq regions in the proximity (≤40 kb) of the TSS of these genes (Figure 5F; http://hesc-enhancers.computational-epigenetics.org) confirmed increased enhancer activities for several genes that were expressed higher in naive ESCs (e.g., CD44, ANXA3). In contrast, several genes were expressed more highly in primed ESCs and in each case

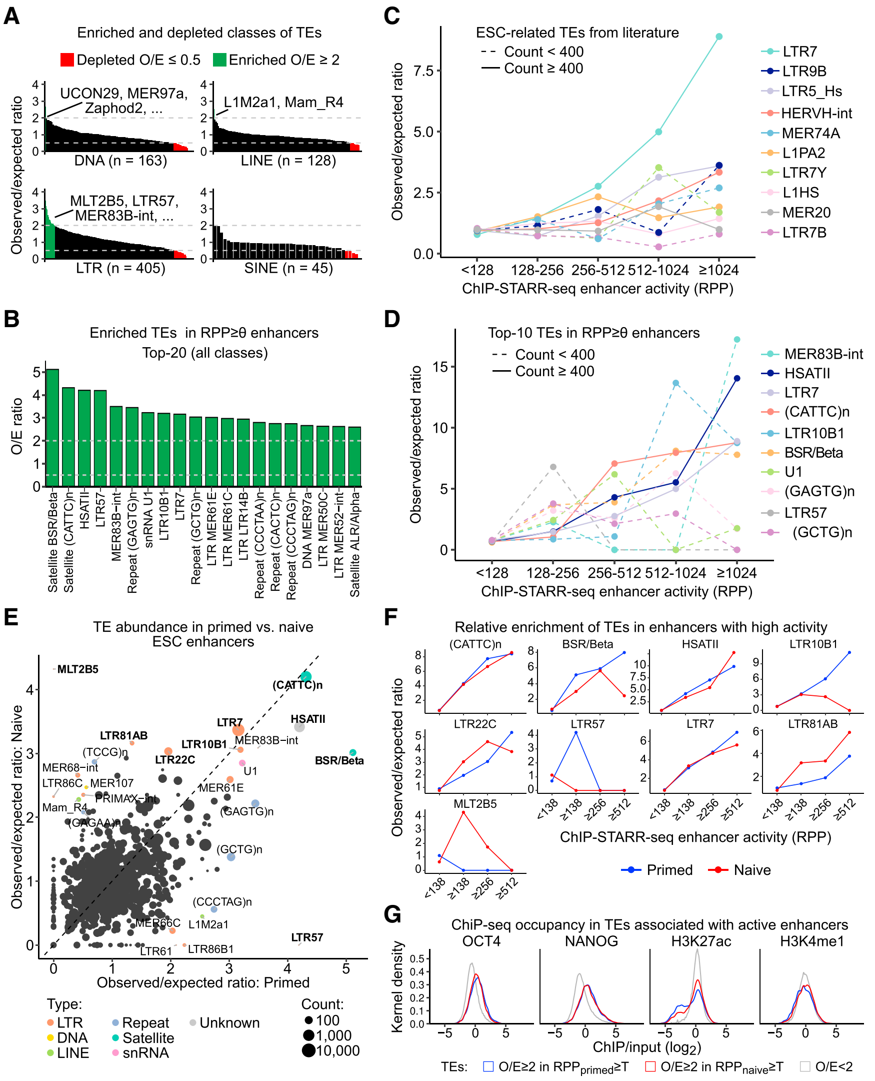

Figure 6. Distinct Transposable Elements Are Associated with Enhancers of Differing Activity in ESCs

(A) Enrichment ratios for the occurrence of TE families (LTR, DNA, SINE, and LINE) in high activity ChIP-STARR-seq enhancers (RPP $\geq$ 138).

(B) Top-25 most enriched TE families in active enhancers.

(C) Enrichment ratio versus activity level for distinct TE families.

(D) Like in (C), but for the top-10 most enriched families of TEs in (B).

(E) Comparison of the enrichment ratios in primed and naive ESCs. Each repeat element is shown by a dot with the size proportional to the number of overlaps with ChIP-STARR-seq regions. Elements with O/E $\geq$ 3 in naive or primed or with strong differences between both (O/E $\geq$ 2 and $\Delta\log_2$ (O/E) $\geq$ 2) are labeled.

(F) Relative enrichment of selected TEs (from E) in primed (blue) and naive (red) ESCs as a function of enhancer activity level (RPP).

(G) Kernel density plots of coverage (ChIP-seq/input) in ESCs for the indicated factor for all TEs overrepresented (O/E > 2) in active enhancers.

enhancers with increased activity in primed ESCs could be identified that may drive preferential expression in primed ESCs (e.g., BMP4/5, ID1/2). Notably, some genes showed concordant changes in multiple adjacent enhancers that presumably jointly drive expression changes (PRICKLE1, BMP4), whereas other genes switched activity from one enhancer to another (PRUNE2, CD44, and ZSCAN23). The catalog of functional enhancers presented here will help to decipher the complexity of enhancer/target interactions directing gene expression.

Next, we trained a classifier to discriminate active from inactive regions in naive ESCs and compared the results to those we obtained previously (Figure 5G and S6M; Figure 3). We find a consistent contribution of evolutionary conservation and GC/CG dinucleotide frequencies to enhancer activity. Notably, the relative importance of TF binding motifs shifts slightly between naive and primed: e.g., ZIC3 is linked to naive ESCs (Warrier et al., 2017), and SOX3 is linked to primed ESCs, in line with a recent report on primed pluripotent mouse cells (Corsinotti et al., 2017).

The Occurrence of Various Transposable Elements Is Associated with Enhancer Activity

As chromatin associated with repetitive DNA was found in active enhancers (Figure 2F), we examined the link between repeats and enhancer activity more closely. Large portions of mammalian genomes are derived from TEs which are linked to TF binding sites (Glinsky, 2015; Kunarso et al., 2010), but whether this enrichment reflects enhancer activity has not been determined genome-wide. To assess ChIP-STARR-seq enhancers for the occurrence of TE sequences, we used the RepeatMasker annotation (Kent et al., 2002). The number of TE-derived sequences in active and inactive regions was compared to the number detected in all genomic regions (Figure 6; Table S4). LTR-containing TEs, such as LTR57, were enriched in primed ESCs enhancers (Figure 6A). However, not all LTR-containing TEs were enriched at active enhancers. The most enriched elements were satellite repeats and LTR family members (Figure 6B). For TEs enriched for NANOG and OCT4 binding (e.g., LTR9B) (Kunarso et al., 2010) or TEs enriched at candidate human-specific regulatory loci (e.g., LTR7) (Glinsky, 2015), the observed enrichment increased further with increasing activity (Figures 6C and 6D). Indeed, LTR7, LTR9B and HERVH-int show the strongest enrichment at the highest activity enhancers. In contrast, other TE families previously linked to human-specific TF binding sites (Glinsky, 2015), were either not (L1HS) or only weakly (L1PA2) enriched at active enhancers. Although many repeat families were found equally in primed and naive ESCs (e.g., LTR7, (CATTC)n), other families showed less or no enrichment in one of the two states (e.g., LTR81AB, LTR57)

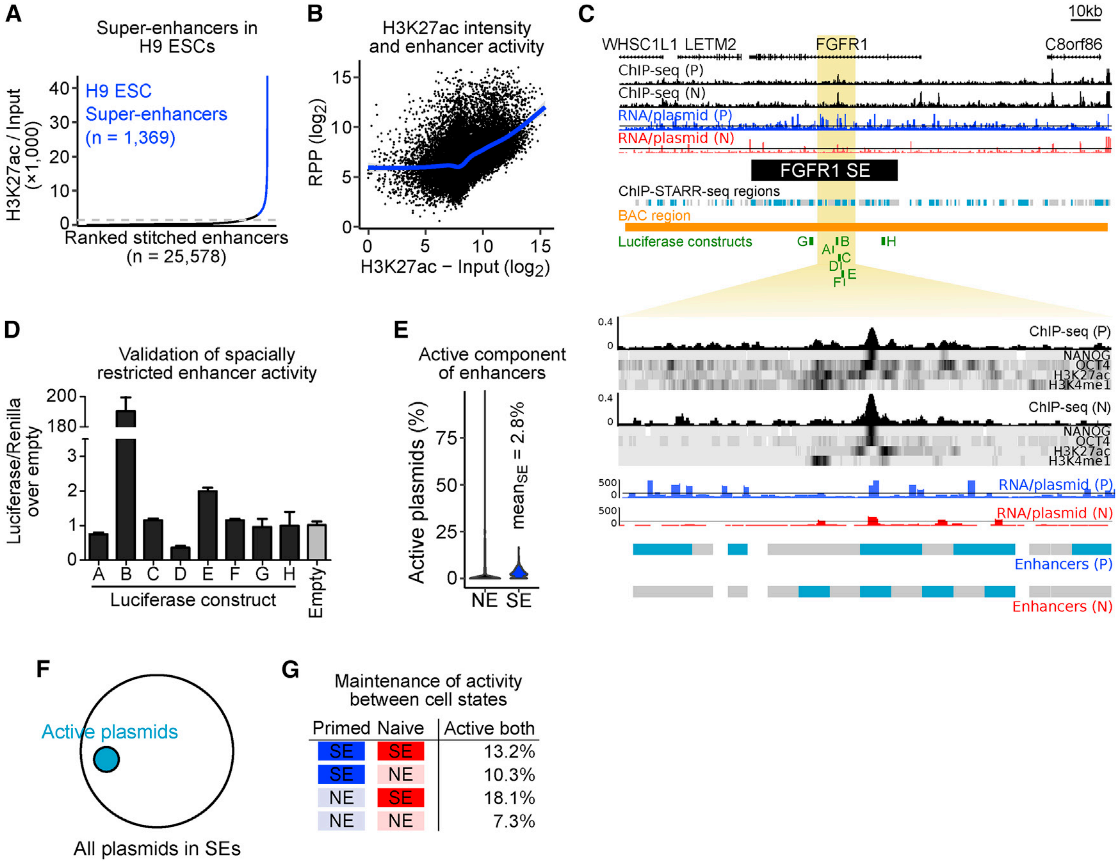

Primed	Naive	Active both
SE	SE	13.2%
SE	NE	10.3%
NE	SE	18.1%
NE	NE	7.3%

Figure 7. ChIP-STARR-Seq Dissects Super-Enhancers into Functional Elements

(A) SEs were called from H3K27ac ChIP-seq data using ROSE (Whyte et al., 2013).

(B) Scatterplot of SE intensity (H3K27ac enrichment over input) with ChIP-STARR-seq activity. *r*, Pearson correlation; blue line indicates a generalized additive model fit.

(C) SE overlapping *FGFR1*, with ChIP-seq tracks for the indicated factors in primed/naive ESCs. Top plot: SE locus; bottom plot: zooms into second intron. Shown are the positions of regions assessed by ChIP-STARR-seq (gray) and active enhancers (blue) from this study and coordinates of luciferase constructs matching selected enhancers (labeled A–H). Enhancer activities are concentrated at small regions.

(D) Luciferase assays of DNA sequences depicted in green in (C); n = 2. Error bars represent SD.

(E) Violin plots of the proportion of active plasmids (RPP $\geq$ 138) for 1,369 SEs compared to normal enhancers (NE).

(F) Sketch of the active subspace (covered by plasmids with RPP $\geq$ 138) of the entire SE space (all plasmids within SEs).

(G) Table of the percentage of ChIP-STARR-seq plasmids representing regions within SEs and NEs active in primed and naive ESCs (RPP $\geq$ 138). Groups of enhancers that were called SEs in both, in on, or in neither state are distinguished.

(Figures 6E and 6F). In general, TEs that were overrepresented in active enhancers showed increased binding of NANOG and OCT4, but not H3K27ac or H3K4me1 (Figure 6G). These results indicate that certain families of TEs are overrepresented at active enhancers and that their enrichment correlates with enhancer activity in a cell-state-dependent manner. However, not all TEs of the same type are associated with active enhancers, nor do all TEs enriched in pluripotency TF binding sites occupy active enhancers.

ChIP-STARR-Seq Dissects Super-Enhancers into Small Functional Units

Recently, large linear tracts of chromatin, referred to as "SEs" have been identified that function to regulate lineage-specific gene expression (Whyte et al., 2013). Compared to traditional enhancers, SEs have increased binding of Mediator, specific histone marks and lineage-specific TFs. Whether the full length of SEs is required for biological activity is a matter of debate (Hay and Hughes, 2016; Moorthy et al., 2017; Shin et al., 2016). We used our enhancer catalog to dissect the regulatory potential of DNA underlying SE regions. SEs were first identified by H3K27ac enrichment in primed (Figure 7A; Data S1) and naive (Figure S7A) ESCs. Alignment of ChIP-STARR-seq data to these SEs showed that the H3K27ac intensity used to define SEs correlated to RPP levels (Figures 7B, S7B, and S7C), supporting the notion that SE-likeness is an indicator of enhancer activity. SEs discovered here overlapped strongly between primed and naive ESCs (n = 824 SEs shared), containing many of the previously described H1 ESC SEs (Figures S7D and S7E) (Hnisz et al., 2013). Detailed examination of the FGFR1 SE indicated strong RPP signals originating from small regions within the SE (Figure 7C). To exclude the possibility that this observation was

due to limited coverage in our ChIP-STARR-seq libraries, we included additional STARR-seq libraries made from BACs covering the FGFR1 SE and two other SEs providing robust coverage of the entire SEs plus flanking regions (Figures 7C and S7F). Luciferase assays confirmed spatially restricted enhancer activity of DNA in the neighborhood of the central active region of the FGFR1 SE. Strong activity was confined to a 596 bp region with other DNA elements from this SE devoid of enhancer activity (Figure 7D). Homozygous deletion of this region by CRISPR-Cas9 significantly reduced expression of *FGFR1* and *WHSC1L1* compared to wild-type cells, without affecting expression of other flanking genes (Figures S7G and S7H). Homozygous deletion of two other parts of this SE did not affect gene expression of target and flanking genes. This indicates that the *FGFR1* SE is composed of small units with enhancer activity. To test whether this finding is valid globally, we examined the relative abundance of active plasmids (RPP $\geq$ 138) in SEs compared to "normal" enhancers (NEs). Most enhancers contained only a small percentage of active plasmids within their bounds (Figures 7E and 7F). Although this fraction was slightly higher in SEs than in NEs, it accounted for only a minority (2.8%) of the genome annotated as SEs. Therefore, only a small part of the large SEs has enhancer function (Figures 7F and S7I). Notably, regions within naive SEs or within SEs called in both primed and naive were more frequently active in both states (18.1% and 13.2%, respectively) than regions within primed SEs or outside SEs (Figure 7G). Since only a subspace of SEs displayed enhancer activity, we investigated the relationship between active components and H3K27ac ChIP-seq peaks by repeating the SE calling without stitching disjoint peaks (ROSE stitching distance = 0). However, the fraction of active plasmids remained unaffected indicating that H3K27ac occupancy alone cannot identify active SE components (Figure S7J).

DISCUSSION

In this study, we present a large-scale analysis of ESC enhancer activities. By using ChIP-STARR-seq we assessed the ability of sequences bound by OCT4, NANOG, or marked by H3K4me1 and H3K27ac to function as enhancers. Our results show that only a subset of these sequences displayed enhancer activity. We find that TF binding is linked with enhancer activity, in line with recent reports (Kwasnieski et al., 2014; Ernst et al., 2016; Kheradpour et al., 2013), but that no individual TF, histone mark or combination thereof could unequivocally predict enhancer activity. Our study identified a previously unrecognized group of functional enhancers that are active in ESCs but are associated with generic cell processes. This extended enhancer module is characterized by reduced binding of pluripotency-associated TFs and histone marks. This reduced binding might have placed these regions below the detection threshold in previous ChIP-seq-based studies that lacked a functional readout.

The use of an episomal-plasmid-based reporter system may be considered a limitation, as it does not fully recapitulate endogenous chromatin context (Inoue et al., 2017). It is also possible that in some cases cloned fragments might be too short to enable all the TF interactions that mediate enhancer function at the endogenous locus. However, the generally accepted definition of an enhancer focuses on the functional capacity of DNA to enhance transcription of a reporter gene in an orientation and position-independent manner (Banerji et al., 1981). Indeed, several lines of evidence argue for the broad usefulness of ChIP-STARR-seq as a high-throughput assay of enhancer function: (1) ChIP-STARR-seq confirmed the function of known enhancers; (2) genes near active enhancers tend to be more highly expressed; (3) active enhancers are marked by motifs of TF associated with ESCs; (4) active enhancers are enriched in genome annotations as enhancer chromatin; and (5) deletion of active enhancers from endogenous loci decreases expression of linked target genes, whereas deletion of sequences devoid of enhancer activity in ChIP-STARR-seq did not affect gene expression.

Previous studies identified crucial roles for OCT4, NANOG, and SMAD3, the latter of which are downstream mediators of TGF-β signaling in the maintenance of ESC pluripotency (James et al., 2005; Xu et al., 2008). Enhancer activity is enriched near these binding peaks, suggesting that these TFs may act combinatorially to provide enhancer function. Other studies have shown that heterotypic clusters of different TF binding sites can increase enhancer activity (Smith et al., 2013) and that sequences marked by H3K122ac but lacking H3K27ac can act as transcriptional enhancers (Pradeepa et al., 2016). It would be of future interest to decipher the individual contributions of TFs to these active enhancers. Several classes of TEs were also enriched at active enhancers, as reported recently (Ernst et al., 2016). TEs are enriched in species-specific TF binding sites and have been hypothesized to shape the enhancer network in ESCs (Glinsky, 2015; Kunarso et al., 2010). Our data indicate that only a limited number of TEs contribute to enhancer function and can do so in a cell-state-dependent manner.

Most enhancers studied to date lie within distal elements or intronic sequences. However, some sequences detected by ChIP-STARR-seq lie near TSSs (n = 3,283 active enhancers within 500bp of a TSS). As tested enhancers are inserted downstream of the GFP ORF in STARR-seq (Figure 1A) GFP-positive transcripts cannot be made by initiating transcription *in situ* from an inserted TSS. Therefore, sequences near a TSS can exert enhancer activity, in line with recent reports (Dao et al., 2017; Engreitz et al., 2016). Furthermore, a subset of extended module enhancers lies close (±2 kb) to a TSS and display GO enrichments related to housekeeping genes and metabolic processes. This suggests that nearby enhancers may regulate some human housekeeping genes. It would be interesting to investigate links between enhancers and promoters that distinguish housekeeping genes from developmental genes, as identified in *Drosophila* (Zabidi et al., 2015).

Several groups have recently developed culture conditions supporting a more naive ESC state enabling contribution to interspecies chimeras (Gafni et al., 2013; Takashima et al., 2014; Theunissen et al., 2014; Wu et al., 2017). Here, we have used one such culture condition to compare primed and naive ESCs and find that enhancer activity is altered substantially. Pluripotency in both states is established by differential use of regulatory elements that is partly reflected in gene expression changes. Further studies should clarify differences between states of pluripotency and how these relate to altered enhancer usage.

SEs are characterized by large domains marked by H3K27ac with increased binding of Mediator and other TFs. ChIP-STARR-seq analysis indicates that the majority of sequences within SEs lack enhancer activity. Rather, enhancer activity is limited to small domains within the SEs that frequently overlap with TF binding sites. This suggests that the observed chromatin signatures at SEs might be a consequence of enhancer activity from much smaller units. Recent reports suggest that SE constituents may function alternatively as independent and additive enhancers (Hay and Hughes, 2016; Moorthy et al., 2017), as constituents in a temporal and functional enhancer hierarchy (Shin et al., 2016), or as interdependent units (Hnisz et al., 2015) exhibiting synergy (Suzuki et al., 2017). The large-scale identification of such active constituents within SEs reported here should help to decipher the regulatory mechanisms contributing to SE formation and function.

The catalog of functional enhancers presented here provides the means to refine models of the regulatory circuitry of ESCs and a framework for understanding transcriptional regulation in humans. Given the increasing appreciation of the importance of the regulatory genome in health and disease, we expect that this resource and the more widespread use of MPRAs, such as ChIP-STARR-seq, should advance basic and translational research.

STAR★METHODS

Detailed methods are provided in the online version of this paper and include the following:

- KEY RESOURCES TABLE
- CONTACT FOR REAGENT AND RESOURCE SHARING
- EXPERIMENTAL MODEL AND SUBJECT DETAILS
    - Cell lines
    - Cell Culture conditions
- METHOD DETAILS
    - Experimental Design
    - Chromatin immunoprecipitation
    - ChIP-qPCR
    - ChIP-seq, ChIP-STARR-seq plasmid library preparation
    - Transfection of plasmid libraries
    - ChIP-STARR-seq RNA and DNA samples
    - RT-qPCR
    - Immunostaining
    - Western blotting
    - Luciferase assays
    - Alternative promoter STARR-seq constructs
    - CRISPR/Cas9 genome editing
- QUANTIFICATION AND STATISTICAL ANALYSIS
    - ChIP-seq and ChIP-STARR-seq data processing
    - ChIP-STARR-seq enhancer activity levels
    - Motif enrichment analysis for ChIP-seq data
    - Assignment of enhancers to genes
    - Comparison to previously published enhancers
    - Functional enrichment analysis
    - Machine learning
    - Enrichment analysis for transposable elements
    - Super-enhancer analysis
    - Statistics for qPCR and luciferase assays
- DATA AND SOFTWARE AVAILABILITY
    - Data availability
- ADDITIONAL RESOURCES

SUPPLEMENTAL INFORMATION

Supplemental Information includes seven figures, five tables, and one data file and can be found with this article online at https://doi.org/10.1016/j.stem.2018.06.014.

ACKNOWLEDGMENTS

We thank S. Pollard, D. O'Carroll, A. Soufi, and S. Tomlinson for comments; E. Hall-Ponsele, F. Rossi, and G. Schaaf for technical support; and R. Pantier, J. Zhang, and other colleagues for discussions. We thank A. Stark for the STARR-seq plasmid; F. Zhang for eSpCas9(1.1); and D. Hay for H9 ESCs. I.C.'s lab is supported by the Medical Research Council (UK) and The Wellcome Trust. T.S.B. was supported by fellowships from Niels Stensen, EMBO (EMBO-ALTF 1272-2014), and Marie Sklodowska-Curie (H2020 MSCA-IF). F.H. was supported by a DFG fellowship (HA 7723/1-1). C.B. is supported by a New Frontiers Group Award of the Austrian Academy of Sciences and an ERC Starting Grant (679146).

AUTHOR CONTRIBUTIONS

T.S.B. and I.C. conceived the study. T.S.B. performed the molecular biology and contributed to the bioinformatics analysis and cell culture. A.F.R. developed the primary data analysis pipeline. F.H. performed bioinformatics analysis, visualization, and interpretation. M.Z. performed cell culture, performed immunofluorescence, and generated CRISPR/Cas9-deleted clones, together with E.P. C.B. supervised the bioinformatics analysis. T.S.B., F.H., and I.C. wrote the paper, with input from all authors.

DECLARATION OF INTERESTS

The authors declare no competing interests.

Received: June 6, 2017
Revised: March 2, 2018
Accepted: June 19, 2018
Published: July 19, 2018

REFERENCES

Afgan, E., Baker, D., van den Beek, M., Blankenberg, D., Bouvier, D., Čech, M., Chilton, J., Clements, D., Coraor, N., Eberhard, C., et al. (2016). The Galaxy platform for accessible, reproducible and collaborative biomedical analyses: 2016 update. Nucleic Acids Res. *44* (W1), W3–W10.

Arnold, C.D., Gerlach, D., Stelzer, C., Boryń, L.M., Rath, M., and Stark, A. (2013). Genome-wide quantitative enhancer activity maps identified by STARR-seq. Science *339*, 1074–1077.

Bailey, T.L., and Machanick, P. (2012). Inferring direct DNA binding from ChIP-seq. Nucleic Acids Res. *40*, e128.

Banerji, J., Rusconi, S., and Schaffner, W. (1981). Expression of a beta-globin gene is enhanced by remote SV40 DNA sequences. Cell *27*, 299–308.

Barakat, T.S., Ghazvini, M., de Hoon, B., Li, T., Eussen, B., Douben, H., van der Linden, R., van der Stap, N., Boter, M., Laven, J.S., et al. (2015). Stable X chromosome reactivation in female human induced pluripotent stem cells. Stem Cell Reports *4*, 199–208.

Buecker, C., Chen, H.H., Polo, J.M., Daheron, L., Bu, L., Barakat, T.S., Okwieka, P., Porter, A., Gribnau, J., Hochedlinger, K., and Geijsen, N. (2010). A murine ESC-like state facilitates transgenesis and homologous recombination in human pluripotent stem cells. Cell Stem Cell *6*, 535–546.

Buenrostro, J.D., Giresi, P.G., Zaba, L.C., Chang, H.Y., and Greenleaf, W.J. (2013). Transposition of native chromatin for fast and sensitive epigenomic profiling of open chromatin, DNA-binding proteins and nucleosome position. Nat. Methods *10*, 1213–1218.

Chen, E.Y., Tan, C.M., Kou, Y., Duan, Q., Wang, Z., Meirelles, G.V., Clark, N.R., and Ma'ayan, A. (2013). Enrichr: interactive and collaborative HTML5 gene list enrichment analysis tool. BMC Bioinformatics *14*, 128.

Corsinotti, A., Wong, F.C., Tatar, T., Szczerbinska, I., Halbritter, F., Colby, D., Gogolok, S., Pantier, R., Liggat, K., Mirfazeli, E.S., et al. (2017). Distinct SoxB1 networks are required for naïve and primed pluripotency. eLife *6*, e27746.

Dao, L.T.M., Galindo-Albarrán, A.O., Castro-Mondragon, J.A., Andrieu-Soler, C., Medina-Rivera, A., Souaid, C., Charbonnier, G., Griffon, A., Vanhille, L., Stephen, T., et al. (2017). Genome-wide characterization of mammalian promoters with distal enhancer functions. Nat. Genet. *49*, 1073–1081.

Eisenberg, E., and Levanon, E.Y. (2013). Human housekeeping genes, revisited. Trends Genet. *29*, 569–574.

ENCODE Project Consortium (2012). An integrated encyclopedia of DNA elements in the human genome. Nature *489*, 57–74.

Engreitz, J.M., Haines, J.E., Perez, E.M., Munson, G., Chen, J., Kane, M., McDonel, P.E., Guttman, M., and Lander, E.S. (2016). Local regulation of gene expression by lncRNA promoters, transcription and splicing. Nature *539*, 452–455.

Ernst, J., Kheradpour, P., Mikkelsen, T.S., Shoresh, N., Ward, L.D., Epstein, C.B., Zhang, X., Wang, L., Issner, R., Coyne, M., et al. (2011). Mapping and analysis of chromatin state dynamics in nine human cell types. Nature *473*, 43–49.

Ernst, J., Melnikov, A., Zhang, X., Wang, L., Rogov, P., and Mikkelsen, T.S. (2016). Genome-scale high-resolution mapping of activating and repressive nucleotides in regulatory regions. Nat Biotechnol. *34*, 1180–1190.

Gafni, O., Weinberger, L., Mansour, A.A., Manor, Y.S., Chomsky, E., Ben-Yosef, D., Kalma, Y., Viukov, S., Maza, I., Zviran, A., et al. (2013). Derivation of novel human ground state naive pluripotent stem cells. Nature *504*, 282–286.

Gifford, C.A., Ziller, M.J., Gu, H., Trapnell, C., Donaghey, J., Tsankov, A., Shalek, A.K., Kelley, D.R., Shishkin, A.A., Issner, R., et al. (2013). Transcriptional and epigenetic dynamics during specification of human embryonic stem cells. Cell *153*, 1149–1163.

Glinsky, G.V. (2015). Transposable elements and DNA methylation create in embryonic stem cells human-specific regulatory sequences associated with distal enhancers and noncoding RNAs. Genome Biol. Evol. *7*, 1432–1454.

Grant, C.E., Bailey, T.L., and Noble, W.S. (2011). FIMO: scanning for occurrences of a given motif. Bioinformatics *27*, 1017–1018.

GTEx Consortium (2013). The genotype-tissue expression (GTEx) project. Nat. Genet. *45*, 580–585.

Hanna, J., Cheng, A.W., Saha, K., Kim, J., Lengner, C.J., Soldner, F., Cassady, J.P., Muffat, J., Carey, B.W., and Jaenisch, R. (2010). Human embryonic stem cells with biological and epigenetic characteristics similar to those of mouse ESCs. Proc. Natl. Acad. Sci. USA *107*, 9222–9227.

Hawkins, R.D., Hon, G.C., Yang, C., Antosiewicz-Bourget, J.E., Lee, L.K., Ngo, Q.M., Klugman, S., Ching, K.A., Edsall, L.E., Ye, Z., et al. (2011). Dynamic chromatin states in human ES cells reveal potential regulatory sequences and genes involved in pluripotency. Cell Res. *21*, 1393–1409.

Hay, D., and Hughes, J.R. (2016). Genetic dissection of the alpha-globin super-enhancer in vivo. Nat Genet. *48*, 895–903.

Hnisz, D., Abraham, B.J., Lee, T.I., Lau, A., Saint-André, V., Sigova, A.A., Hoke, H.A., and Young, R.A. (2013). Super-enhancers in the control of cell identity and disease. Cell *155*, 934–947.

Hnisz, D., Schuijers, J., Lin, C.Y., Weintraub, A.S., Abraham, B.J., Lee, T.I., Bradner, J.E., and Young, R.A. (2015). Convergence of developmental and oncogenic signaling pathways at transcriptional super-enhancers. Mol. Cell *58*, 362–370.

Hoffman, M.M., Ernst, J., Wilder, S.P., Kundaje, A., Harris, R.S., Libbrecht, M., Giardine, B., Ellenbogen, P.M., Bilmes, J.A., Birney, E., et al. (2013). Integrative annotation of chromatin elements from ENCODE data. Nucleic Acids Res. *41*, 827–841.

Inoue, F., Kircher, M., Martin, B., Cooper, G.M., Witten, D.M., McManus, M.T., Ahituv, N., and Shendure, J. (2017). A systematic comparison reveals substantial differences in chromosomal versus episomal encoding of enhancer activity. Genome Res. *27*, 38–52.

James, D., Levine, A.J., Besser, D., and Hemmati-Brivanlou, A. (2005). TGFbeta/activin/nodal signaling is necessary for the maintenance of pluripotency in human embryonic stem cells. Development *132*, 1273–1282.

Ji, X., Dadon, D.B., Powell, B.E., Fan, Z.P., Borges-Rivera, D., Shachar, S., Weintraub, A.S., Hnisz, D., Pegoraro, G., Lee, T.I., et al. (2016). 3D chromosome regulatory landscape of human pluripotent cells. Cell Stem Cell *18*, 262–275.

Jiang, H., Lei, R., Ding, S.W., and Zhu, S. (2014). Skewer: a fast and accurate adapter trimmer for next-generation sequencing paired-end reads. BMC Bioinformatics *15*, 182.

Juven-Gershon, T., Cheng, S., and Kadonaga, J.T. (2006). Rational design of a super core promoter that enhances gene expression. Nat. Methods *3*, 917–922.

Kent, W.J., Sugnet, C.W., Furey, T.S., Roskin, K.M., Pringle, T.H., Zahler, A.M., and Haussler, D. (2002). The human genome browser at UCSC. Genome Res. *12*, 996–1006.

Kheradpour, P., Ernst, J., Melnikov, A., Rogov, P., Wang, L., Zhang, X., Alston, J., Mikkelsen, T.S., and Kellis, M. (2013). Systematic dissection of regulatory motifs in 2000 predicted human enhancers using a massively parallel reporter assay. Genome Res. *23*, 800–811.

Killick, R.E., and IA. (2014). changepoint: An R package for changepoint analysis. J. Stat. Softw *58*, 1–19.

Kreimer, A., Zeng, H., Edwards, M.D., Guo, Y., Tian, K., Shin, S., Welch, R., Wainberg, M., Mohan, R., Sinnott-Armstrong, N.A., et al. (2017). Predicting gene expression in massively parallel reporter assays: A comparative study. Hum. Mutat. *38*, 1240–1250.

Krupp, M., Marquardt, J.U., Sahin, U., Galle, P.R., Castle, J., and Teufel, A. (2012). RNA-Seq Atlas–a reference database for gene expression profiling in normal tissue by next-generation sequencing. Bioinformatics *28*, 1184–1185.

Kulakovskiy, I.V., Vorontsov, I.E., Yevshin, I.S., Soboleva, A.V., Kasianov, A.S., Ashoor, H., Ba-Alawi, W., Bajic, V.B., Medvedeva, Y.A., Kolpakov, F.A., and Makeev, V.J. (2016). HOCOMOCO: expansion and enhancement of the collection of transcription factor binding sites models. Nucleic Acids Res. *44* (D1), D116–D125.

Kunarso, G., Chia, N.Y., Jeyakani, J., Hwang, C., Lu, X., Chan, Y.S., Ng, H.H., and Bourque, G. (2010). Transposable elements have rewired the core regulatory network of human embryonic stem cells. Nat. Genet. *42*, 631–634.

Kundaje, A., Meuleman, W., Ernst, J., Bilenky, M., Yen, A., Heravi-Moussavi, A., Kheradpour, P., Zhang, Z., Wang, J., Ziller, M.J., et al.; Roadmap Epigenomics Consortium (2015). Integrative analysis of 111 reference human epigenomes. Nature *518*, 317–330.

Kwasnieski, J.C., Mogno, I., Myers, C.A., Corbo, J.C., and Cohen, B.A. (2012). Complex effects of nucleotide variants in a mammalian *cis*-regulatory element. Proc. Natl. Acad. Sci. USA *109*, 19498–19503.

Kwasnieski, J.C., Fiore, C., Chaudhari, H.G., and Cohen, B.A. (2014). High-throughput functional testing of ENCODE segmentation predictions. Genome Res. *24*, 1595–1602.

Lachmann, A., Xu, H., Krishnan, J., Berger, S.I., Mazloom, A.R., and Ma'ayan, A. (2010). ChEA: transcription factor regulation inferred from integrating genome-wide ChIP-X experiments. Bioinformatics *26*, 2438–2444.

Lachmann, A., Torre, D., Keenan, A.B., Jagodnik, K.M., Lee, H.J., Silverstein, M.C., Wang, L., and Ma'ayan, A. (2018). Massive mining of publicly available RNA-seq data from human and mouse. Nat. Commun. *9*, article number 1366.

Langmead, B., and Salzberg, S.L. (2012). Fast gapped-read alignment with Bowtie 2. Nat. Methods *9*, 357–359.

Lawrence, M., Huber, W., Pagès, H., Aboyoun, P., Carlson, M., Gentleman, R., Morgan, M.T., and Carey, V.J. (2013). Software for computing and annotating genomic ranges. PLoS Comput. Biol. *9*, e1003118.

Love, M.I., Huber, W., and Anders, S. (2014). Moderated estimation of fold change and dispersion for RNA-seq data with DESeq2. Genome Biol. *15*, 550.

McLean, C.Y., Bristor, D., Hiller, M., Clarke, S.L., Schaar, B.T., Lowe, C.B., Wenger, A.M., and Bejerano, G. (2010). GREAT improves functional interpretation of *cis*-regulatory regions. Nat. Biotechnol. *28*, 495–501.

Melnikov, A., Murugan, A., Zhang, X., Tesileanu, T., Wang, L., Rogov, P., Feizi, S., Gnirke, A., Callan, C.G., Jr., Kinney, J.B., et al. (2012). Systematic dissection and optimization of inducible enhancers in human cells using a massively parallel reporter assay. Nat. Biotechnol. *30*, 271–277.

Moorthy, S.D., Davidson, S., Shchuka, V.M., Singh, G., Malek-Gilani, N., Langroudi, L., Martchenko, A., So, V., Macpherson, N.N., and Mitchell, J.A. (2017). Enhancers and super-enhancers have an equivalent regulatory role in embryonic stem cells through regulation of single or multiple genes. Genome Res. *27*, 246–258.

Muerdter, F., Boryń, L.M., Woodfin, A.R., Neumayr, C., Rath, M., Zabidi, M.A., Pagani, M., Haberle, V., Kazmar, T., Catarino, R.R., et al. (2018). Resolving systematic errors in widely used enhancer activity assays in human cells. Nat. Methods *15*, 141–149.

Murtha, M., Tokcaer-Keskin, Z., Tang, Z., Strino, F., Chen, X., Wang, Y., Xi, X., Basilico, C., Brown, S., Bonneau, R., et al. (2014). FIREWACh: high-throughput functional detection of transcriptional regulatory modules in mammalian cells. Nat. Methods *11*, 559–565.

Patwardhan, R.P., Hiatt, J.B., Witten, D.M., Kim, M.J., Smith, R.P., May, D., Lee, C., Andrie, J.M., Lee, S.I., Cooper, G.M., et al. (2012). Massively parallel functional dissection of mammalian enhancers in vivo. Nat. Biotechnol. *30*, 265–270.

Pradeepa, M.M., Grimes, G.R., Kumar, Y., Olley, G., Taylor, G.C., Schneider, R., and Bickmore, W.A. (2016). Histone H3 globular domain acetylation identifies a new class of enhancers. Nat. Genet. *48*, 681–686.

Quinlan, A.R., and Hall, I.M. (2010). BEDTools: a flexible suite of utilities for comparing genomic features. Bioinformatics *26*, 841–842.

R. Development Core Team. (2008). R: A language and environment for statistical computing. R Foundation for Statistical Computing, http://www.R-project.org.

Rada-Iglesias, A., Bajpai, R., Swigut, T., Brugmann, S.A., Flynn, R.A., and Wysocka, J. (2011). A unique chromatin signature uncovers early developmental enhancers in humans. Nature *470*, 279–283.

Sánchez-Castillo, M., Ruau, D., Wilkinson, A.C., and Ng, F.S. (2015). CODEX: a next-generation sequencing experiment database for the haematopoietic and embryonic stem cell communities. Nucleic Acids Res *43*, *(Database issue)*, D1117–D1123.

Sheffield, N.C., and Bock, C. (2016). LOLA: enrichment analysis for genomic region sets and regulatory elements in R and Bioconductor. Bioinformatics *32*, 587–589.

Shin, H.Y., Willi, M., HyunYoo, K., Zeng, X., Wang, C., Metser, G., and Hennighausen, L. (2016). Hierarchy within the mammary STAT5-driven Wap super-enhancer. Nat. Genet. *48*, 904–911.

Slaymaker, I.M., Gao, L., Zetsche, B., Scott, D.A., Yan, W.X., and Zhang, F. (2016). Rationally engineered Cas9 nucleases with improved specificity. Science *351*, 84–88.

Smith, R.P., Taher, L., Patwardhan, R.P., Kim, M.J., Inoue, F., Shendure, J., Ovcharenko, I., and Ahituv, N. (2013). Massively parallel decoding of mammalian regulatory sequences supports a flexible organizational model. Nat. Genet. *45*, 1021–1028.

Sperber, H., Mathieu, J., Wang, Y., Ferreccio, A., and Hesson, J. (2015). The metabolome regulates the epigenetic landscape during naive-to-primed human embryonic stem cell transition. Nat. Cell Biol. *17*, 1523–1535.

Spitz, F., and Furlong, E.E. (2012). Transcription factors: from enhancer binding to developmental control. Nat. Rev. Genet. *13*, 613–626.

Suzuki, H.I., Young, R.A., and Sharp, P.A. (2017). Super-enhancer-mediated RNA processing revealed by integrative microrna network analysis. Cell *168*, 1000–1014.e15.

Takashima, Y., Guo, G., Loos, R., Nichols, J., Ficz, G., Krueger, F., Oxley, D., Santos, F., Clarke, J., Mansfield, W., et al. (2014). Resetting transcription factor control circuitry toward ground-state pluripotency in human. Cell *158*, 1254–1269.

Tewhey, R., Kotliar, D., Park, D.S., Liu, B., Winnicki, S., Reilly, S.K., Andersen, K.G., Mikkelsen, T.S., Lander, E.S., Schaffner, S.F., and Sabeti, P.C. (2016). Direct identification of hundreds of expression-modulating variants using a multiplexed reporter assay. Cell *165*, 1519–1529.

Theunissen, T.W., Powell, B.E., Wang, H., Mitalipova, M., Faddah, D.A., Reddy, J., Fan, Z.P., Maetzel, D., Ganz, K., Shi, L., et al. (2014). Systematic identification of culture conditions for induction and maintenance of naive human pluripotency. Cell Stem Cell *15*, 471–487.

Vanhille, L., Griffon, A., Maqbool, M.A., Zacarias-Cabeza, J., Dao, L.T., Fernandez, N., Ballester, B., Andrau, J.C., and Spicuglia, S. (2015). High-throughput and quantitative assessment of enhancer activity in mammals by CapStarr-seq. Nat. Commun. *6*, 6905.

Verfaillie, A., Svetlichnyy, D., Imrichova, H., Davie, K., Fiers, M., Kalender Atak, Z., Hulselmans, G., Christiaens, V., and Aerts, S. (2016). Multiplex enhancer-reporter assays uncover unsophisticated TP53 enhancer logic. Genome Res. *26*, 882–895.

Vockley, C.M., D'Ippolito, A.M., McDowell, I.C., Majoros, W.H., Safi, A., Song, L., Crawford, G.E., and Reddy, T.E. (2016). Direct GR binding sites potentiate clusters of TF binding across the human genome. Cell *166*, 1269–1281.e19.

Warrier, S., Van der Jeught, M., Duggal, G., Tilleman, L., Sutherland, E., Taelman, J., Popovic, M., Lierman, S., Chuva De Sousa Lopes, S., Van Soom, A., et al. (2017). Direct comparison of distinct naive pluripotent states in human embryonic stem cells. Nat. Commun. *8*, 15055.

Whyte, W.A., Orlando, D.A., Hnisz, D., Abraham, B.J., Lin, C.Y., Kagey, M.H., Rahl, P.B., Lee, T.I., and Young, R.A. (2013). Master transcription factors and mediator establish super-enhancers at key cell identity genes. Cell *153*, 307–319.

Wu, J., Platero-Luengo, A., Sakurai, M., Sugawara, A., Gil, M.A., Yamauchi, T., Suzuki, K., Bogliotti, Y.S., Cuello, C., Morales Valencia, M., et al. (2017). Interspecies chimerism with mammalian pluripotent stem cells. Cell *168*, 473–486.e15.

Xie, W., Schultz, M.D., Lister, R., Hou, Z., Rajagopal, N., Ray, P., Whitaker, J.W., Tian, S., Hawkins, R.D., Leung, D., et al. (2013). Epigenomic analysis of multilineage differentiation of human embryonic stem cells. Cell *153*, 1134–1148.

Xu, R.H., Sampsell-Barron, T.L., Gu, F., Root, S., Peck, R.M., Pan, G., Yu, J., Antosiewicz-Bourget, J., Tian, S., Stewart, R., and Thomson, J.A. (2008). NANOG is a direct target of TGFbeta/activin-mediated SMAD signaling in human ESCs. Cell Stem Cell *3*, 196–206.

Zabidi, M.A., Arnold, C.D., Schernhuber, K., Pagani, M., Rath, M., Frank, O., and Stark, A. (2015). Enhancer-core-promoter specificity separates developmental and housekeeping gene regulation. Nature *518*, 556–559.

Zhang, Y., Liu, T., Meyer, C.A., Eeckhoute, J., Johnson, D.S., Bernstein, B.E., Nusbaum, C., Myers, R.M., Brown, M., Li, W., and Liu, X.S. (2008). Model-based analysis of ChIP-Seq (MACS). Genome Biol. *9*, R137.

STAR★METHODS

KEY RESOURCES TABLE

REAGENT or RESOURCE	SOURCE	IDENTIFIER
Antibodies		
goat-anti-NANOG	R&D Systems	Cat# AF1997
rabbit-anti-OCT4	Abcam	Cat# AB19857
rabbit-anti-H3K4me1	Abcam	Cat# AB8895
rabbit-anti-H3K27ac	Abcam	Cat# AB4729
rabbit-IgG	Life Technology	Cat# 10500C
goat-IgG	Santa Cruz Biotechnology	Cat# SC-2028
donkey-anti-goat conjugated to Alexa fluor488	Invitrogen	Cat# A11055
donkey-anti-rabbit conjugated to Alexa fluor568	Invitrogen	Cat# A10042
donkey-anti-goat-IRDey680	Li-Cor	Cat# 926-68074
rabbit-anti-Laminin B	Abcam	Cat# AB16048
Bacterial and Virus Strains		
MegaX DH10β E.coli bacteria	Invitrogen	Cat# C6400-03
Chemicals, Peptides, and Recombinant Proteins		
mTesR1 medium	Stem Cell Technology	Cat# 05850
Y-27632	Cambridge bioscience	Cat# SM02-10
knockout DMEM	Invitrogen	Cat# 10829018
knockout serum replacement	Invitrogen	Cat# 10828028
human insulin	Sigma	Cat# I9278
recombinant human LIF	Millipore	Cat# LIF1010
recombinant bFGF	Peprotech	Cat# 100-18B
recombinant TGF-β	Peprotech	Cat# 100-21C
PD0325901	Axon Medchem	Cat# 1408
CHIR99021	Axon Medchem	Cat# 1386
SP600125	Abcam	Cat# ab120065
SB203580	Abcam	Cat# ab120638
G06983	Abcam	Cat# ab144414
TrypLE	Invitrogen	Cat# 12604021
0.5mM EDTA	Invitrogen	Cat# 15575020
Accutase	Invitrogen	Cat# A1110501
Protease inhibitor complex	Roche	Cat# 04693116001
Protein Dynabeads G	Life Technology	Cat# 10004D
Lipofectamine 3000	Life Technology	Cat# L3000015
Oligo (dT)25 beads	Life Technology	Cat# 61002
DNaseI	Life Technology	Cat# 18068-015
SuperscriptIII	Life Technology	Cat# 18080-044
Critical Commercial Assays		
2x Takyon qPCR master mix	UF-NSMT-B0701	Cat# Takyon
NEB Next ChIP-seq library preparation kit	NEB	Cat# E6200
NEB Next ChIP-seq library preparation kit	NEB	Cat# E6240
Illumina index primers	NEB	Cat# 7335
Illumina index primers	NEB	Cat# 7500

(*Continued on next page*)

Continued

REAGENT or RESOURCE	SOURCE	IDENTIFIER
NEB Next Q Hot start high fidelity master mix	NEB	Cat# M0543S
Phusion Polymerase	NEB	Cat# M0530L
Dual Glo luciferase kit	Promega	Cat# E2920
Deposited Data		
Raw and analyzed data	This study	GEO: GSE99631
See Table S1 for an overview of generated datasets	This study	N/A
See Table S2 for an overview of published enhancer datasets used in this study	N/A	N/A
Human reference genome, NCBI build 37, GRCh37/hg19	Genome Reference Consortium	https://www.ncbi.nlm.nih.gov/grc/human
Published primed HUES64 human ESC RNA-seq	Gifford et al., 2013	https://ars.els-cdn.com/content/image/1-s2.0-S0092867413005138-mmc2.xlsx
Published primed H9 human ESC RNA-seq	Takashima et al., 2014	https://ars.els-cdn.com/content/image/1-s2.0-S009286741401099X-mmc3.xlsx
Published primed H1 human ESC RNA-seq	Ji et al., 2016	https://ars.els-cdn.com/content/image/1-s2.0-S1934590915005056-mmc2.xlsx
Published microarray data primed and naive hESCs	Gafni et al., 2013	GEO: GSE46872
Published putative human ESC enhancers	Hawkins et al., 2011	https://media.nature.com/original/nature-assets/cr/journal/v21/n10/extref/cr2011146x3.xls
Published putative human ESC enhancers	Rada-Iglesias et al., 2011	https://media.nature.com/original/nature-assets/nature/journal/v470/n7333/extref/nature09692-s2.xls
Published putative human ESC enhancers	Xie et al., 2013	https://ars.els-cdn.com/content/image/1-s2.0-S0092867413004649-mmc2.xlsx
Experimental Models: Cell Lines		
H9 female human ESCs	N/A	D. Hay, Edinburgh
Oligonucleotides		
See Table S5	N/A	N/A
Recombinant DNA		
mammalian STARR-seq plasmid	Arnold et al., 2013	A. Stark, Vienna
STARR-seq plasmid with AML promoter	This study	N/A
STARR-seq plasmid with CML promoter	This study	N/A
eSpCas9(1.1)	Slaymaker et al., 2016	Addgene #71814
gRNA for CRISPR cloned in eSpCas9(1.1), see Table S5 for oligonucleotide sequences	This study	N/A
Software and Algorithms		
Skewer (v0.1.124)	Jiang et al., 2014	https://github.com/relipmoc/skewer
Bowtie2 (v2.2.4)	Langmead and Salzberg, 2012	http://bowtie-bio.sourceforge.net/bowtie2/index.shtml
BEDTools (v2.20.1)	Quinlan and Hall, 2010	https://github.com/arq5x/bedtools2
UCSC Genome Browser	Kent et al., 2002	http://genome.ucsc.edu/cgi-bin/hgGateway
MACS2 (v2.1.0.20150420)	Zhang et al., 2008	https://github.com/taoliu/MACS
R (v3.2.3)	R Development Core Team, 2008	https://www.r-project.org/
R: h2o (v3.16.0.2)	H2O.ai team, 2017	https://cran.r-project.org/web/packages/h2o/index.html
R: DESeq2 (v1.10.1)	Love et al., 2014	https://www.bioconductor.org/packages/release/bioc/html/DESeq2.html

(*Continued on next page*)

Continued

REAGENT or RESOURCE	SOURCE	IDENTIFIER
R: changepoint(v2.2.2)	Killick, 2014	https://cran.r-project.org/web/packages/changepoint
R: LOLA (v1.0.0)	Sheffield and Bock, 2016	https://doi.org/10.1093/bioinformatics/btv612
R: BSgenome (v1.38.0), BSgenome.Hsapiens.UCSC.hg19 (v1.4.0)	N/A	https://bioconductor.org/packages/release/data/annotation/html/BSgenome.Hsapiens.UCSC.hg19.html
R: GenomicRanges (v1.22.4)	Lawrence et al., 2013	https://bioconductor.org/packages/release/bioc/html/GenomicRanges.html
CentriMo (v4.11.2)	Bailey and Machanick, 2012	http://meme-suite.org/
Enrichr API (version of January 2018)	Chen et al., 2013	https://doi.org/10.1093/nar/gkw377
GREAT (v3.0.0)	McLean et al., 2010	http://great.stanford.edu/public/html/
HOCOMOCO (v11)	Kulakovskiy et al., 2016	http://hocomoco11.autosome.ru/
FIMO (v4.10.2)	Grant et al., 2011	http://meme-suite.org/doc/fimo.html
Galaxy (v17.09)	Afgan et al., 2016	https://usegalaxy.org/
ROSE (v0.1)	Whyte et al., 2013	http://younglab.wi.mit.edu/super_enhancer_code.html
Other		
Supplementary resource website	This study	http://hesc-enhancers.computational-epigenetics.org

CONTACT FOR REAGENT AND RESOURCE SHARING

Further information and requests for resources and reagents should be directed to and will be fulfilled by the Lead Contact, Ian Chambers (ichambers@ed.ac.uk)

EXPERIMENTAL MODEL AND SUBJECT DETAILS

Cell lines

H9 female human ESCs were a gift of David Hay (Edinburgh). All cells were regularly karyotyped and checked for the presence of mycoplasm.

Cell Culture conditions

H9 human ESCs were cultured on Matrigel coated cell culture plates, using mTesR1 medium (Stem Cell Technology, 05850). Cells were routinely split (ratio 1:3-1:4) using 0.5mM EDTA (Invitrogen, 15575020). For transfection, single cells were obtained by Accutase treatment (Invitrogen, A1110501), in the presence of Rock inhibitor, Y-27632 (10uM, Cambridge bioscience, SM02-10). For conversion to the naive state, cells were split on irradiated MEFs on gelatin coated plates and media was changed to NHSM media, as described by Gafni et al. (2013), containing knockout DMEM (Invitrogen), 20% knockout serum (Invitrogen), human insulin (Sigma, 12.5 μg ml-1 final concentration), 20 ng ml-1 recombinant human LIF (Millipore), 8 ng ml-1 recombinant bFGF (Peprotech) and 1 ng ml-1 recombinant TGF-β1 (Peprotech), 1 mM glutamine (Invitrogen), 1% nonessential amino acids (Invitrogen), 0.1 mM beta-mercaptoethanol (Invitrogen), penicillin-streptomycin (Invitrogen) and small molecule inhibitors: PD0325901 (1 μM, ERK1/2i, Axon Medchem); CHIR99021 (3 μM, GSKβi, Axon Medchem); SP600125 (10 μM, JNKi, Abcam ab120065) and SB203580 (10 μM, p38i,Abcam ab120638) Y-27632 (5 μM, ROCKi) and protein kinase C inhibitor G06983 (5 μM, PKCi, Abcam, ab144414). Cells were 1:10 passaged using TrypLE™ (Invitrogen, 12604021) in the presence of Rock inhibitor and maintained for more than 10 passages in NHSM media prior to analysis.

METHOD DETAILS

Experimental Design

All experiments were replicated. For the specific number of replicates done see either the figure legends or the specific section below. No aspect of the study was done blinded. Sample size was not predetermined and no outliers were excluded.

Chromatin immunoprecipitation

For chromatin immunoprecipitation, $2x10^7$ H9 primed or naive ESC were harvested in 9 mL of medium and cross-linked by addition of 270 μL 37% Formaldehyde (Sigma, final concentration of 1%), for 10 min at room temperature under rotation. 1 mL of 1.25 M

Glycine was added, cells were incubated on ice for 5 min and 3x washed with ice cold PBS. At this point, cross-linked cell pellets were snap-frozen and stored at −80°°C, or immediately processed for sonication. Prior to sonication, cells were resuspended in 1ml TE-I-NP40 (10mM TRIS-HCl pH 8, 1mM EDTA, 0.5% NP40, 1mM PMSF, 1x Protease inhibitor complex (PIC, Complete tablets, 04693116001, Roche)) incubated on ice for 5 min and centrifuged for 5 min at 2500 rpm at 4°C in a refrigerated bench top centrifuge (Eppendorf). Supernatant was removed and nuclei were resuspended in 1 mL ice-cold lysis buffer (50mM TRIS-HCl pH 8, 10mM EDTA, 1% SDS, 1mM PMSF, 1x PIC) and transferred to a 15 mL Falcon tube for sonication, using a Diagenode Bioruptor Next Gen (40 cycles of 30'' on, 30'' off). After transfer to an Eppendorf tube and centrifugation for 10 min at 13200 rpm at 4°C, chromatin solution was aliquoted and used for immunoprecipitation or snap-frozen and stored at −80°C. A 20 μl sample was taken and served as a total input control. For immunoprecipitation, Protein Dynabeads G (10004D, Life Technologies) were washed with PBS and incubated for 6 hours with 5 μg of antibody, at 4°C on a rotating wheel. Antibodies used were: goat-anti-NANOG (AF1997, R&D Systems), rabbit-anti-OCT4 (AB19857, Abcam), rabbit-anti-H3K4me1 (AB8895, Abcam) and rabbit-anti-H3K27ac (AB4729, Abcam); as a control, respective IgG antibodies were used (rabbit-IgG: 10500C, Life Technology, goat-IgG: SC-2028, Santa Cruz Biotechnology). After washing with PBS, antibody-coupled beads were incubated with 200 μL chromatin solution, diluted to a final volume of 2 mL with dilution buffer (167mM NaCl, 16.7mM TRIS-HCl pH 8.1, 1.2mM EDTA, 0.01% SDS, 1.1% Triton X-100, 1mM PMSF, 1x PIC), overnight at 4°C on a rotating wheel. Washing of beads was performed by incubation with ice-cold 1 mL of washing buffer, for 5 min, at 4°C on a rotating wheel, followed by removal of supernatant using a magnetic stand, for each of the following: 2x with wash buffer 1 (10mM TRIS-HCl pH 7.6, 1mM EDTA, 0.1% SDS, 1% Triton X-100, 0.1% NaDeoxychloate), 2x with wash buffer 2 (10mM TRIS-HCl pH 7.6, 1mM EDTA, 0.1% SDS, 1% Triton X-100, 0.1% NaDeoxychloate, 150mM NaCl), 2x with wash buffer 3 (250mM LiCl, 0.5% NP40, 0.1% NaDeoxychloate), 1x with TE 1x with 0.2% Triton X-100 and 1x with TE 1x, after which beads were resuspended in 100ul TE1x. Immunoprecipitated chromatin and total input control were decross-linked, by addition of 3 μL of 10% SDS and 5 μL Proteinase K (20 μg/μl, Roche) and 10 μL RNase A (50 μg/μl, Roche) to each tube and incubation overnight at 65°C on a shaking thermomixer block, 1400 rpm (Eppendorf). The next day, beads were briefly vortexed and supernatants were transferred to new tubes using the magnetic stand. 100 μL of TE1x containing 500mM NaCl was added to the beads and briefly vortexed, after which the supernatant was added to the first fraction of collected supernatant. Following Phenol / chloroform extraction, DNA was precipitated using 1 μL glycogen (20mg/ml), 1/10 vol NaOAc (3M) and 100% ice-cold Ethanol, at −20°C for 1 hour, followed by centrifugation at 13200 rpm for 1 hour at 4°C. After a final wash with 70% ethanol, the DNA pellet was dried and resuspended in 50 μL H_2O. Concentration of ChIP DNA was determined by Qubit measurement following manufacturer's instructions and sonication was assessed by gel-electrophoresis of total input DNA (target fragment size between 200 and 600 bp).

ChIP-qPCR

Concentration of ChIP and total input control DNA was assessed by Qubit measurement (LifeTech) according to manufacturer's instructions and was diluted to 2 ng/μl. 2 μL of DNA was used per qPCR reaction, using a 2x Takyon qPCR master mix (No ROX SYBR, UF-NSMT-B0701, Takyon). qPCR reactions were run on a Roche Lightcycler 480 II (Roche), using the following cycle conditions: 95°C 3 min, (95°C 10 s, 60°C 30 s, 72°C 25 s) x45, followed by a melting curve from 95° to 65°C. All data shown are averages of at least 2 biological replicates and 3 technical replicates. All primers used are shown in Table S5.

ChIP-seq, ChIP-STARR-seq plasmid library preparation

For ChIP-seq and ChIP-STARR-seq plasmid library generation, 10 ng of ChIP DNA was used as starting material. Using NEB Next ChIP-seq library preparation kit (E6200 or E6240, NEB), DNA was end-repaired, dA-tailed and adaptor-ligated according to manufacturer's instructions. After adaptor ligation and purification using AMPure-XP beads (0.8x, Beckman Coulter) and elution into 30 μL of 0.1xTE, 25 μL of the reaction product was used for ChIP-seq library preparation, by PCR amplification with Illumina index primers (7335 and 7500, NEB) using the NEB Next Q Hot start high fidelity master mix (M0543S, NEB) according to manufactures instructions (cycle conditions: 98°C 30 s, (98°C 10 s, 65°C 75 s) x15, 65°C 5 min, 4°C hold). After an additional round of AMPureXP bead purification, DNA was eluted in 0.1xTE without further size selection. Quality and quantity of the prepared ChIP-seq libraries was assessed on an Agilent Tapestation. All sequencing occurred on an Illumina HiSeq 2500 platform, using 50bp single-end sequencing.

The remaining 5 μL of purified adaptor ligated DNA were used for ChIP-STARR-seq plasmid library generation. Therefore, DNA was diluted to a total volume of 10 μL in 0.1xTE and used as an input in 8 × 50 μL PCR reactions using Phusion Polymerase, High-fidelity buffer (M0530L, NEB) and primers 147 STARRseq libr FW (TAGAGCATGCACCGGACACTCTTTCCCTACACGACGCTCTTCC GATCT) and 148 STARRseq libr RV (GGCCGAATTCGTCGAGTGACTGGAGTTCAGACGTGTGCTCTTCCGATCT) (Arnold et al., 2013), which prime on the adaptor sequences and add a 5′and 3′ 15 nucleotide homology sequence to the reaction products which are used for Gibson assembly. After PCR amplification (cycle conditions: 98° 2 min, (98°C 10 s, 62°C 30 s, 72°C 30 s) x 15, 72°C 5 min, 4°C hold), PCR reactions were pooled, purified using AMPure XP beads (1.8x), eluted in 30 μL 0.1xTE and used for Gibson assembly. Therefore, 15 μg of the mammalian STARRseq plasmid (a kind gift of A.Stark) (Arnold et al., 2013) were digested with AgeI-HF and SalI-HF (NEB) for 8h at 37°C, column purified (Nucleospin purification columns, 740609250, Machery-Nagel), eluted in 30 μl elution buffer and used as a vector in a Gibson reaction, using 2 μL of digested plasmid, 5 μL purified PCR product, 3 μL H20 and 10 μL of a home-made Gibson reaction (100mM Tris-HCl, 10mM MgCl2, 0.2 mM dNTP (each), 0.5U Phusion DNA polymerase (NEB), 0.16U 5′ T5 exonuclease (Epicenter), 2 Gibson reactions per library. After incubation at 50°C for 1 hour, Gibson reaction were pooled and precipitated by addition of 1 μL Glycogen (20 μg/μl, Roche, 1090139300), 5 μL NaOAc (3M) and 125 μL ice-cold 100% ethanol, incubation at −20°C for 1 hour and centrifugation for 1 hour at 13200 rpm at 4°C, followed by a final wash in 70% ethanol. After air

drying, DNA pellet was dissolved in 10 μL water and used for electroporation into electrocompetent MegaX DH10β E.coli bacteria (Invitrogen), according to manufacturer's instructions, using a Biorad pulser. A total of 5 electroporations per library were performed with each 2 μL of DNA. After recovery in 1 mL SOCS medium each, bacteria were grown for 1 hour at 37°C in a bacterial shaker in the absence of antibiotics. Then, bacteria were pooled together and 50 μL of a 1:100 and 1:10000 dilution was plated on Ampicillin containing Agar plates to enable estimation of the number of transformants after overnight growth at 37°C (Control electroporations with Mock-Gibson without addition of PCR product plated on Ampicillin, or digested STARRseq plasmid transformations on Ampicillin- and Ampicillin/Chloramphenicol-containing Agar plates were negative, confirming complete digestion of the STARR-seq plasmid and a functional Ccdb counter-selection in DH10βE.Coli). The remaining 5 mL of bacteria culture were incubated in a total volume of 2 l of LB-media supplemented with Ampicillin and allowed to grow for 16 hours in a bacterial shaker at 37°C. Plasmid DNA was isolated using a QIAGEN Maxiprep kit according to manufacturer's instructions and eluted in 500 μL 10mM Tris-HCl, pH 7.4. Concentration was determined by Nanodrop measurement. For BAC-STARR-seq of super enhancer regions, three BAC clones (RP11-357D8, RP11-100L8, RP11-713N22) were ordered at the BAC PAC resource center from CHORI. DNA was isolated according to standard procedures, mixed in equimolar quantities and subjected to sonication, after which 10 ng was used for end-repair, adaptor ligation and cloning of plasmid libraries as described above for the ChIP-STARR-seq.

Transfection of plasmid libraries

Primed and naive H9 ESCs were transfected using either Nucleofection (Lonza, VPH-5022), or using Lipofectamine 3000 according to manufacturer's instructions. For each transfection, 6-10 million cells were used (approximately 2.5-4.2 x10^8 cells in total) and transfected with 8 μg of plasmid library DNA and 500 ng pmCherry-N1 plasmid (Clonetech) as transfection control. Cells were incubated in 10 cm dishes and 24h post-transfection, single cells were harvested and subjected to FACS. Non-transfected cells were used to set sorting gates, DAPI was used as a marker for dead cells. All percentages mentioned are relative to the fraction of DAPI-negative, single cells.

ChIP-STARR-seq RNA and DNA samples

A minimum of 400,000 GFP-positive, sorted cells were used to isolate total RNA using Trizol (Thermo Fisher) according to manufacturer's instructions. On average, 2 million GFP-positive cells were used per sample. The mRNA fraction was captured using Oligo (dT) 25 beads (61002, Life Technologies) and DNaseI treated (18068-015, Life Technologies), followed by reverse transcription using 2 μL SuperscriptIII (18080-044, Life Technologies) using a GFP-mRNA specific primer (149 STARRseq rep RNA cDNA synth, CAAACT CATCAATGTATCTTATCATG) at 50°C for 90 minutes, in a total reaction volume of 21 μl. To repress residual plasmid DNA contamination, cDNA was PCR amplified using a combination of primers (152 STARR reporter specific primer 2 fw, GGGCC AGCTGTTGGGGTG*T*C*C*A*C and 153 STARR reporter specific primer 2 rv, CTTATCATGTCTGCTCGA*A*G*C, where * represent phosphorothioate bonds) spanning a synthetic intron in the STARR-seq plasmid, as previously described (Arnold et al., 2013). PCR was performed with Phusion polymerase and High-fidelity buffer, in 6 × 50 μl reactions (cycling conditions: 98°C 2 min, (98°C 10 s, 62°C 30 s, 72°C 70 s) x15, 72°C 5 min, 4°C hold). PCR reactions were pooled, purified using AMPureXP beads (1.0x) and eluted in 18 μL 0.1xTE. Absence of significant plasmid contamination in the PCR amplified cDNA was assessed by qPCR using a primer-set amplifying an amplicon from the STARR-seq plasmid backbone (161 STARRseq detect plasmid backbone qPCR fw, CATCATCGG GAATCGTTCTT, and 162 STARRSeq detect plasmid backbone qPCR rv, TGAAGATCAACTGGGTGCAA), relative to a primer-set amplifying GFP (154 STARRseq GFP fw, ACGGCCACAAGTTCTCTGTC, and 155 STARRseq GFP rv, GCAGTTTGCCAGTAGTG CAG). PCR amplified cDNA was then used in a second round of PCR to add Illumina index primers (7335, 7500, NEB) using priming on the adaptor sequences added during the plasmid library generation. PCR was performed in 1-4x 50 μL reactions using Phusion polymerase and High-fidelity buffer (NEB)(cycling conditions: 98°C 2 min, (98°C 10 s, 65°C 30 s, 72°C 30 s) x13, 72°C 5 min, 4°C hold), after which PCR reactions were pooled, purified using AMPureXP beads (1.0x) and eluted in 15 μL 0.1xTE. Corresponding plasmid libraries were similarly amplified in a nested PCR, using primers detecting the STARR-seq plasmid (160 STARR reporter specific primer for plasmid DNA fw, GGGCCAGCTGTTGGGGTG, and 153 STARR reporter specific primer 2 rv, CTTATCATGTCTGCTC GA*A*G*C, where * represent phosphorothioate bonds) and Illumina index primers. In addition to sequencing libraries prepared from plasmid maxiprep DNA, we also sequenced plasmid libraries reisolated from transfected ESCs. For this, we transfected H9 ESCs as described above and harvested non-sorted cells 24h post-transfection, followed by plasmid reisolation using a QIAGEN miniprep isolation kit and sequencing library preparation. Quantity and quality of generated sequencing libraries was assessed on an Agilent Tapestation. All sequencing occurred on an Illumina HiSeq 2500 platform, using 50bp or 125 bp paired-end sequencing. Up to 22 RNA samples were pooled on a single lane. During data-processing all reads were trimmed to 50bp length to improve consistency.

RT-qPCR

For RNA analysis of complete cultures, cells were lysed in Trizol (Thermo Fisher) and RNA was prepared according to manufacturer's instructions. 1 μg of RNA was treated with DNaseI (Invitrogen) to remove genomic DNA contamination and cDNA was obtained through reverse transcription using SuperScriptIII (Invitrogen) in the presence of RNaseOUT (Invitrogen). cDNA was diluted in DEPC-treated water to a final volume of 200 μL and 2 μL of cDNA was used per qPCR reaction, using a 2x Takyon qPCR master mix (No ROX SYBR, UF-NSMT-B0701, Takyon). qPCR reactions were run on a Roche Lightcycler 480 II (Roche), using the following cycle conditions: 95°C 3 min, (95°C 10 s, 60°C 30 s, 72°C 25 s) x45, followed by a melting curve from 95° to 65°C. All data shown are averages of at least 2 biological replicates and 3 technical replicates, normalized to TBP. All primers used are shown in Table S5.

Immunostaining

Cells were grown on culture dishes suitable for confocal microscopy (Ibidi, 81156) and fixed using 4% v/v Paraformaldehyde at room temperature for 10 min. After permeabilisation using 0.3% Triton/PBS and incubation with blocking solution (1% BSA, 3% Donkey serum, 0.1% triton in PBS), cells were incubated with primary antibody O/N at 4°C. After washing with PBS, cells were incubated with secondary antibody at RT for 1h, washed and counterstained with DAPI. Imaging occurred on a Leica SP8 STED-CW confocal microscope and images were processed using ImageJ software. Antibodies used are: goat-anti-NANOG (1: 200, AF1997, R&D Systems), rabbit-anti-OCT4 (1: 200, AB19857, Abcam). Secondary antibodies were Donkey-anti-goat conjugated to Alexa fluor488 (1:800, A11055, Invitrogen) and Donkey-anti-rabbit conjugated to Alexa fluor568 (1:1000, A10042, Invitrogen).

Western blotting

Whole cell protein extracts were isolated and western blotting was performed using standard procedures using pre-cast 10% Bis-Tris Bolt gels (Invitrogen). Primary antibody used was goat-anti-NANOG (1: 500, 1 μg/ml, AF1997, R&D Systems), secondary antibody conjugated to fluorophores was donkey-anti-goat-IRDey680 (1:500, 926-68074, Li-Cor). Rabbit-anti-Laminin B (1:1000, AB16048, Abcam) served as a loading control and was detected by chemi-iluminescence. Imaging occurred on an Odyssey imager (Li-Cor).

Luciferase assays

Enhancer sequences were PCR amplified from human genomic DNA using Phusion polymerase and cloned by Gibson assembly into a KpnI-NheI linearized Pgl3 promoter luciferase vector. For primer sequences, see Table S5**.** All constructs were sequence-verified by Sanger sequencing and co-transfected with a Renilla expressing plasmid using Lipofectamin 3000 into H9 ESCs. 48h post-transfection illuminescence was assessed using the Dual Glo luciferase kit (E2920, Promega) according to manufacturer's instructions, on a Promega Glumax Multidection system. All data shown are average from at least two biological replicates and two technical replicates, representing fold-change in luciferase activity compared to empty vector controls and normalized for Renilla transfection control.

Alternative promoter STARR-seq constructs

To replace the SCP1 minimal promoter from the original STARR-seq plasmid (Arnold et al., 2013), the plasmid was linearized by restriction digestion using KpnI-ApaI (NEB) and used to ligate annealed oligonucleotides, coding for the adenovirus major late (AML) or CMV IE core promoter (Juven-Gershon et al., 2006). Test enhancer sequences were introduced by PCR amplification and Gibson assembly as done during library cloning. All constructs were verified by Sanger sequencing. Oligonucleotide sequences are given in Table S5. Constructs (1 μg of each plasmid) were transfected in H9 primed ESCs cultured in 6-well plates using Lipofectamine 3000 and fluorescents was assessed using flow cytometry. Shown are the results for two independent experiments (analyzing > 30.000 GFP positive cells each), comparing all identical tested enhancer sequences in constructs with the SCP1, AML or CMV minimal promoter transfected in parallel.

CRISPR/Cas9 genome editing

Oligonucleotides for gRNAs (Table S5) flanking the tested enhancers were annealed and cloned into a BbsI digested spCas9 plasmid, from which the gRNAs are separately expressed together with a eSpCas9(1.1)-t2a-mCherry or eSpCas9(1.1)-t2a-GFP (modified from Addgene plasmid #71814) (Slaymaker et al., 2016). All plasmids were sequence verified and 1 μg of each gRNA was used to transfect primed H9 ESCs in a 6-well plate using Lipofectamine 3000. 48h post-transfection, mCherry and GFP double positive cells were FACS sorted and cells were plated at low density in 10 cm dishes coated with Matrigel in conventional mTesR1 ESC medium. Emerging clones were expanded and genotyped by PCR using primers flanking the gRNA targets (Table S5). For the pos3_ID1 enhancer, a nested PCR using outer and inner primers was performed. All candidate clones were validated by Sanger sequencing of PCR products and correct clones were expanded.

QUANTIFICATION AND STATISTICAL ANALYSIS

ChIP-seq and ChIP-STARR-seq data processing

We trimmed possible adaptor contaminants from reads using Skewer (Jiang et al., 2014). Trimmed reads were then aligned to the GRCh37/hg19 assembly of the human genome using Bowtie2 (Langmead and Salzberg, 2012) with the "*–very-sensitive*" parameter. Genome browser tracks were created from all aligned reads with the *genomeCoverageBed* command in BEDTools (Quinlan and Hall, 2010) and normalized such that each value represents the read count per base pair per million uniquely mapped reads. Finally, the UCSC Genome Browser's *bedGraphToBigWig* tool was used to produce a bigWig file.

ChIP-STARR-seq enhancer activity levels

For ChIP-seq and plasmid DNA-seq libraries, peak calling was performed with MACS2 version 2.1.0.20150420 (Zhang et al., 2008) with default parameters (narrow peak calling, fragment length detection from libraries, genome size $2.7x10^9$ bp, FDR < 0.05), using the respective input samples as background. Significant peaks (FDR < 0.05) were fixed to a width of 500 bp from the peak summit for

transcription factors and 1000 bp for histone modifications. Peaks overlapping blacklisted features as defined by the ENCODE project (Hoffman et al., 2013) were removed. ChIP-seq peaks are given in Data S1.

To define a non-redundant set of enhancers to compare in our analysis of ChIP-seq, plasmid DNA-seq and ChIP-STARR RNA-seq samples, we produced a set of regions by merging all peaks across cell types and experiment types (ChIP-seq and plasmid DNA-seq). This operation results in regions that can be very large. To preserve high genomic resolution for our analysis, large regions were split in half recursively until all regions were at most 1000 bp long. All further analysis were performed on these scaffold regions.

We initially quantified the intensity of ChIP-seq, plasmid DNA-seq and ChIP-STARR RNA-seq datasets in the enhancer peak regions by counting the number of aligned fragments (only properly paired, concordantly aligning and uniquely mapping fragments – i.e., both mate reads mapped to same chromosome with MAPQ > = 30 – were kept) overlapping each enhancer region. To get a more accurate and precise measure of plasmid reporter intensity for further analysis, we then made use of our paired-end sequencing data to unequivocally link RNA-seq reads to the plasmid that they came from. To do so, we matched RNA-seq reads to plasmid reads with the exact same start coordinate of the first read and the exact same end coordinate of the second read. Comparing the counts for both made it possible to define a measure of RNA-seq activity relative to the abundance of plasmids in the. To avoid distortion by differences in sequencing depth, we normalized the raw read counts for each plasmid library and all RNA-seq datasets derived from transfections of this library together using DESeq2 (Love et al., 2014). The ratio of normalized RNA-seq and (plasmid) DNA-seq reads was used as a measure of enhancer activity (reads per plasmid, RPP). We then calculated the mean RPP of replicate measurements for the same plasmid position and used the maximum observed RPP value per region as an estimate of enhancer-peak-level activity. Since our individual replicate datasets were sparse, with the same plasmids infrequently measured in both replicates, but our overall coverage of enhancers was much better, we used RPP from all datasets generated in the same cell type (so specific to either primed or naive H9 ESCs) for this purpose. We could do so because the ChIP-STARR-seq plasmid libraries are independent from the antibody target used to pull down the enriched DNA fragments, thus the plasmids in all libraries jointly report the activity of the same genome. To objectively define a threshold for discriminating highly active and inactive genome regions, we looked at the curve of RPP ranks versus RPP values (Figure 2C) and defined points of change in the mean and variation of the data using the *changepoint* package in R (Killick, 2014). The highest value was used as a threshold for active enhancers (θ = 138). The coordinates of all genome regions assessed with activity calls are given in Table S2 and Data S1.

Motif enrichment analysis for ChIP-seq data

For *de novo* motif discovery (Figure S5), BED files of ChIP-seq datasets were generated with 500 bp sequences centered on the narrow ChIP-seq peak, and used for motif enrichment analysis using CentriMo (http://meme-suite.org/) (Bailey and Machanick, 2012), using default settings.

Assignment of enhancers to genes

We used GREAT, version 3.0.0 (McLean et al., 2010) to assign regulatory elements identified in ChIP-STARR-seq to their putative target genes, using the following settings: basal plus extension, proximal 5kb upstream and 1kb downstream, plus distal up to 100kb. Publically available, processed RNA-seq data from primed human ESCs were downloaded (Gifford et al., 2013; Ji et al., 2016; Takashima et al., 2014) and their RPKM value distribution was plotted for the various ChIP-STARR-seq regions grouped by activity in RPP. For naive ESCs, we used publically available microarray data from the original study describing gene expression in naive cells cultured under NHSM conditions (Gafni et al., 2013).

Comparison to previously published enhancers

The coordinates of putative enhancers were obtained from the supplemental data of Hawkins et al. (2011), Rada-Iglesias et al. (2011), and Xie et al. (2013), and when necessary converted to the hg19 version of the human genome using the liftOver tool. Overlapping enhancers were merged into 76,666 putative enhancers and joint to our ChIP-STARR-seq enhancers using GenomicRanges (Lawrence et al., 2013) in R (see Figure S4A; Table S2). We refer to those enhancers that overlapped with previously published enhancers and showed a ChIP-STARR-seq activity of RPP > = 138 as the core enhancer module (n = 7,948). Conversely, we refer to active enhancers (RPP > = 138) that did not overlap with the previously published enhancers as the extended enhancer module (n = 24,405).

Functional enrichment analysis

To help understand the function and relevance of different groups of enhancers, we used three types of functional enrichment analysis (Table S3).

(a) We used LOLA (Sheffield and Bock, 2016) to determine the relative over-representation of ChIP-seq peaks related transcription factor binding and other elements of known regulatory function. To this end, we used the *codex*, *encode_tfbs*, and *encode_segmentation* databases contained in the LOLA Core database and tested for the enrichment of overlap in genome regions with a specific level of activity (high, low or inactive) over the background of all ChIP-STARR-seq peaks.

(b) We also used the Enrichr API (January 2018 version) (Chen et al., 2013) to test genes linked to enhancers of interest for significant enrichment in numerous functional categories. To comply with the web interface, we considered the 1000 genes closest to the tested peaks for enrichments. In all plots, we report the "combined score" calculated by Enrichr, which is a

product of the significance estimate and the magnitude of enrichment (combined score $c = log(p) * z$, where p is the Fisher's exact test p value and z is the z-score deviation from the expected rank).

(c) We additionally used the GREAT web interface (version 3.0.0) (http://great.stanford.edu/public/html/) (McLean et al., 2010) for gene ontology analysis, using the following settings: basal plus extension, proximal 5kb upstream and 1kb downstream, plus distal up to 100kb, including curated regulatory domains, and whole genome (hg19) as background.

Machine learning

We used the random forest classifier implementation in the h2o R package (https://github.com/h2oai/h2o-3) to train models for predicting enhancer activity ("Active" versus "Inactive") in primed and naive ESCs and to discriminate enhancers from the Core and Extended module ("Core" versus "Extended"). Three types of features based on the DNA underlying each ChIP-STARR-seq region were used as inputs: (a) sequence conservation. The maximum PhastCons score from overlaps with the UCSC Golden Path reference was used per region; (b) GC content calculated from alphabet frequency in R; (c) dinucleotide frequencies calculated with the bioconductor package *Biostrings*), taking the maximum on either forward or reverse strand; and (d) occurrence of known motifs from the HOCOMOCO database (Kulakovskiy et al., 2016) (v11; limited to "excellent" [A] and "good" [B] quality motifs). The tool FIMO (v4.10.2) (Grant et al., 2011) was used (parameters: *–no-qvalue–text–bgfile motif-fil*) to scan DNA sequences for these motifs and regions with at least one hit ($p < 0.05$) were counted. Each classifier was trained on balanced classes from the complete set of ChIP-STARR-seq regions (excluding missing RPP values) or on all active enhancers (RPP > = 138; for Core/Extended discrimination) using 10-fold cross-validation and evaluation 500 trees with 50 features sampled at each split and a maximum depth of 10 (parameters: *mtries = 50, nfolds = 10, keep_cross_validation_predictions = T, balance_classes = T, ntrees = 500, max_depth = 10*).

Enrichment analysis for transposable elements

The UCSC RepeatMask (hg19) was downloaded from the UCSC Table Browser, imported into Galaxy (https://usegalaxy.org/) (Afgan et al., 2016) and joined to the ChIP-STARR-seq activity calls for primed or naive ESCs. The number of overlaps of each type of repeat ($n_{overlaps}$) with all ChIP-STARR-seq regions (n) was used to calculate the relative frequency ($f_{all} = n_{overlaps}/n$). Multiplication of the relative frequency with the number of regions (n_{test}, e.g., $n_{active,primed}$) in any tested groups yields the expected frequency (E). This number was compared with the actual observed frequency in the subgroups ($f_{test} = n_{overlap,test}/n_{test} = O$) to calculated the observed versus expected ratio (O/E). We considered repeats with $O/E < 0.5$ as depleted, or $O/E > 2$ as enriched. For the subsequent data interpretation we only focused on transposable elements that were present multiple times ($n_{overlap} > 15$) in all ChIP-STARR-seq regions.

Super-enhancer analysis

To call super-enhancers in primed and naive H9 ESCs, we used the ROSE software (v0.1) (Whyte et al., 2013) to combine ("stitch") H3K27ac ChIP-seq peaks within 12.5 kb of each other and excluding 2.5 kb around known transcription start sites. An alternate analysis was also run with stitching distance d = 0 for comparison. We then asked the software to quantify the ratio of the H3K27ac ChIP-seq signal in primed and naive ESCs over the total input control and to call super-enhancers. The coordinates of all stitched enhancers, as well as primed and naive super-enhancers are given in Data S1.

Statistics for qPCR and luciferase assays

qPCR and luciferase assay figures were plotted and statistics were calculated using GraphPad Prism 5 software, $p < 0.05$ was considered significant. Statistical tests used are indicated in the figure legends. For the qPCR analysis of CRISPR deleted enhancer clones in Figure 2E, we calculated expression as follows: in each graph (with the exception of TBX3), average results for the indicated enhancer deletion (heterozygous (+/−) or homozygous (−/−) as indicated) are plotted relative to wild-type, n = number of cell lines per genotype. Wild-type controls consisted of H9 parental, two untransfected H9 clones and all remaining clones that were wild-type for the respective allele. Genes assessed were the presumed target gene and four randomly selected genes. For the TBX3 intronic deletion, three H9 wt and three −/− deletion clones were assessed for three amplicons detecting TBX3 mRNA and two flanking genes. All measurements occurred at two different passages, in two independent cultures measured in duplicate.

DATA AND SOFTWARE AVAILABILITY

Data availability

High-throughput sequencing data generated in this study have been submitted to the Gene Expression Omnibus (GEO): accession code GEO: GSE99631, to the Sequence Read Archive (SRA): accession codes SRA: SRP108517, SRA: SRP108518, SRA: SRP108519, and SRA: SRP108520. A BioProject for this study has also been registered: PRJNA389108.

ADDITIONAL RESOURCES

Additional data, an interactive search tool for active enhancers in the proximity of genes and the genome browser track hub providing raw and processed ChIP-STARR-seq data for interactive visualization and processing with online tools such as Galaxy, are available from a supplemental website under the following URL: http://hesc-enhancers.computational-epigenetics.org.

Cell Stem Cell, Volume *23*

Supplemental Information

Functional Dissection of the Enhancer Repertoire in Human Embryonic Stem Cells

Tahsin Stefan Barakat, Florian Halbritter, Man Zhang, André F. Rendeiro, Elena Perenthaler, Christoph Bock, and Ian Chambers

Supplementary Information

Functional dissection of the enhancer repertoire in human embryonic stem cells

Tahsin Stefan Barakat, Florian Halbritter, Man Zhang, André F. Rendeiro, Elena Perenthaler, Christoph Bock and Ian Chambers

Supplemental Inventory

Supplemental Figure legends

Supplemental Figures:

Figure S1, related to Figure 1: ChIP-seq in primed H9 human embryonic stem cells
Figure S2, related to Figure 1: Overview of generated datasets
Figure S3, related to Figure 2: ChIP-STARR-seq in primed H9 ESCs
Figure S4, related to Figure 4: Core and extended enhancer module
Figure S5, related to Figure 5: Conversion of primed to naive H9 human embryonic stem cells
Figure S6, related to Figure 5: ChIP-STARR-seq and functional enhancers in naive ESCs
Figure S7, related to Figure 7: Super-enhancers and ChIP-STARR-seq in primed and naive ESCs

Supplemental Figure Legends

Figure S1, related to Figure 1: ChIP-seq in primed H9 human embryonic stem cells

A) Brightfield microscopy of a representative colony of H9 ESCs cultured on Matrigel in standard ESC culture conditions.

B) Immunofluorescence of primed H9 ESCs for NANOG (green) or OCT4 (red); DNA is stained with DAPI (blue).

C) ChIP-qPCR in primed H9 ESC with anti-NANOG, anti-OCT4 or rabbit IgG at known OCT4 and NANOG binding sites in *SCGB3A2*, *SMARCA* (Kunarso et al., 2010) and *XIST* (left) or with anti-H3K4me1, anti-H3K27ac or rabbit IgG at binding sites near *FGFR1* (at central and flanking locations), *POU5F1* (at central and flanking locations), *CD9*, *SCGB3A2*, and *SMARCA* (Rada-Iglesias et al., 2011) (right). The mean fold-enrichment is shown relative to total input control DNA, normalized to a non-bound site in *ACTB* (for OCT4 and NANOG), or *NCAPD2* (for H3K4me1 and H3K27ac). Error bars indicate standard deviations; n= 3.

D) Venn diagrams of the overlap between ChIP-seq peaks in primed ESCs, indicating the cell line and study for NANOG, OCT4, H3K4me1 and H3K27ac (Ji et al., 2016; Gafni et al., 2013; Gifford et al., 2013; Kunarso et al., 2010; Lister et al., 2009 and this study) . The numbers indicate overlapping peaks.

E) Heatmap contrasting gene expression of immune response genes in human ESCs as determined by RNA-seq (Muerdter et al., 2018). Data from (Gifford et al., 2013; Ji et al., 2016; Takashima et al., 2014)

Figure S2, related to Figure 1: Overview of generated datasets

A) Summary table of the high-throughput sequencing datasets generated in this study, indicating the number of ChIP-seq, plasmid (corresponding to ChIP-STARR-seq or BAC-STARR-seq plasmid libraries prior to transfection) and isolated plasmid (plasmid libraries 24h after transfection) samples, as well as RNA-seq data from GFP-positive primed and/or naive ESCs (RNA: primed and RNA: naive, respectively).

B) Pearson correlation matrix of samples from the indicated ChIP-seq, plasmid libraries and isolated plasmid libraries from primed ESCs. Rows and columns have been arranged by hierarchical clustering with complete linkage.

C-D) Scatterplots contrasting normalized read counts (reads per million) per peak for different datasets. The Pearson correlation coefficient (*r*) for each comparison is indicated for each plot.

C) Comparison between ChIP-seq, DNA-seq of ChIP-STARR-seq plasmid libraries and two replicates of DNA-seq for isolated plasmid libraries post transfection, for OCT4, NANOG, H3K37ac, H3K27ac and genomic DNA (input).

D) Comparison between ChIP-STARR-seq plasmid libraries prior to transfection and the corresponding RNA-seq read counts generated from two replicates of GFP-positive cells after transfection.

E) Pearson correlation coefficients (*r*) between replicate STARR-RNA-seq measurements from the same pool of ESCs transfected with the same ChIP-STARR-seq library, shown as a function of minimum read count in both replicates (0 = unfiltered, 1 = at least one read from the same plasmid measured in each replicate, etc.).

Figure S3, related to Figure 2: ChIP-STARR-seq in primed H9 ESCs

A) Bar graph showing the mean GFP intensity as measured by flow cytometry for positive or negative ChIP-STARR-seq regions cloned into STARR-seq plasmids and used in transfection of H9 primed ESCs. Average results for two experiments are shown. Three different minimal promoters were assessed for each tested sequence. GFP mean intensity was indistinguishable from non-transfected cells for negative sequences in all constructs.

B) Boxplots showing the distribution of RNA-seq RPKM values of genes associated with enhancers grouped by activity level. Boxplots represent the interquartile range (IQR), the line is the median, whiskers extend to 1.5xIQR and outliers are indicated as dots. The numbers on the x-axis indicate thresholds on the RPP activity level; RPKM, reads per kilobase million. RNA-seq datasets were from the following studies: H1 (Ji et al., 2016); HUES64 (Gifford et al., 2013).

C) Distribution of active (RPP ≥138) and inactive sequences (RPP <138) in ChIP-STARR-seq regions overlapping with ChIP-seq peaks of the indicated factor or combination of factors.

D) PCR genotyping of H9 ESC wild type (WT) and targeted clones (numbers), that were transfected with Cas9 and gRNAs to delete DNA sequences with enhancer activity detected by ChIP-STARR-seq (*pos*, top) or inactive (*neg*, bottom) sequences. The putative interacting gene is indicated. Primers used for genotyping are located outside the gRNA targets; wt = wt allele, ko = knockout allele.

E) Relative enrichment of DNA-binding proteins (DBPs) from the ENCODE database (2012) in inactive genome regions, as well as active enhancers (compare to **Fig. 2G**). Shown are the log_2-odds ratios between observed percentages of enhancers overlapping binding sites of each given DBP in the respective groups over the percentage of overlaps in the entire enhancer dataset. Each dot represents one ChIP-seq dataset for the given DBP and the lines connect the most extreme dot with zero for visualization. For each category, the eight most enriched

DBPs are shown ranked by their mean log-odds ratio. ChIP-seq datasets produced from ESCs are indicated as dots and those from other cell sources as crosses. Enrichments were calculated using LOLA (Sheffield and Bock, 2016).

F) Smooth line plots showing the proportion of active plasmids (RPP ≥138) of all plasmids measured at the indicated distance from the peak center for ChIP-seq binding sites of factors from the CODEX database (Sanchez-Castillo et al., 2015) found preferentially associated at highly active enhancers (compare to **Fig. 2H**), averaged across all binding sites for the respective factor. The number of peaks (*n*) is indicated in each plot.

G) LOLA enrichment plots as in panel **E**, but showing instead the relative over-presentation of ENCODE chromatin segments from different cell lines in enhancers with different activity levels. E, enhancer; PF, promoter-flanking region; R, repressed; T, transcribed; TSS, transcription start site; WE, weak enhancer.

H) Line plots as in panel F, showing the proportion of active plasmids in a window around the center of repressed chromatin segments and enhancer chromatin segments from the ENCODE H1 chromatin segmentation (Hoffman et al., 2013).

Figure S4, related to Figure 4: Core and extended enhancer module

A) Illustration showing three source datasets (Hawkins et al., 2011; Rada-Iglesias et al., 2011; Xie et al., 2013) that contributed to the catalogue of putative ESC enhancers used in this study. We converted all enhancer coordinates to the same assembly (hg19) and then merged overlapping peaks resulting in a list of 76,666 putative enhancers.

B) Luciferase assay in primed ESCs for eight putative enhancers that did not show activity in ChIP-STARR-seq. The OCT4 proximal enhancer (PE) is tested as a positive control. Luciferase activity is reported as the fold enrichment in luciferase counts over empty vector, normalised to the Renilla transfection control. Error bars indicate standard deviations, n=2.

C) Illustration of the number of genes found in the proximity of core enhancer module (pink, left circle), or extended enhancer module (olive, right), or with enhancers of both types (white, overlap). Enhancer-gene assignments were performed with GREAT (McLean et al., 2010).

D) Receiver operating characteristic (ROC) curve illustrating random forest classifier performance.

E) Bar charts displaying the scaled variable importance of the top 25 sequence features used for the distinction of active enhancers from inactive regions. Motif IDs are shortened versions of the full ID from the HOCOMOCO database (v11) (Kulakovskiy et al., 2016). dinuc., dinucleotide frequency.

F) Boxplots of gene expression (RNA-Seq; log_2) in different tissues from the GTEx database (2013)for all genes linked to Core (pink) or Extended (olive) module enhancers or both (white). Boxplots represent the interquartile range (IQR), the line is the median, whiskers extend to 1.5xIQR and outliers are indicated as dots. RPKM, reads per kilobase million.

Figure S5, related to Figure 5: Conversion of primed to naive H9 human embryonic stem cells

A) Brightfield microscopy of a representative colony of H9 ESCs cultured in naive culture conditions on feeders for 10 passages showing a more dome-shaped colony morphology (compare to **Figure S1A**).

B) Immunofluorescence of naive H9 ESCs for NANOG (green) and OCT4 (red); DNA is stained with DAPI (blue).

C) Immunoblot analysis of NANOG and LAMININ B in primed and naive ESCs.

D) qRT-PCR of pluripotency-related genes in H9 ESCs cultured in primed or naive conditions (10 passages). Error bars indicate standard deviations; n=3.

E) ChIP-qPCR in naive H9 ESC with anti-NANOG, anti-OCT4 or rabbit IgG at three OCT4 and NANOG binding sites (from primed ChIP-seq data) in *SCGB3A2, SMARCA* (Kunarso et al., 2010) and *XIST* (left) or with anti-H3K4me1, anti-H3K27ac or rabbit IgG at binding sites near *FGFR1* (at central and flanking locations), *POU5F1* (at central and flanking locations), *CD9*, *SCGB3A2* and *SMARCA* (Rada-Iglesias et al., 2011) (right). The mean fold-enrichment is shown relative to total input control DNA, normalized to a non-bound site in *ACTB* (for NANOG and OCT4) or *NCAPD2* (for H3K4me1 and H3K27ac). Error bars indicate standard deviations; n= 3.

F-G) Venn diagrams of the overlap between ChIP-seq peaks in naive ESCs, indicating the cell line and study for **F)** H3K4me1 and **G)** H3K27ac (Gafni et al., 2013 and this study). The numbers indicate overlapping peaks.

H) Local motif enrichment analysis using CentriMo (Bailey and Machanick, 2012) for OCT4 ChIP-seq data generated in this study for primed (upper panel) and naive H9 ESCs (lower panel). Top-3 identified motifs and their p-values are indicated.

I) as **H,** but now for NANOG.

Figure S6, related to Figure 5: ChIP-STARR-seq and functional enhancers in naive ESCs

A-B) Scatterplots contrasting normalized read counts (reads per million) in naive H9 ESCs per peak for different datasets. The Pearson correlation coefficient (*r*) for each comparison is indicated for each plot.

A) Comparison of ChIP-seq datasets and the corresponding ChIP-STARR-seq plasmid library.

B) Comparison between ChIP-STARR-seq plasmid libraries prior to transfection and the corresponding RNA-seq read counts generated from two replicates of GFP-positive naive H9 ESCs after transfection.

C) Plot showing enhancer activity (normalized ratio of ChIP-STARR RNA over plasmids; $\log_2$) in naive H9 ESCs ranked from lowest to highest across all measured enhancers (union of all peak calls). Active enhancers and inactive regions are discriminated by a threshold (θ) as indicated in the plot by a dashed line. RPP, reads per plasmid million.

D) Distribution of active (RPP $\geq$138) and inactive sequences (RPP <138) in naive ESCs in ChIP-STARR-seq regions overlapping with ChIP-seq peaks of the indicated factor or combination of factors.

E) Relative enrichment of DNA-binding proteins (DBPs) from the ENCODE database (2012) in inactive genome regions, as well as lowly active and highly active enhancers in naive H9 ESCs (compare to **Fig. 5B**). Shown are the $\log_2$-odds ratios between observed percentages of enhancers overlapping binding sites of each given DBP in the respective groups over the percentage of overlaps in the entire enhancer dataset. Each dot represents one ChIP-seq dataset for the given DBP and the lines connect the most extreme dot with zero for visualization. For each category, the eight most enriched DBPs are shown ranked by their mean log-odds ratio. ChIP-seq datasets produced from ESCs are indicated as dots and those

from other cell sources as crosses. Enrichments were calculated using LOLA (Sheffield and Bock, 2016).

F) LOLA enrichment plots as in panel **C**, but showing instead the relative over-presentation of ENCODE chromatin segments from different cell lines in enhancers with different activity levels. E, enhancer; PF, promoter-flanking region; R, repressed; T, transcribed; TSS, transcription start site; WE, weak enhancer.

G) Barplots showing relative enrichment of H9 chromatin segment overlaps (Kundaje et al., 2015) in regions with ChIP-STARR-seq activity compared to inactive regions (see panel **A**). TSS, transcription start site; enh, enhancer; ZNF, zinc-finger protein.

H) Density scatter plot comparing ranked activity (RPP) in primed and naive ESCs. Point density is represented by color and contours are shown to emphasize dense regions. The majority of points is located either in the lower left (inactive) or upper left (active in primed and naive) section of the plot.

I) Plot equivalent to panel **A**, but showing ChIP-seq binding intensity instead of RPP. A change point analysis was performed to determine a threshold to distinguish strongly bound from weakly bound regions. RPM, reads per million.

J) Table showing the percentage of ChIP-STARR-seq regions with a certain activity (e.g., Inactive in primed and naive ESCs) that remain weakly bound (W→W), gain binding (W→S), lose binding (S→W), or remain strongly bound (S→S) in the transition from primed to naive ESCs according to the threshold shown in panel G. W, weak ChIP-seq binding; S, strong ChIP-seq binding; I, inactive ChIP-STARR-seq region; A, active ChIP-STARR-seq enhancer.

K) Functional enrichment analysis using Enrichr to test the relative over-representation of enhancers that lose ChIP-STARR-seq activity in the transition from primed to naive ESCs (Active→Inactive), that gain activity (Inactive→Active), or that remain inactive in both states

(Inactive→Inactive). Enrichments were calculated for genes near these regions to test for GO assignments (left) or for ENCODE and ChEA ChIP-seq experiments (right). The x-axis reports the combined score calculated by Enrichr.

L) Distribution of active (RPP ≥138) and inactive sequences (RPP <138) in naive ESCs in ChIP-STARR-seq regions of the Core module (top), Extended module (middle), or in regions that were inactive in primed ESCs. The p-value calculated by the χ^2 test is indicated.

M) Receiver operating characteristic (ROC) curve illustrating random forest classifier performance. The area under the curve (AUC) and Gini index are reported.

Figure S7, related to Figure 7: Super-enhancers and ChIP-STARR-seq in primed and naive ESCs

A) Super-enhancers in naive H9 ESCs (SEs) were called from H3K27ac ChIP-seq data on enhancers stitched within 12.5kb windows using the ROSE software (Whyte et al., 2013).

B) Scatterplot contrasting SE intensity in naive H9 ESCs (H3K27ac signal divided by input) with ChIP-STARR-seq activity. The Pearson correlation coefficient (*r*) is indicated and the red line represents a generalised additive model fit to the data.

C) Kernel density plots showing the distribution of SE intensity values (H3K27ac signal divided by input) in primed and naive H9 ESCs.

D) Scatterplot contrasting SE intensity values (H3K27ac signal divided by input) in primed and naive H9 ESCs. Regions called as SEs in primed, naive, or both ESCs are indicated in blue, red, or purple, respectively.

E) Comparison between super-enhancers and H3K27ac peaks from H1 ESCs (Hnisz et al., 2013) and this study.

F) Genome browser plots showing two regions captured by our additional BAC-STARR-seq libraries. Shown are annotated genes in these regions, the BAC-covered region itself (in yellow), followed by tracks showing the combined ChIP-seq coverage and RNA over plasmid ratios in primed and naive ESCs.

G) PCR genotyping of H9 wild type (WT) and targeted clones (numbers) that were transfected with Cas9 and gRNAs to delete three different constituents of the *FGFR1* super-enhancer (part A, B and G, see **Figure 7**). Primers used for genotyping are located outside the gRNA targets; wt = wt allele, ko = knockout allele.

H) qRT-PCR analysis of wild type (wt) H9 ESCs or H9 ESCs with a homozygous deletion of *FGFR1* super-enhancer part A, part B or part G for amplicons detecting *FGFR1*, *LETM2, C8ORF86*, *RNF5P1* and *WHSC1L1* (*NSD3*) mRNA. 3 clones per genotype were assessed, and

expression was normalized for *TBP* and one wt set to 1. *** =p<0.001 (2-way ANOVA with Bonferoni post-test), error bars are SD.

I) Violin plots showing the proportion of active plasmids (RPP $\geq$ 138) for 1,159 super-enhancers (SE) compared to normal enhancers (NE) in naive ESCs.

J) Violin plots as in panel I, for SEs and NEs in primed (left) and naive (right) ESCs with super-enhancers based on H3K27ac peaks with d=0kb stitching distance (called using ROSE).

Supplemental Figures

Figure S1
Related to Figure 1

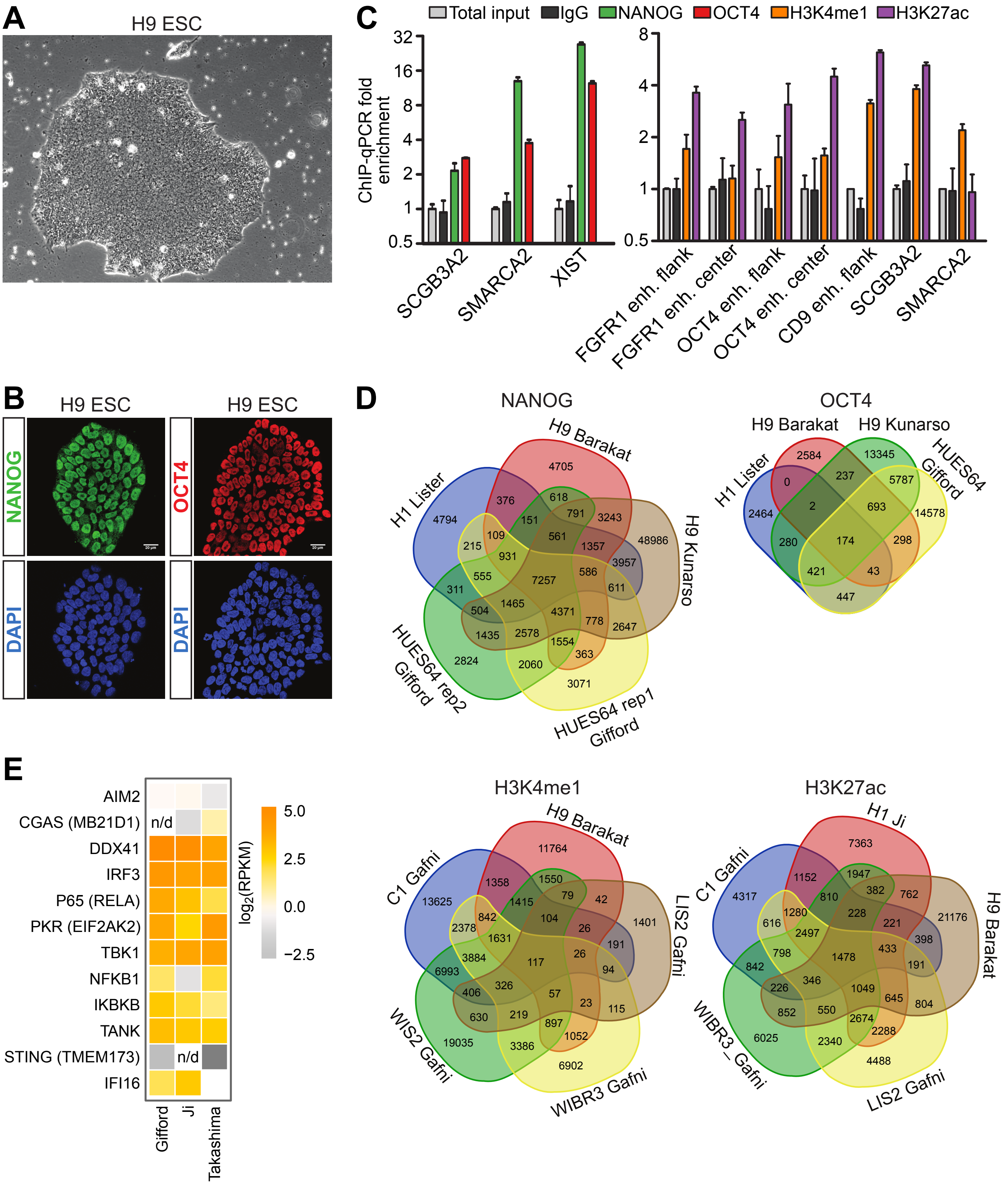

Figure S2
Related to Figure 1

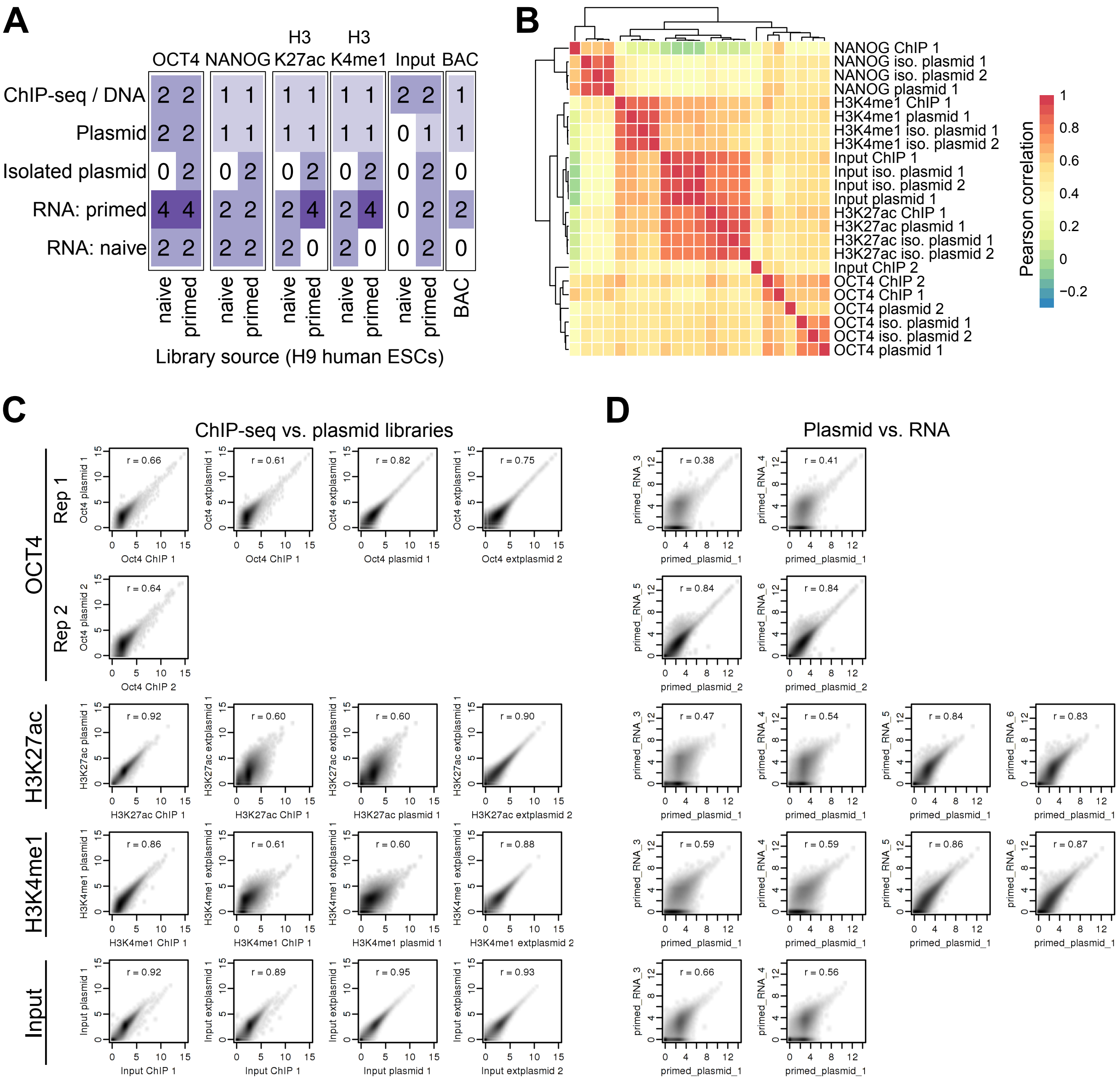

Figure S3
Related to Figure 2

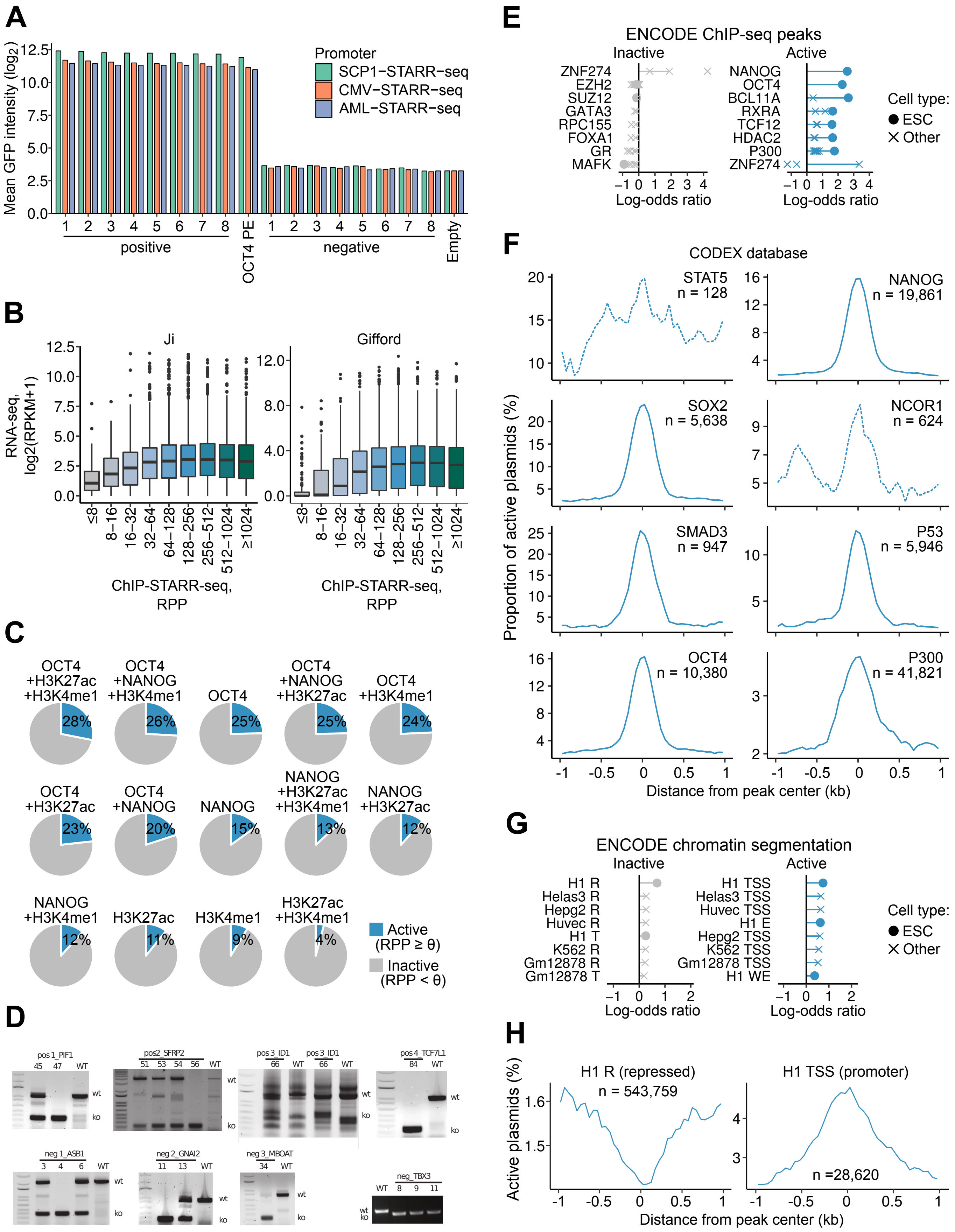

Figure S4
Related to Figure 4

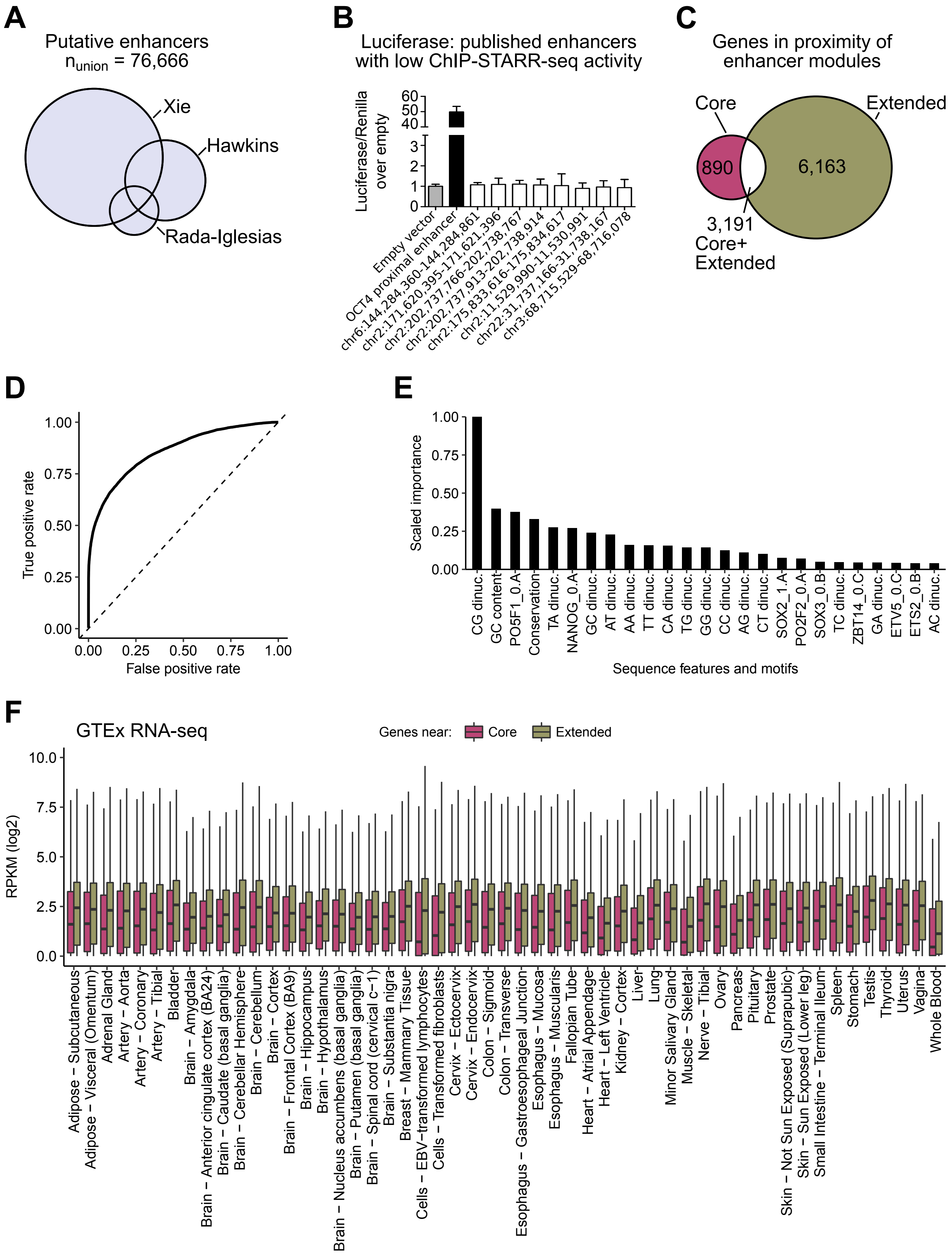

Figure S5
Related to Figure 5

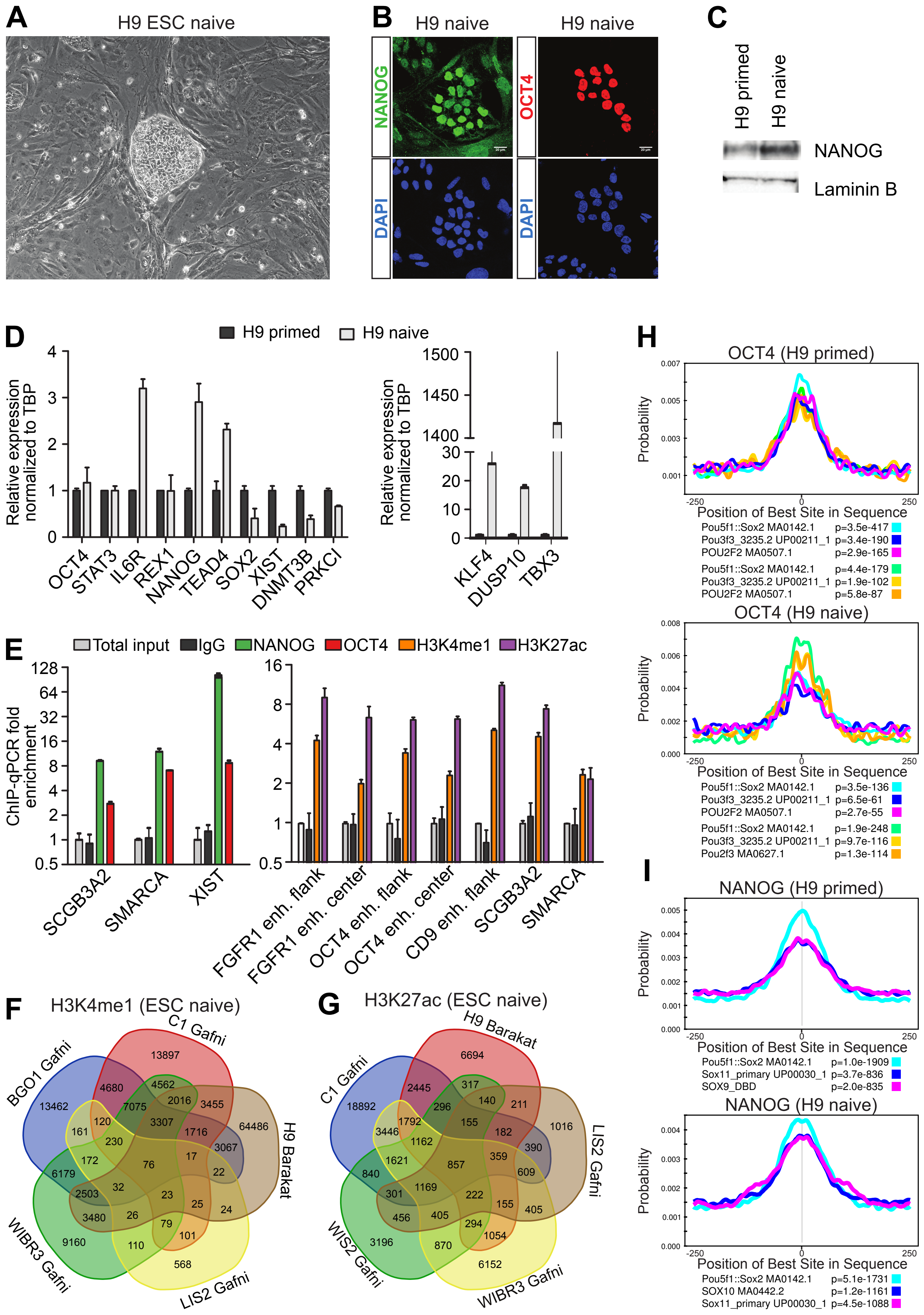

Figure S6
Related to Figure 5

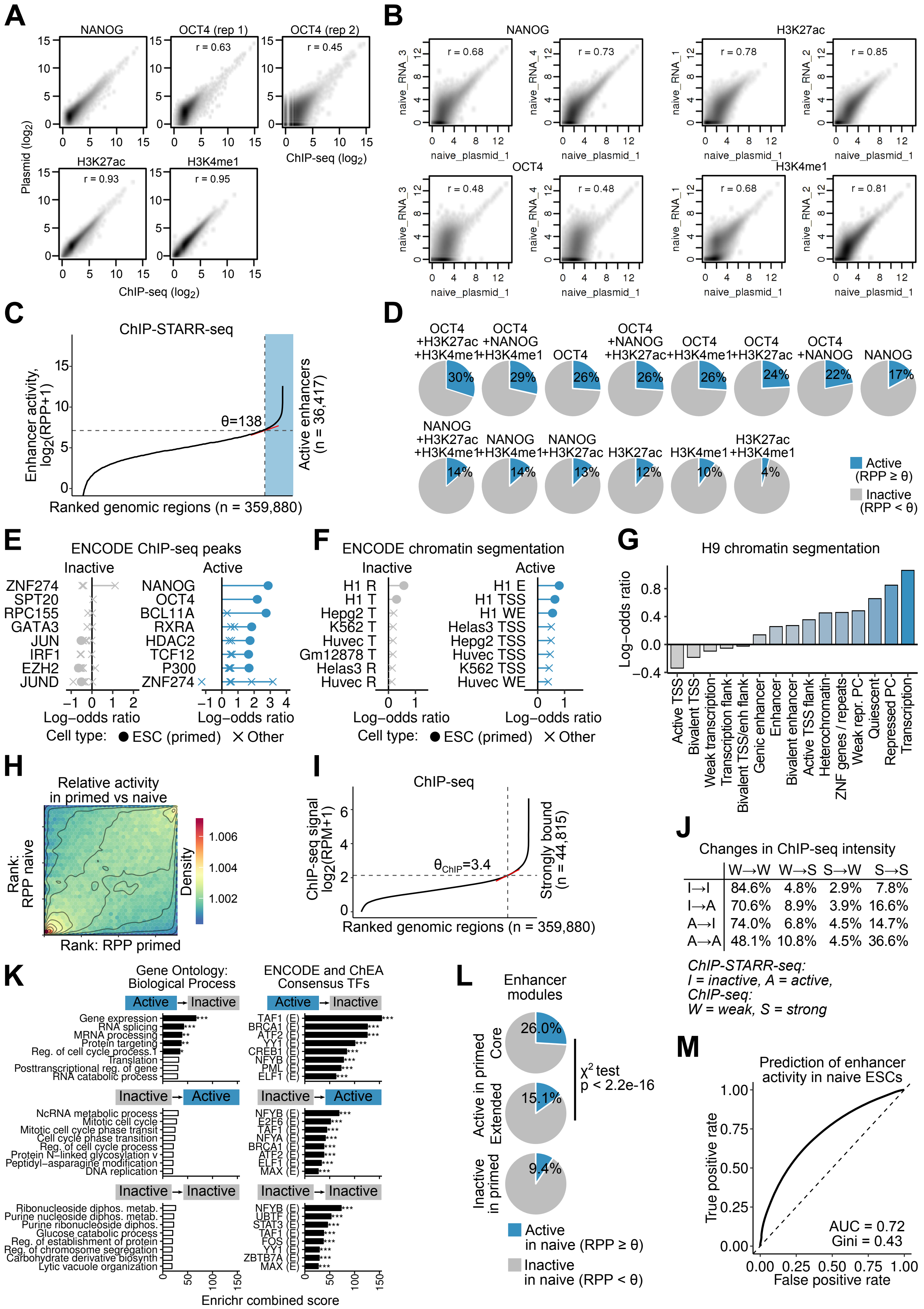

Figure S7
Related to Figure 7

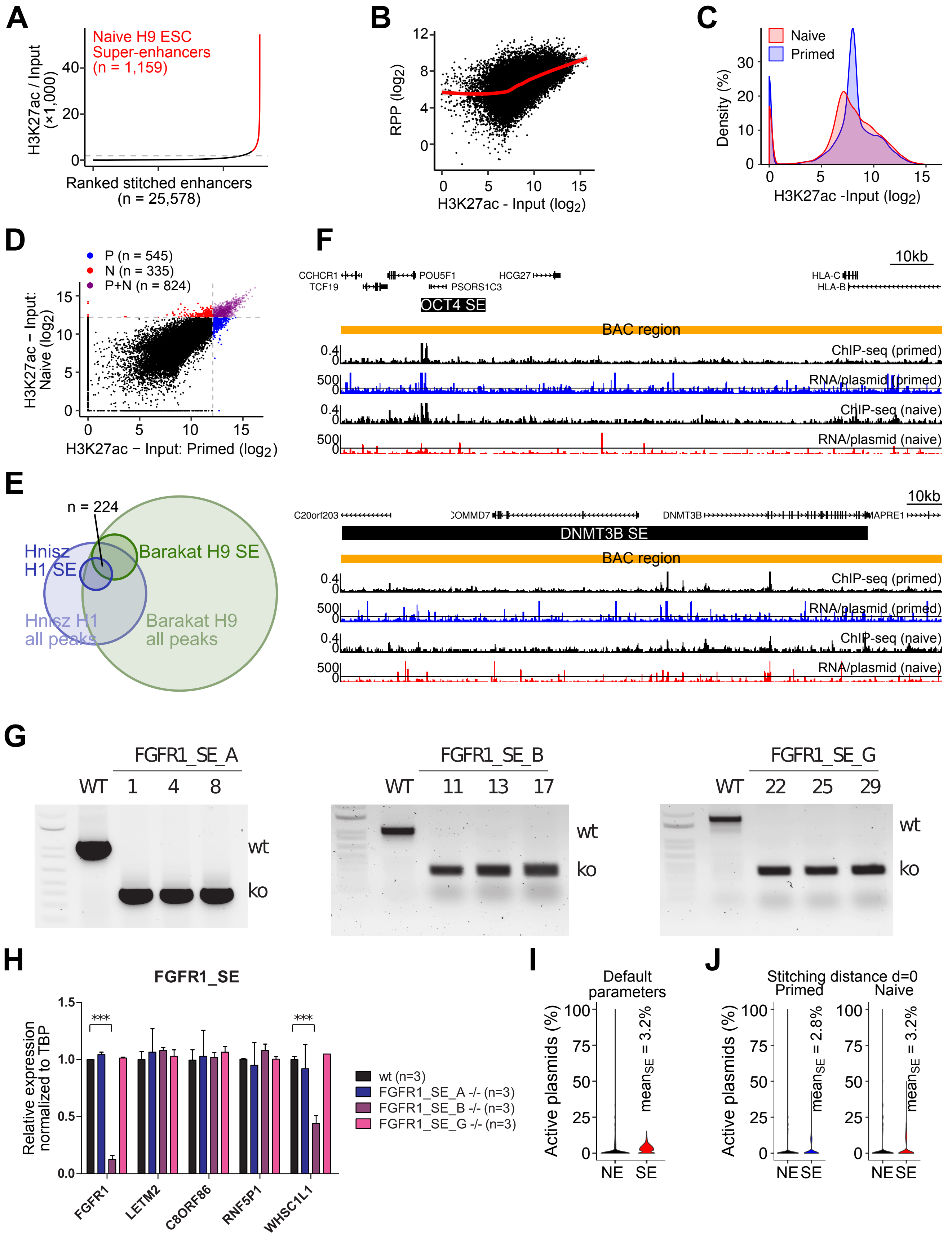

Supplement: Document S2. Article plus Supplemental Information [file mmc8.pdf]
